# HIV Integration Targeting: A Pathway Involving Transportin-3 and the Nuclear Pore Protein RanBP2

**DOI:** 10.1371/journal.ppat.1001313

**Published:** 2011-03-10

**Authors:** Karen E. Ocwieja, Troy L. Brady, Keshet Ronen, Alyssa Huegel, Shoshannah L. Roth, Torsten Schaller, Leo C. James, Greg J. Towers, John A. T. Young, Sumit K. Chanda, Renate König, Nirav Malani, Charles C. Berry, Frederic D. Bushman

**Affiliations:** 1 Department of Microbiology, University of Pennsylvania School of Medicine, Philadelphia, Pennsylvania, United States of America; 2 Medical Research Council Centre for Medical Molecular Virology, Division of Infection and Immunity, University College London, London, United Kingdom; 3 Protein and Nucleic Acid Chemistry Division, Medical Research Council Laboratory of Molecular Biology, Cambridge, United Kingdom; 4 Infectious Disease Laboratory, The Salk Institute for Biological Studies, La Jolla, California, United States of America; 5 Infectious and Inflammatory Disease Center, Burnham Institute for Medical Research, La Jolla, California, United States of America; 6 Department of Family/Preventive Medicine, University of California, San Diego School of Medicine, San Diego, California, United States of America; Duke University Medical Center, United States of America

## Abstract

Genome-wide siRNA screens have identified host cell factors important for efficient HIV infection, among which are nuclear pore proteins such as RanBP2/Nup358 and the karyopherin Transportin-3/TNPO3. Analysis of the roles of these proteins in the HIV replication cycle suggested that correct trafficking through the pore may facilitate the subsequent integration step. Here we present data for coupling between these steps by demonstrating that depletion of Transportin-3 or RanBP2 altered the terminal step in early HIV replication, the selection of chromosomal sites for integration. We found that depletion of Transportin-3 and RanBP2 altered integration targeting for HIV. These knockdowns reduced HIV integration frequency in gene-dense regions and near gene-associated features, a pattern that differed from that reported for depletion of the HIV integrase binding cofactor Psip1/Ledgf/p75. MLV integration was not affected by the Transportin-3 knockdown. Using siRNA knockdowns and integration targeting analysis, we also implicated several additional nuclear proteins in proper target site selection. To map viral determinants of integration targeting, we analyzed a chimeric HIV derivative containing MLV *gag*, and found that the gag replacement phenocopied the Transportin-3 and RanBP2 knockdowns. Thus, our data support a model in which Gag-dependent engagement of the proper transport and nuclear pore machinery mediate trafficking of HIV complexes to sites of integration.

## Author Summary

HIV continues to be responsible for approximately two million deaths worldwide each year. As part of the viral replication cycle, the viral cDNA is transported through the nuclear pore into the nucleus where it integrates into the host cell genome. HIV integrates non-randomly, likely choosing integration sites within the host chromosomes that best enable the viral genes to be expressed and, ultimately, progeny virus to be produced. HIV uses host factors to guide its selection of integration sites. Here we demonstrate that components of the nuclear trafficking and nuclear pore machinery are required for HIV to achieve its normal pattern of integration sites. This finding suggests that passage of the virus through the nuclear pore into the nucleus is coupled to downstream integration events and enables the virus to achieve its final position within the host genome. Our study provides new insights into two important steps of the HIV replication cycle and suggests possible new targets for anti-retroviral drugs.

## Introduction

To complete the early steps of infection, retroviral preintegation complexes (PICs) must access the nucleus of the infected cell and integrate the viral cDNA into host chromatin. Gammaretroviruses such as MLV require nuclear envelope breakdown during mitosis to access cellular chromosomes and complete integration [1], [2]. In contrast, lentiviruses such as HIV can enter the nucleus in non-cycling cells, presumably by traversing the nuclear pore [3]–[5].

Passage through the pore is likely a preferred route of nuclear entry for HIV-1 even in dividing cells – several components of the nuclear pore are required for efficient infection of dividing cells, even though PICs might access the nucleus during nuclear breakdown in mitosis [6]–[11]. Moreover, in infections initiated during interphase, integration occurs before mitosis, while integration in cells infected just prior to mitosis is delayed until the following interphase [12]. These data suggest that the steps of HIV import through the nuclear pore may be coupled to subsequent integration. In support of this hypothesis, König and colleagues found that in dividing cells depleted of some nuclear pore factors or karyopherins, HIV DNA entered the nucleus but did not integrate efficiently [7]. Thus the route of nuclear entry may influence subsequent integration, and the pore may provide the preferred route even in dividing cells.

Retroviral integration is known to be modulated by several host components. Integration target site selection is guided by the genomic environment of the integration acceptor site [13]–[18]. Lentiviruses such as HIV show a preference for integration in active transcription units, which may promote efficient expression after integration [13], [19]–[21]. Gammaretroviruses such as MLV show a preference for integration near gene 5′ ends and CpG islands [13]–[15]. Target site preferences of HIV integration are due in part to tethering by a host chromatin binding protein, Ledgf/p75 (product of the PSIP1 gene), which binds lentiviral IN [22], [23] and mediates IN-chromatin binding [24], [25]. In the absence of Ledgf/p75, HIV integration is severely compromised and integration in transcription units is diminished [26]–[28]. Recently, the tethering model for Ledgf/p75 function was bolstered by the finding that fusion proteins containing the IN-binding domain of Ledgf/p75 fused to alternative chromatin binding domains retargeted lentiviral integration efficiently [29]–[31].

Here we analyze host factors identified in genome-wide siRNA screens [6]–[8] and find links between transport into the nucleus and subsequent integration targeting. We chose factors whose depletion, like that of Ledgf/p75, led to an infection block at nuclear entry or integration. We initially surveyed effects of knocking down expression of ten genes, then focused on two of them, TNPO3 and RANBP2, which encode components of the nuclear pore and import machinery. TNPO3 encodes Transportin-3, a karyopherin [32] that has been shown to be required for import of HIV PICs into the nucleus in cycling cell lines and macrophages [6], [7], [9]. RanBP2 (originally named Nup358), is a large cyclophilin-related nuclear pore protein involved in the Ran-GTPase cycle that orchestrates much of nuclear import and export [33], and is also required for import of HIV PICs [7]. Recently, Lee and colleagues isolated a capsid mutant (N74D) [34] that bypassed the requirement for Transportin-3 and RanBP2, but acquired a requirement for other nuclear pore factors. HIV capsid had previously been suggested to be a viral determinant of nuclear entry [35] and these data suggest a possible direct interaction of capsid with Transportin-3 and RanBP2.

Using RNA interference, we reduced the expression of candidate genes, confirmed that HIV titer was reduced as a result, and then investigated the distribution of integration sites in the human genome using DNA bar coding and 454/Roche pyrosequencing. As controls, we studied infections and targeting by MLV. We also studied integration targeting by a derivative of HIV containing the *gag* gene (encoding the capsid structural proteins) of MLV. We found that depletion of Transportin-3 and RanBP2 resulted in marked alterations in the distribution of HIV integration sites, providing a link between nuclear entry and integration targeting. MLV integration patterns were not altered in Tranportin-3 knockdowns, and substitution of MLV Gag into HIV phenocopied the effects of the knockdowns. Several additional host gene products were also identified as candidate members of the pathway. Thus we can begin to specify a "railroad track" through the nuclear pore to favored sites of HIV DNA integration.

## Results

### Surveying integration site distributions after siRNA knockdown

**Figure 1:**
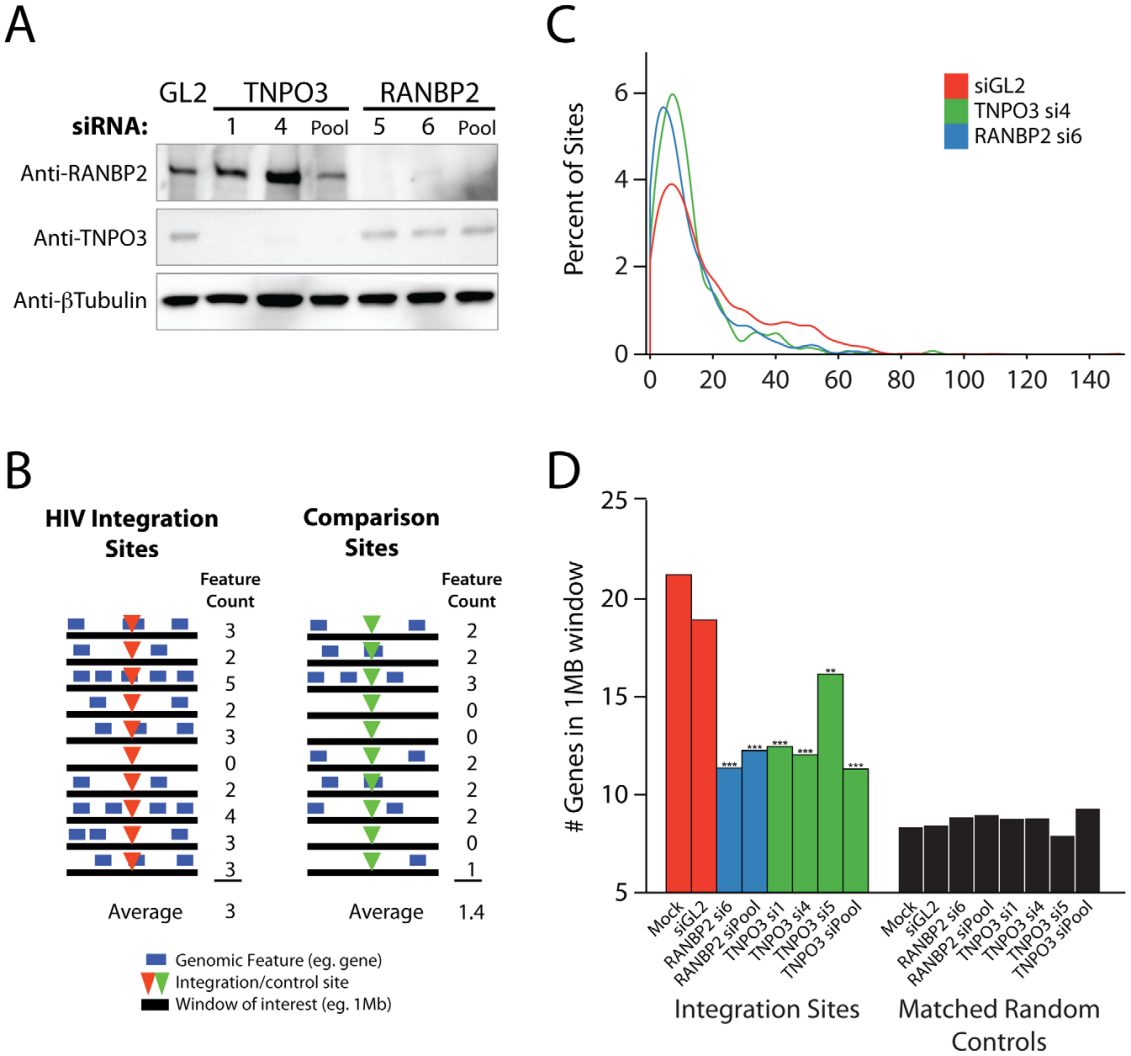
Effects of siRNA treatments on HIV integration in gene dense regions. Cells were transfected with individual siRNAs or an siRNA pool of four siRNAs targeting the same gene as indicated, and infected 48 hr later for an additional period of 48 hr prior to integration site analysis. (A) Reduction in Transportin-3 and RanBP2 protein levels after RNAi. Protein abundance was measured at the time of infection by Western blot with β-tubulin as a loading control. For comparison, protein levels are shown in cells treated with an siRNA against firefly luciferase (GL2), a gene not found in HEK-293T cells. (B) Overview of the approach for integration site analysis. The number of genomic features of interest (blue bars), such as transcription units, is tabulated within genomic intervals (black bars) surrounding integration sites (red arrowheads) or computationally-generated matched random control sites (green arrowheads). The average number of times the genomic feature occurs within that window can be compared across datasets. (C) Histogram indicating distribution of integration sites with respect to gene density. Cells were transfected with individual siRNAs and infected as above. Sample names in legend indicate the gene targeted followed by the individual siRNA number. The number of genes in 1 Mb windows surrounding each integration site was counted as in 1B. Integration sites in each dataset were binned (along the X-axis) according to the number of genes within 1 MB interval surrounding each site. Curves were computed from histogram plot using Gaussian kernal density estimates. (D) Barplot of the average number of RefSeq genes in 1 Mb windows surrounding sites of HIV integration or computationally generated matched random controls. Mock transfected cells (no RNAi) and cells treated with the siRNA targeting luciferase GL2 (siGL2) are shown as controls. Asterisks denote significant difference from control GL2 siRNA treated cells as determined by the nonparametric Mann–Whitney test (*P<0.05; **P<0.01; ***P<0.001).

We initially analyzed 10 genes previously implicated as HIV cofactors at or near the integration step to determine whether they had effects on integration targeting (Table S1). We selected NUP98 [7], [11], MAP4 [6], [7], IK [7], ANAPC2 [7], [8], PRPF38A [7], RANBP2 [6], [7], SNW1 [7], and TNPO3 [6], [7] from siRNA screens, and two other genes, WDR46 and WDHD1, the products of which bind Ledgf/p75 in yeast two-hybrid screens (unpublished data). For each gene, we tested several different siRNAs in HEK-293T cells. Reduction of mRNA levels was confirmed by quantitative RT-PCR (Figure S1), and we assessed inhibition of infection by a VSVG-pseudotyped GFP reporter virus, as defined as percent of cells expressing the GFP marker 48 h after infection (Figure S2), as well as toxicity of the siRNAs (Figure S3). Selected knockdowns were verified by Western blot (Figure S4 and Figure 1A).

This initial scan showed robust effects on infection efficiency for the nuclear import factors Transportin-3 and RanBP2, confirming observations from earlier studies [6], [7], [9], [34]; therefore, these genes were studied in detail as described in the following sections. Results for Transportin-3 and RanBP2 have been corroborated by further studies using stable knockdowns with shRNAs in HeLa cells that achieved efficient reductions in mRNA levels (Schaller *et al.*, submitted). The remaining 8 genes were also analyzed for integration targeting using our high throughput pipeline. We return to findings for this group of genes at the end of the Results.

### HIV integration site selection is modified by depletion of Transportin-3 and RanBP2

Having confirmed that knockdown of Transportin-3 and RanBP2 reduced the efficiency of HIV infection (Figure S2), we examined the effect of these factors on integration site selection using ligation-mediated PCR and 454-pyrosequencing as previously described [36]. Recovered genomic sequences were mapped to the human genome draft hg18. Association of integration sites with genomic features was then assessed (e. g. Figure 1B).

In the human genome, many types of features are linked–for example, gene dense regions are rich in CpG islands and DNAseI sites, high in G/C content, and rich in highly expressed genes [37], [38]. As a first step in illustrating the results, we present integration site distributions as a function of gene density. In cells depleted of Transportin-3 or RanBP2, the distribution of HIV integration sites was altered towards regions of lower gene density in comparison to control cells treated with siGL2, which targets firefly luciferase GL2, a gene not found in the HEK-293T cells (Figure 1C). The trend towards integration in less gene dense regions was significant for both RANBP2 and TNPO3 knockdowns (p<0.001, see below). There was no evidence of a bimodal distribution integration sites with respect to gene density, which would have suggested knockdown of the factors in only a portion of the cells (Figure 1C).

The average gene density in a one megabase window surrounding integration sites in cells depleted of either Transportin-3 or RanBP2 is plotted in Figure 1D. For comparison, matched random control sites within the human genome were computationally generated and are shown in black (described in [14], [18] and Text S1). The average gene density at integration sites in the RANBP2 and TNPO3 knockdown cells was reduced compared to cells treated with siGL2, though it remained higher than would be expected for random integration. Thus integration in gene dense regions is promoted in part by RanBP2 and Transportin-3. As a control for the fact the knockdowns diminished infection, we investigated whether infections at low MOI altered the distribution of integration sites, but MOI was not found to affect integration targeting detectably (data not shown).

**Figure 2:**
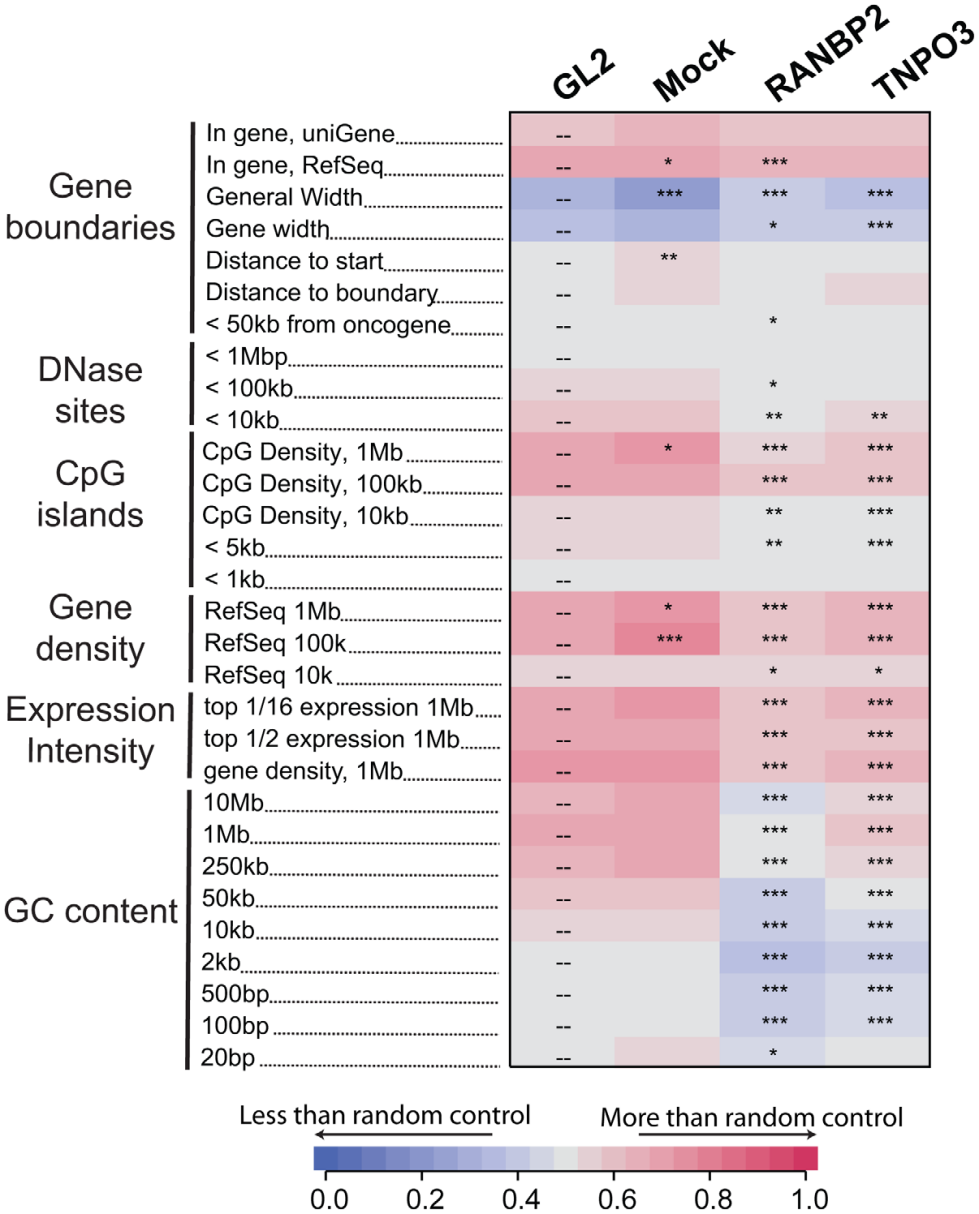
Effects of Transportin-3 and RanBP2 depletion on integration near multiple chromosomal features. Genes targeted by siRNA in infected cells including the control, GL2, are shown above the columns. Mock cells received no siRNA. The genomic features analyzed are shown in the rows and labeled on the left. Relationships between integration frequency and feature density are summarized using ROC curve areas [18], where increasing shades of blue indicate a negative correlation with integration frequency and increasing shades of red indicate a positive correlation with integration frequency relative to matched random control distributions. The control GL2 siRNA set was used for pairwise statistical comparisons (overlay dashes). P values summarizing the significance of the departure from the GL2 control are shown with asterisks (*P<0.05; **P<0.01; ***P<0.001). Note that the asterisks and the heat map summarize different comparisons (to siGL2 and matched random controls, respectively). The base pair values in the row labels indicate the size of the genomic interval used for analysis–often the most appropriate interval is not known, so several different interval sizes are compared. A more detailed guide to the data presented in this figure can be found in Text S1. An interactive version of this figure is available as Figure S5.

Analysis of integration frequency relative to a large collection of genomic features (described in Text S1) showed a common set of changes in both the Transportin-3 and RanBP2 depleted cells relative to the controls (Figure 2 and Figure S5). The reduction in integration in gene dense regions was significant for both TNPO3 and RANBP2 knockdowns when analyzed over multiple genomic intervals of different lengths. Significant differences were also seen when only expressed genes (identified by Affymetrix chip transcriptional profiling) were considered in a similar analysis (labeled “Expression Intensity” in Figure 2). Genomic features that correlate with gene density such as DNase I hypersensitive sites and CpG islands were similarly enriched near control HIV integration sites but less enriched near sites from TNPO3 and RANBP2 knockdown cells. GC-rich regions, normally favored by HIV [13], were disfavored in most window sizes in the Transportin-3 and RanBP2 knockdowns.

By contrast, gene density at integration sites was not significantly affected in Ledgf/p75 knockdowns compared to the control. The GC content and the density of CpG islands within one kb of integration sites actually increased in Ledgf/p75-depleted cells [26]–[28], indicating divergent effects on integration targeting. Integration within genes, which is reproducibly diminished in Ledgf/p75-depleted cells [26]–[28], was not affected by TNPO3 knockdown, and showed only a slight decrease in the RANBP2 knockdown cells. Together these data suggest that Transportin-3 and RanBP2 influence HIV integration targeting relative to a collection of features associated with gene dense regions, and do so in a manner that differs from Ledgf/p75 tethering.

### Effect of Transportin-3 depletion on integration site selection can be partially rescued by expression from an siRNA insensitive TNPO3 allele

**Figure 3:**
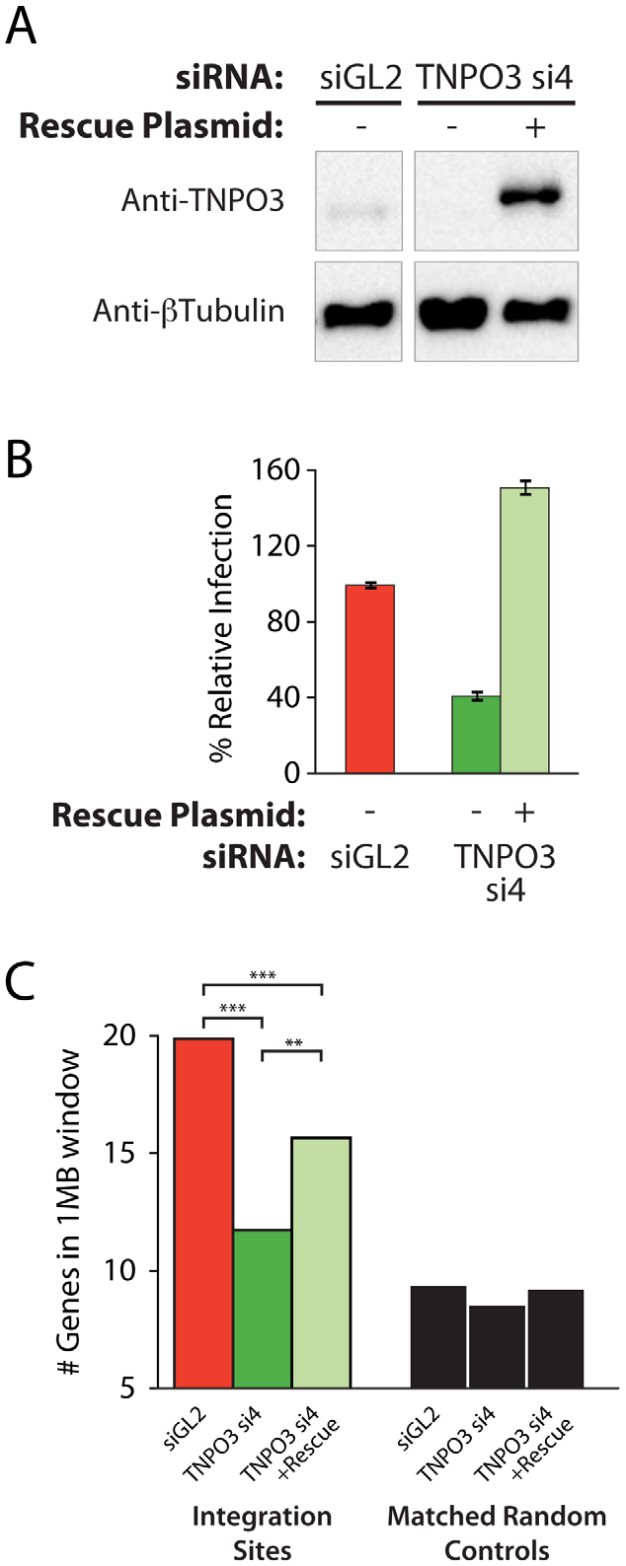
Transfection of a Transportin-3 allele insensitive to TNPO3 si4 restores protein expression, HIV infectivity, and partially restores wild-type HIV integration site distributions. (A) Western blot showing Transportin-3 levels in cells treated with TNPO3 si4 in the presence or absence of the Transportin-3 rescue plasmid. Cells were cotransfected with siRNA and either empty vector plasmid or rescue plasmid encoding siRNA-resistant alleles of Transportin-3 expressed from the CMV promoter and harvested at 48 hr post-transfection for analysis. Transportin-3 is reduced after co-transfection with siRNA and empty vector, and overexpressed after co-transfection with siRNA and rescue plasmid. Endogenous levels of Transportin-3 are shown in cells transfected with the control siRNA targeting GL2 and an empty vector. (B) HIV infection in cells treated with TNPO3 si4 in the presence or absence of the Transportin-3 rescue plasmid. Cells were co-transfected as above. 48 hr after transfection cells were infected with a VSVG-pseudotyped HIV-1 vector carrying a GFP reporter. At 48 hpi cells were harvested and the percent of cells expressing GFP was determined by flow cytometry. The Y-axis shows relative infection compared to infection in the control (GL2 siRNA + empty vector-transfected) cells. (C) Average gene density in 1 Mb windows surrounding HIV integration sites in cells depleted or rescued for Transportin-3 expression. Asterisks denote significant differences as determined by the Mann–Whitney test (*P<0.05; **P<0.01; ***P<0.001).

Multiple different siRNAs directed against TNPO3 and RANBP2 mRNAs yielded similar effects on integration targeting that were not observed in control knockdowns, indicating that off-target effects were unlikely to explain the observed alterations in integration targeting. As an additional control, we analyzed complementation of the Transportin-3 depletion using a plasmid-encoded siRNA-insensitive allele generated by site-directed mutagenesis of the siRNA target sequence. The RANBP2 coding region is very large (11,711 bp), and so rescue experiments were not attempted for this factor. Co-transfection of the resistant Transportin-3 expression vector with the corresponding siRNA resulted in overexpression of Transportin-3 and restored HIV infection, increasing reporter virus GFP expression above control levels (Figure 3a and b).

We observed an increase in gene density near integration sites in knockdown cells co-transfected with the siRNA-insensitive TNPO3 allele compared to vector-only controls (Figure 3c and Figure S6). The average number of genes within 1 Mb of HIV integration sites increased from 11 (in the presence of TNPO3 si4 and an empty vector) to 14 when Transportin-3 expression was rescued (p<0.01, Figure 3c). The effect of knockdown in the presence and absence of rescue on additional genomic features is described in Text S2. It is unclear why restoring Transportin-3 protein levels did not fully rescue the integration defect, but this result may be due to the abnormally high levels of Transportin-3 expressed from the siRNA-resistant construct. Nevertheless, these data support the idea that off-target effects of the TNPO3 siRNA do not account for the phenotypes observed.

### Transportin-3 depletion has no detectable effect on gene density surrounding MLV integration sites

**Figure 4:**
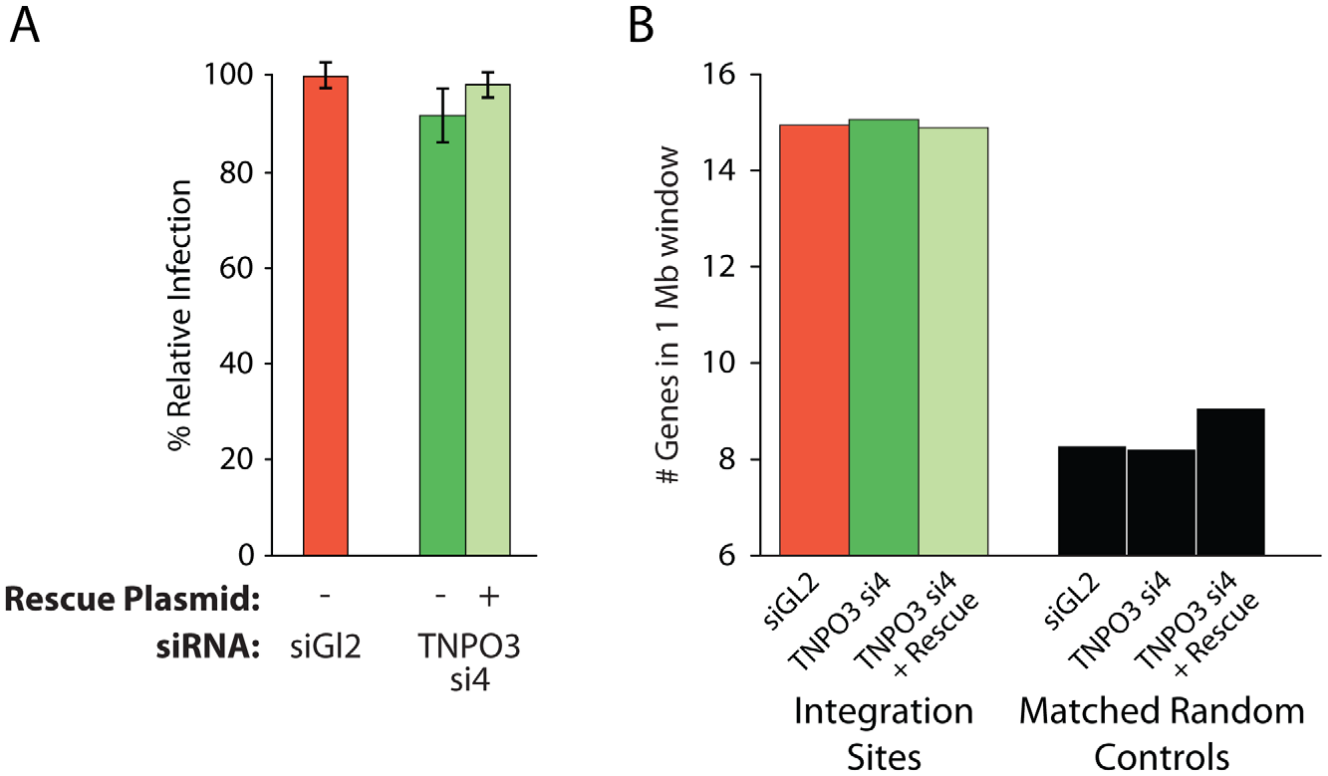
Depletion of Transportin-3 does not alter MLV integration targeting. (A) Infection levels of MLV in cells co-transfected with the control siRNA to GL2 plus an empty vector, TNPO3-si4 plus an empty vector, or TNPO3 si4 plus a vector encoding the siRNA-resistant Transportin-3 allele. (B) Average gene density in 1 Mb windows surrounding MLV integration sites in cells depleted or rescued for Transportin-3 expression. No sets showed significant differences from GL2-treated cells as determined by the Mann–Whitney test.

As a control, we tested whether MLV integration, which requires cell division for infection and is not dependent on Transportin-3 [7], [39], showed altered integration targeting in the Transportin-3-depleted cells. We found that treatment with siRNA targeting TNPO3 mRNA, either in the presence or absence of the rescue plasmid, did not affect MLV infection efficiency (Figure 4a). We sequenced MLV integration sites from knockdown and control cells (Table S1), and found no significant changes in MLV integration frequency in gene dense regions (Figure 4b), within transcription units, or with respect to GC content (data not shown). These data indicate that Transportin-3 depletion does not affect MLV integration targeting as it does for HIV.

### Other nuclear factors may participate in directing integration to gene dense regions

**Figure 5:**
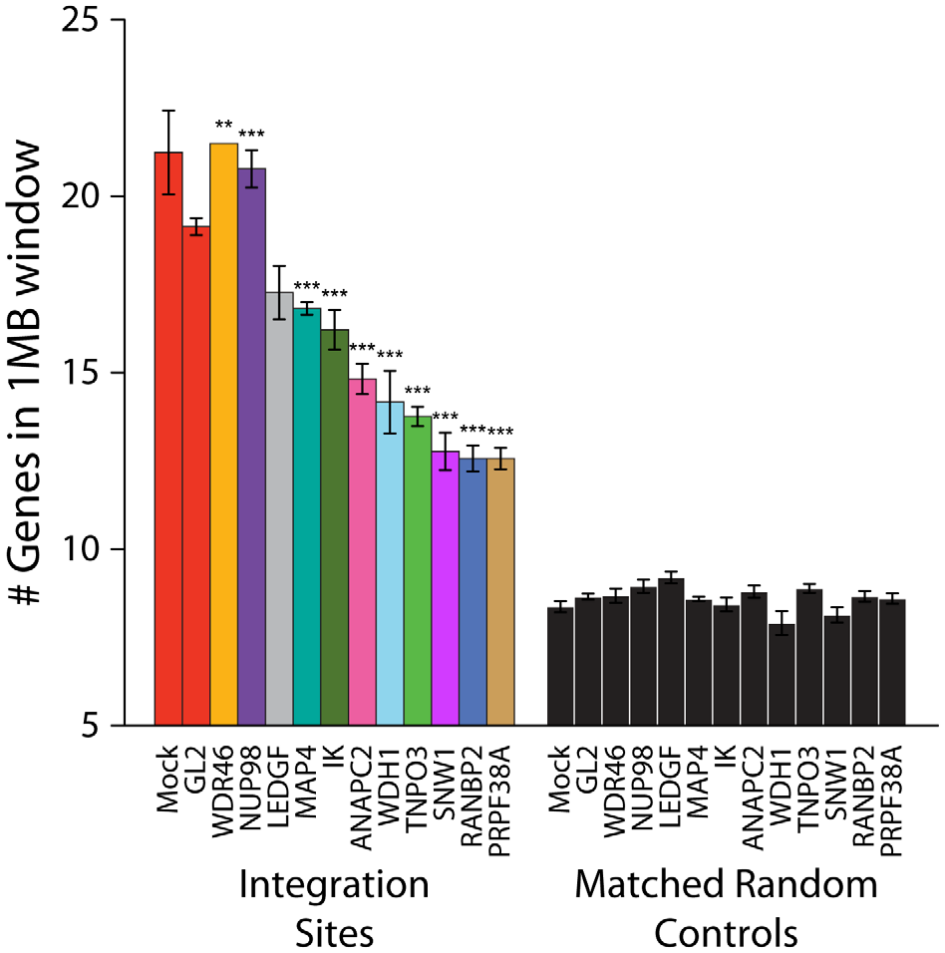
Depletion of additional host factors and their effects on HIV integration in gene dense regions. Integration sites were isolated from cells treated with siRNAs targeting the indicated genes. The average numbers of RefSeq genes in 1 Mb windows surrounding integration sites are shown. Data for a given gene knockdown is the average over multiple siRNA knockdowns using different siRNAs and pools of siRNAs targeted to the same gene, except for Mock, control siRNA (GL2), and LEDGF knockdown conditions for which single treatments were used. Asterisks denote significant difference from control GL2 siRNA-treated cells as determined by the Mann–Whitney test (*P<0.05; **P<0.01; ***P<0.001). Error bars represent standard error for biological replicates. For LEDGF only one dataset was available.

Integration site data sets were also acquired for cells treated with siRNAs for NUP98, MAP4, IK, ANAPC2, PRPF38A, SNW1, WDR46 and WDHD1 (Table S1). For many of these, considerable toxicity was detected (Figure S3). Thus interpretation of integration targeting results for these factors is more tentative than for Transportin-3 and RanBP2. Data sets were analyzed for their association with gene density as for Transportin-3 and RanBP2 (Figure 5). Knockdown of several of the factors (ANAPC2, SNW1, PRPF38, WDH1, and IK) led to decreased integration in gene dense regions. MAP4 depletion was also seen to modestly decrease integration preference for gene dense regions in some experiments. For two of these genes, SNW1 and ANAPC2, we confirmed that although MLV infection is diminished in the knockdowns as previously noted [7], the gene density at MLV integration sites is unchanged (Figure S7), suggesting that, like Transportin-3, the factors encoded by these genes are potentially involved in targeting pathways specific for HIV. By contrast, gene density at integration sites in cells stably depleted of Ledgf was not significantly decreased compared to the siGL2 control.

**Figure 6:**
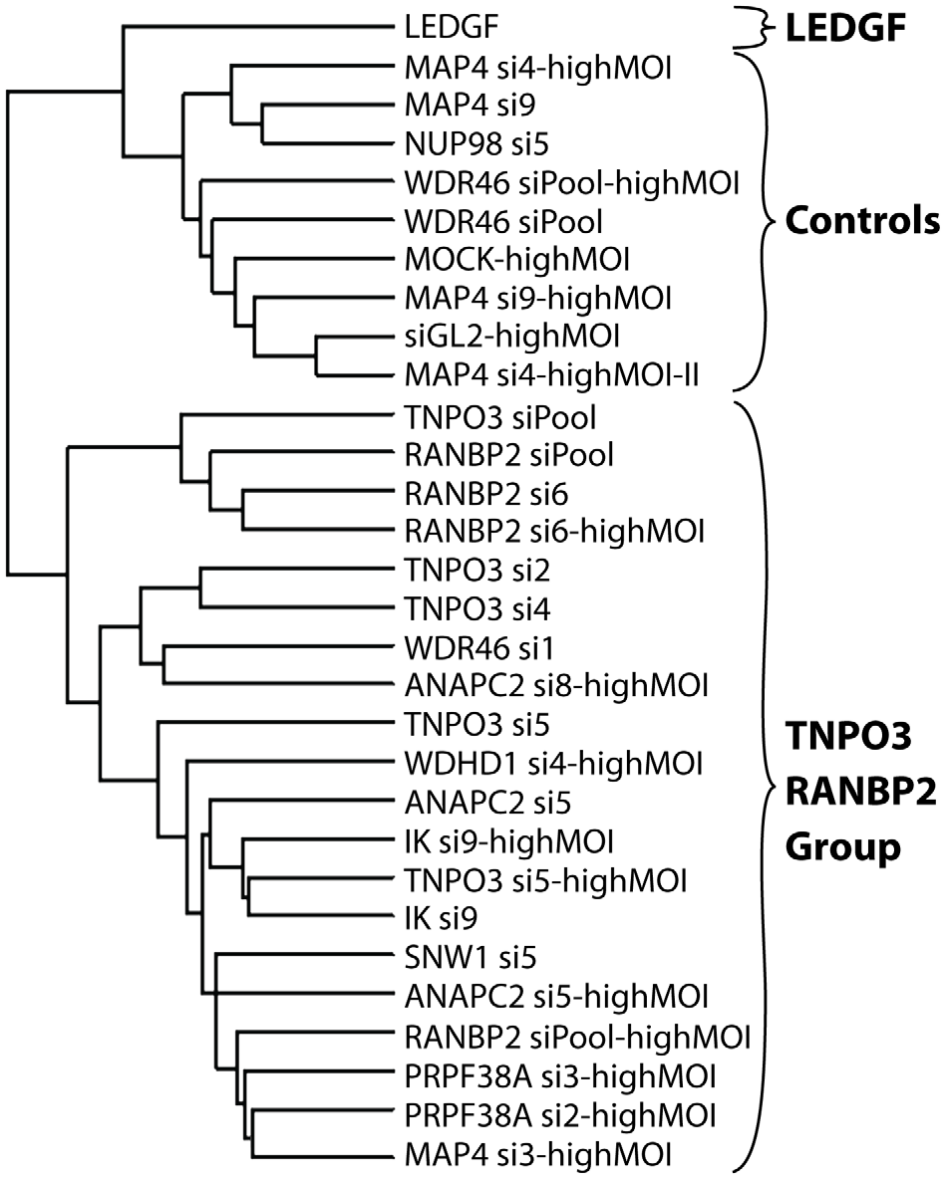
Dendrogram showing clustering of integration site data sets from knockdowns of Transportin-3, RanBP2, and several additional factors. Only sets containing at least 200 integration sites were used for the analysis. A conditional logit model was used to cluster integration sites data sets based on annotation of in or out of annotated transcription units, gene density, expression density, CpG islands, G/C content, nearby oncogenes, and local sequence features (Text S3). Sets were clustered based on their overall similarity in a pairwise analysis. The "Control" cluster is so named because it contains the Mock and siGL2 control data sets. Branch labels indicate the siRNA used for the analysis, and indicates the name of the targeted gene (e.g. TNPO3 si4). Infections were performed using enough HIV vector stock to infect 30–60% of untreated cells except where marked as “highMOI” where 90–100% of untreated cells were infected.

For those knockdowns where we could sequence at least 200 integration sites, the global integration site patterns were investigated by assessing integration frequency relative to many genomic features for each knockdown, and the patterns were clustered using a conditional logit model to conduct pairwise comparisons of the datasets (details are in Text S3). The dendrogram in Figure 6 shows that the controls clustered in a group separate from Transportin-3 and RanBP2 knockdowns. Data sets for several additional gene knockdowns clustered in the TNPO3/RANBP2 group, including IK, ANAPC2, SNW1, WDHD1 and PRPF38A. For MAP4 and WDR46 different siRNAs fell in different groups, and so these have an indeterminate effect. Thus the IK, ANAPC2, SNW1, WDHD1 and PRPF38A genes encode candidates for additional factors acting in the same pathway with Transportin-3 and RanBP2. The Ledgf/p75 knockdown was an outlier in the control cluster. This is consistent with Ledgf/p75 knockdown leading to effects not seen in depletion of RanBP2, or Transportin-3. For this analysis we investigated both low MOI (30–60% infected wild type cells) and high MOI (90–100% infected cells) infections. In most cases the MOI made no difference on the overall position of a knockdown within the tree, suggesting that the roles of the factors are not saturable under the conditions tested.

### HIV *gag* is a determinant of integration targeting to gene dense regions

**Figure 7:**
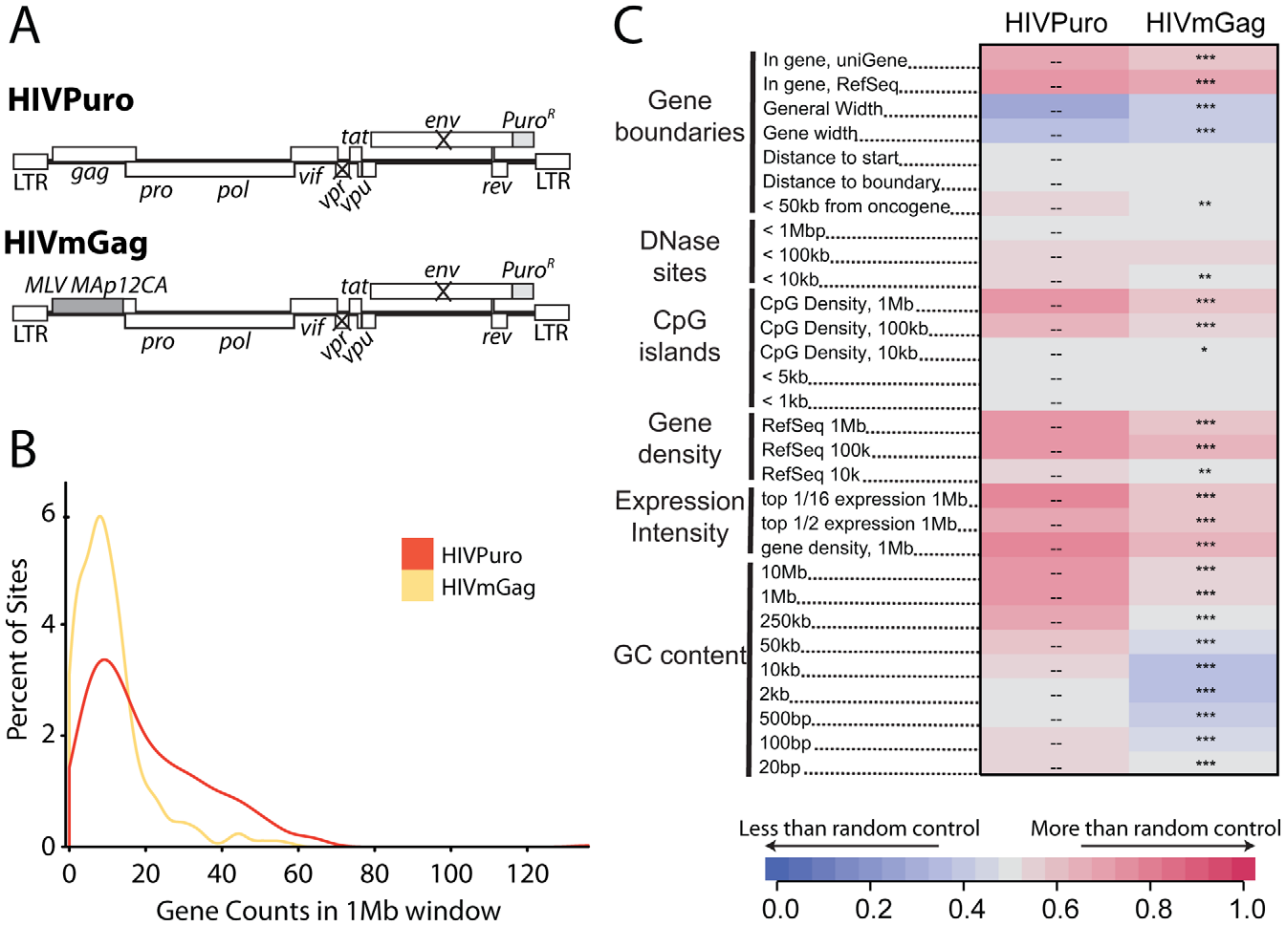
A chimeric derivative of HIV containing MLV *gag* (HIVmGag) shows reduced integration frequency in gene dense regions. (A) Genetic map of HIV proviruses containing wild type *gag* (HIVPuro) or a chimera encoding MLV Gag (MA, p12, and CA) in place of HIV MA and CA (HIVmGag). Both viruses have inactivated *vpr* and *env* and a puromycin selectable marker in place of *nef*. (B) Histogram indicating distribution of HIVPuro and HIVmGag integration sites with respect to gene density measured in 1 Mb intervals surrounding integration events. Data is plotted as in Fig. 1C and curves are computed using Gaussian kernel density estimates. (C) Genomic heatmap of HIVPuro and HIVmGag datasets. Significant differences are shown by asterisks (*p<0.05; **p<0.01; ***p<0.001). Annotations at the left of the heat map are as in Figure 2 and described in Text S1. An interactive version of this figure is available as Figure S8.

We previously studied integration targeting in HeLa cells using HIV chimeras containing MLV *gag*, MLV *IN*, or both, in place of their HIV counterparts [40]. We found that MLV IN was a dominant determinant of MLV-like integration, resulting in integration near transcription start sites by HIV derivatives containing MLV IN. Similar chimeric viruses have been used to show that HIV capsid is a dominant viral determinant of HIV nuclear entry in non-dividing cells [41]. Recently, Lee and colleagues [34] suggested that the HIV CA protein might determine the interactions between HIV PICs and nuclear pore components. These findings led us to reinvestigate integration targeting by the HIV chimera containing MLV *gag* in place of HIV *gag* (HIVmGag; Fig. 7A) [40]. We found that HIVmGag showed a shift in distribution of integration sites towards less gene dense regions compared to the unmodified control (Figure 7B). The average number of genes within 1 MB of HIVmGag integration sites was 11 as compared to 20 for the unmodified HIV control (A Chi square test over ranked comparisons of gene density values between the two sets attains a p value of <2.22–16). A comparison over many genomic features (Figure 7C and Figure S8) showed a pattern of HIVmGag integration similar to that seen for HIV in Transportin-3 and RanBP2 depleted cells (compare Figure 2), including reduced density of genes, CpG islands, DNase I hypersensitive sites and reduced GC content surrounding integration sites. Thus substitution of HIV *gag* with MLV *gag* phenocopied the TNPO3 and RANBP2 knockdowns.

### Knockdowns of RANBP2 or TNPO3 do not cause HIV to favor integration near transcription start sites

**Figure 8:**
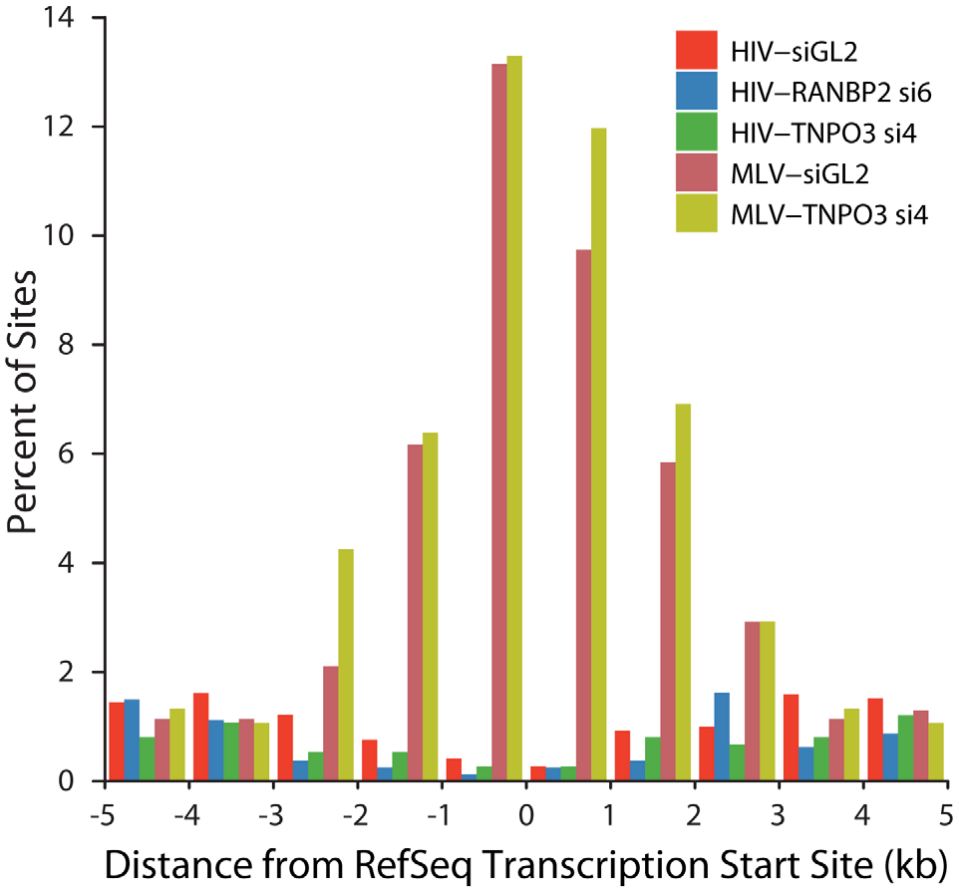
HIV and MLV integration patterns at transcription start sites are unaffected by knockdown of TNPO3 or RANBP2. The percent of integration sites within the indicated genomic distances (kb) from the transcription start site (RefSeq genes) is plotted for each dataset. Sample names indicate the VSVG-pseudotyped viral vector used (HIV or MLV) followed by the cell treatment (either control siGL2 or gene-specific siRNA used).

A model to explain the altered integration site patterns of HIV in TNPO3 or RANBP2 knockdowns is that in the absence of these pore proteins the HIV PIC accesses chromatin during nuclear breakdown during mitosis. MLV employs such a mechanism for nuclear entry, so we wondered whether the HIV integration site distributions in the knockdowns might resemble the normal pattern for MLV. We asked whether HIV integration in cells knocked down for TNPO3 and RANBP2 shows the most characteristic feature of MLV integration, favored integration near transcription start sites (Figure 8). We found that HIV in the knockdowns disfavors transcription start sites, paralleling HIV integration in unmodified cells. MLV showed strongly favored integration in transcription start sites in the 293T cells studied, and in 293T cells knocked down for TNPO3. We conclude that obstructing the normal HIV pathway of integration by knocking down RANBP2 or TNPO3 does not result in an MLV-like integration targeting pattern. This is consistent with the observation that IN is the dominant determinant of MLV like integration patterns at transcription start sites for chimeric viruses where HIV IN is replaced with MLV IN [40], [41].

## Discussion

Here we report that depletion of Transportin-3 and RanBP2 by RNAi affects the downstream choice of targets for HIV DNA integration, providing evidence for coupling of the nuclear translocation and integration steps. As others have noted, Transportin-3 has little or no effect on infection efficiency of MLV [6], [7], [9], which is not thought to traverse the nuclear pore, and we report that Transportin-3 did not affect integration targeting by MLV. Replacing HIV *gag* with MLV *gag* phenocopied the effects of the Transportin-3 and RanBP2 knockdowns on HIV integration targeting. These findings support a model in which HIV Gag proteins interact with Transportin-3 and RanBP2 to mediate HIV integration targeting to chromosomal regions rich in genes and associated features.

We found that depletion of several additional factors previously shown to be required for efficient integration also resulted in HIV integration targeting patterns similar to those seen in Transportin-3 and RanBP2 depleted cells. These factors include a component of the anaphase promoting complex (ANAPC2) splicing factors (SNW1 and PRPF38), a WD-repeat protein (WDHD1), and nuclear DNA binding proteins (IK and SNW1). The analysis of some of these was complicated by cell toxicity, and in some cases conflicting results were obtained with different siRNAs, so effects of these factors are less well supported than those of Transportin-3 and RanBP2. It is possible that each of these factors acts in a common pathway with Transportin-3 and RanBP2 to direct integration to regions dense in genes and associated features, though depletion of some of these factors could also alter the synthesis or function of other factors acting more directly.

**Figure 9:**
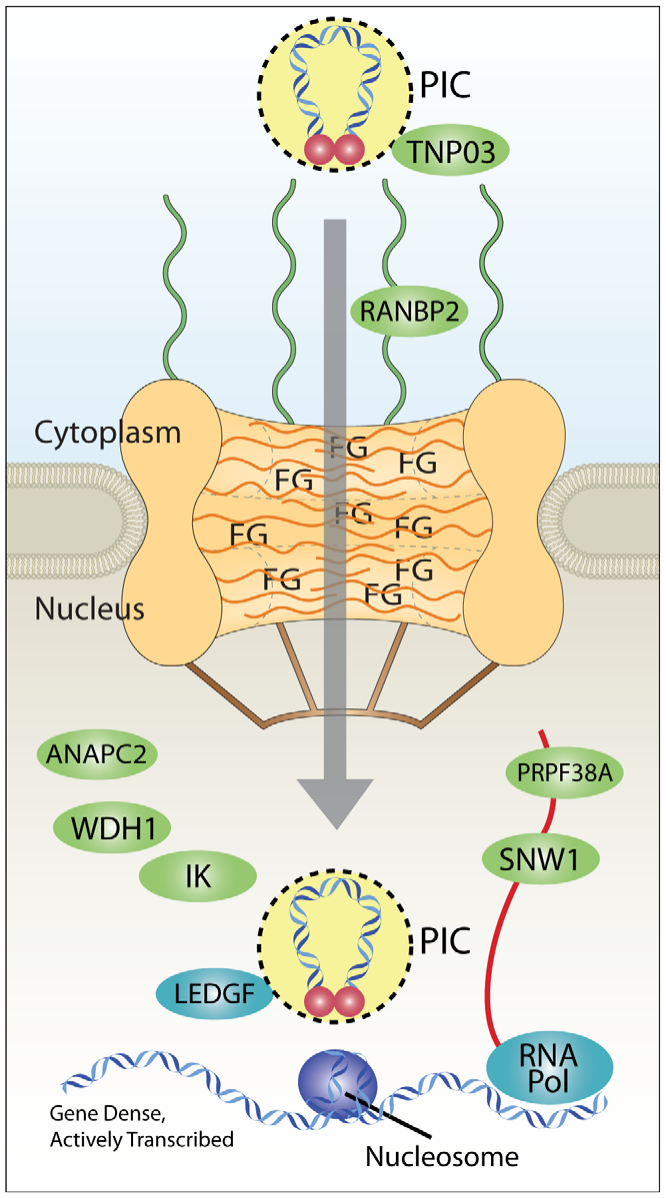
Model for coupling of nuclear import and integration targeting. Interaction with Transportin-3 and RanBP2 shuttles the PIC through the nuclear pore and toward gene dense regions favored for HIV integration. Interactions with additional factors in the nucleus (ANAPC2, WDH1, IK, PRPF38A, and SNW1) may also play a role in site selection upstream of the known integration cofactor Ledgf/p75, which targets integration to active transcription units. RNA Pol indicates RNA polymerase II, which is known to be required for transcriptional activity, and which promotes integration [13]–[15], [18]. Nucleosomes are shown because target DNA is known to be wrapped in nucleosomes during the integration step [36], [57]–[60]. PIC, preintegration complex; FG, phenylalanine-glycine repeat sequences of nuclear pore proteins.

Our studies support the hypothesis that nuclear import of HIV is linked to integration, and suggest that normal interactions with the nuclear pore help to determine integration target site distributions (Figure 9). We favor a two-step model, in which passage through the pore first places the PIC in regions of high gene density, and then Ledgf/p75 tethers the PIC for integration to provide the final distribution in active transcription units. Several studies suggest that chromosomes and genes are nonrandomly distributed in the nucleus, though the organization is not fully clarified [42]–[44]. Although the nuclear periphery is thought to be rich in heterochromatic chromosomal regions that promote gene silencing, studies in yeast and Drosophila suggested that genes can relocate to the nuclear pore upon transcriptional induction [45]–[50]. Thus passage through the pore may deliver HIV to locally concentrated active gene-dense chromatin. Alternatively, interaction with Transportin-3 and RanBP2 at the pore might engage a nuclear transport system leading to gene-dense chromatin.

Our data is consistent with the idea that correct engagement of the Transportin-3/RanBP2-dependent targeting pathway leads to efficient integration in chromosomal regions rich in genes and associated features. Failure to engage this pathway results in targeting to less gene dense regions. Two possible scenarios can be imagined for nuclear entry and integration targeting in cells depleted for pore factors Transporting-3 and RanBP2.

The first model is that in the absence of Transportin-3 or RanBP2, nuclear access of HIV is restricted to times of nuclear envelope breakdown during cell division. The shift in integration away from gene-dense regions in the TNPO3 and RANBP2 knockdowns may thus reflect changes in chromatin availability during mitosis or shortly afterwards. Consistent with this idea, the HIVmGag virus requires nuclear envelope breakdown during mitosis for infection [41], and it phenocopied HIV integration in the knockdown cells, showing reduced integration frequency in gene dense regions.

An extreme version of this model would hold that HIV integration targeting in TNPO3 and RANBP2 knockdowns might mimic MLV targeting because in both cases the virus accesses chromatin during nuclear breakdown. However, MLV strongly favors integration near transcription start sites, and this is not seen for HIV in knockdown cells (Figure 8).

Similarly, if passage through the nuclear pore delivers the HIV PIC to transcription units and gene dense regions, growth arrest of cells might increase favoring of these features, since all integrants must enter through the pore in arrested cells. Integration site distributions have been investigated in growth arrested IMR90 lung fibroblasts and macrophages [28], [51]. In IMR90 cells, arrest did result in more integration in transcription units and gene dense regions, but in macrophages the favoring is in fact weaker than that observed in many other cell types [27]. Thus it is possible that passage through the nuclear pore results in favored integration in gene dense regions, but additional assumptions are needed to explain the data from macrophages.

The second model (not exclusive of the first) holds that in cells depleted of TNPO3 and RanBP2, HIV integration complexes may pass through the pore but on a different pathway, interacting with different pore proteins. The idea that alternative pathways through the pore exist is supported by findings of Lee and colleagues, who found that the N74D substitution in HIV CA disrupted normal interactions with Transportin-3 and RanBP2 but created dependence on other pore proteins [34]. From our data, it is not possible to determine whether in cells depleted of Transportin-3 and RanBP2 HIV integration complexes pass through the pore on alternate pathways, or whether nuclear access during mitosis fully explains the data. Thus it will be important to analyze targeting when integration complexes pass through the pore on alternative pathways, as in the presence of the N74D CA substitution (Schaller et al., submitted).

## Materials and Methods

### Cell culture and viral infections

HEK 293T cells were grown in D10 media (DMEM supplemented with 10% FBS and 50 ug/µL Gentamicin). For gene knockdowns, cells were grown to confluency, trypsinized and reverse transfected (100,000 cells/well in 12 well plates, 50,000/well in 24 well plates, and 8,000/well in 96 well plates) using RNAiMax (Invitrogen, Carlsbad CA) with 25 pmol/mL siRNA. The siRNAs were purchased from Qiagen (Qiagen, Valencia, CA) and are listed in Table S2. Toxicity of siRNAs was measured 48 hr after transfection both visually and by the CellTiter-Glo Luminescent Cell Viability Assay (Promega, Madison WI; see Figure S3 for details). Transfection media was replaced after 48 hr by 500 µL of D10 plus 5 ug DEAE dextran and virus in 12 well plates. Two viral inoculums were used (0.06 µL or 1 µL concentrated virus stock corresponding to 1.32 ng or 22 ng p24 per well, values determined by titration to result in infection of 30–60% or 80–100% of cells, respectively). Virus-containing media was replaced after 10–12 hours with 1 mL D10 and incubated an additional 38 hours before harvest. Infections of LEDGF stable knockdown cell lines were performed essentially as described [28].

VSV-G pseudotyped HIV vector particles were produced in HEK 293T cells by Lipofectamine (Invitrogen, Carlsbad CA) transfection of p156RRLsin-PPTCMVGFPWPRE [52], the packaging construct pCMVdeltaR9 [53], and the vesicular stomatitis virus G-producing plasmid pMD.G. VSV-G pseudotyped MLV particles were produced in a similar manner but using the MLV vector segment (pMX-eGFP) and packaging construct pCGP (pCGP, kindly provided by Paul Bates).

Percent infection was measured using GFP fluorescence, which is not strongly affected by integration site placement in the HIV-based vectors with strong artificial promoters used here [30].

HIV infection and targeting rescue experiments were performed as described for siRNA knockdowns but with the co-transfection of siRNA-resistant or empty expression vectors (333 ng plasmid/mL). The siRNA-resistant TNPO3 allele was constructed by introducing six conservative mutations in the third position of each codon and an N-terminal 3xFLAG-tag into the TNPO3 cDNA amplified HEK-293T cells. This product was then cloned into the mammalian expression vector pLNCX (kind gift of Paul Bates), engineered to contain a WPRE.

### Gene expression by RNA and protein levels

Q-PCR (see Figure S1 for details) and immunoblotting were used to monitor the extent of siRNA knockdowns. Protein levels were measured by immunoblotting using antibodies against Transportin-3 (ab54353, Abcam Inc., Cambridge, MA) and RanBP2 (ab2938, Abcam Inc., Cambridge, MA). HRP conjugated secondary antibodies (p0260, DAKO A/S, Denmark, and ab6721-1, Abcam, Cambridge, MA) were used for detection with SuperSignal West Pico Chemiluminescent Substrate (Thermo Scientific, Pierce Protein Research Products, Rockford, IL). Beta-tubulin was used as a loading control, detected by the HRP conjugated antibody (ab21058, Abcam, Cambridge, MA).

### Integration site analysis

For integration site recovery, purified genomic DNA was digested overnight with MseI, ligated at 16°C to PCR adapters, and digested a second time with SacI. Nested PCR was then performed using primers and conditions described previously [36], [54]. Amplification products between 200–600 bp were then gel-excised, purified, and sequenced on a Genome Sequencer FLX Titanium Series (Roche 454 Sequencing) at either the University of Pennsylvania or the University of Florida. Only sequences that began within three base pairs of the LTR end and showed unique best alignments to the human genome by BLAT (hg18, version 36.1, >98% match score) were considered true integration sites. Identical integration sites identified in two or more separately amplified samples were considered to be PCR contamination and were omitted.

Comparisons to genomic features were carried out as described previously [18], [55] using a combination of conditional logit regression and Bayesian model averaging. Details of statistical methods are available in [14], [18], [55], [56]. Methods used for statistical analysis of ROC areas (Figures 3 and 8) are summarized in [56]. Gene expression analyses utilized data from 293T cells [28] with expression measured using the Affymetrix HU133 plus 2.0 gene chip array. All integration site sequences will be deposited in publicly accessible databases (NCBI) upon acceptance of this manuscript for publication.

### Entrez Gene ID numbers for genes mentioned in the text

NUP98: 4928, MAP4: 4134, IK: 3550, ANAPC2: 29882, PRPF38A: 84950, RANBP2/NUP358: 5903, SNW1: 22938, TNPO3: 23534, WDR46: 9277, WDHD1:11169, PSIP1/LEDGF/p75: 493969.

## Supporting Information

Figure S1 mRNA levels under normal and gene knockdown conditions. 293T cells were reverse transfected as described in Materials and Methods (8,000/well in 96 well plates using RNAiMax (Invitrogen, Carlsbad CA) with 25 pmol/mL siRNA, then incubated 48 hr at 37°C before harvest. RNA was purified from cells using either the RNeasy Mini Kit from Qiagen (Carlsbad, CA) or the RNAspin Mini Kit (GE Healthcare, Buckinghamshire UK) per manufacturer's instructions. RT-PCR was carried out using the High Capacity RNA to cDNA Kit (Applied Biosystems, Foster City CA) and relative RNA levels were measured by the ddCt method using Taqman Gene Expression Assays (Applied Biosystems, Foster City CA) with GUSB as the internal reference. Assays IDs were Hs00193785_m1, Hs00600887_m1, Hs00173172_m1, Hs00273527_m1, Hs00159048_m1, Hs00610583_m1, Hs01108576_m1, Hs00203499_m1, Hs00273351_m1, Hs00180522_m1 for genes measured for knockdown and product number 4333767F for the GUSB endogeneous control assay. All values were normalized the control siRNA, GL2. Data presented is representative of at least three replicate experiments.
(1.58 MB TIF)

Figure S2 HIV infection levels under normal and gene knockdown conditions. 48 hr following siRNA transfection, media was replaced with 500 µL of D10 (12 well plates) plus 5 ug DEAE dextran and virus as described in Materials and Methods (0.06 µL concentrated virus stock corresponding to 1.32 ng p24 per well, innoculum determined by titration to result in infection of 30–60% of cells). Virus-containing media was replaced after 10–12 hours with 1 mL D10 and incubated an additional 38 hours before harvest. Infection level was measured by flow cytometry as the percentage of GFP positive cells. All values normalized to Mock controls.
(0.77 MB TIF)

Figure S3 Cell viability after siRNA transfection. Toxicity of siRNAs was measured 48 hr after transfection both visually and by the CellTiter-Glo Luminescent Cell Viability Assay (Promega, Madison WI) following the manufacturer's instructions. Cells were reverse transfected in 96 well plates with the indicated siRNAs at 25 pmol/ml final concentration and incubated at 37°C. All values normalized to GL2 controls. Data shown is representative of at least two independent experiments.
(1.49 MB TIF)

Figure S4 SNW1 protein levels under normal and gene knockdown conditions. Cells were reverse transfected with SNW1 si5 or with GL2 as described, incubated 48 hr, harvested, and lysed for protein analysis. Blotting was done using rabbit polyclonal antibody from Santa Cruz Biotechnology (Santa Cruz, CA; product SC-30139 Lot B1506). Following gel transfer, PVDF membranes were incubated 2.5 hr at RT (antibodies diluted 1∶2000 in PBST, 5% milk) followed by incubation for 1 hr at RT with secondary antibody was Abcam HRP conjugated Goat anti Rabbit (goat polyclonal to Rabbit IgG; ab6721-1 lot 142201, diluted 1∶2000 in PBST, 5% milk). Knockdown of protein levels for ANAPC2 could not be confirmed by western blot (Abcam, product ab18295).
(1.12 MB TIF)

Figure S5 Effects of Transportin-3 and RanBP2 depletion on integration near multiple chromosomal features: interactive heat map. Data was analyzed and is displayed as described in Figure 2 and Text S1. To view, download and open zip file, and follow instructions in the included ReadMe.txt document.
(0.21 MB ZIP)

Figure S6 Partial rescue of HIV integration site distributions by Transportin-3 allele insensitive to TNPO3 si4. Cells were cotransfected with siRNA and either empty vector plasmid or rescue plasmid encoding siRNA-resistant alleles of Transportin-3, infected with a VSVG-pseudotyped HIV-1 vector, and harvested for integration site analysis as described. Histogram shown indicates distribution of integration sites with respect to gene density. Integration sites in each dataset were binned (along the X-axis) according to the number of genes within 1 MB interval surrounding each site (counted as shown in Figure 1B). Curves were computed from histogram plot using Gaussian kernal density estimates.
(1.15 MB TIF)

Figure S7 MLV infection and integration site distributions after siRNA treatment targeting SNW1 and ANAPC2. MLV infections were carried out using VSV-G pseudotyped, single round viral vectors in the same manner described for HIV infections (see Materials and Methods and Supplementary Figure 2). Infection level was measured by flow cytometry as the percentage of GFP positive cells. All values normalized to GL2 controls.
(1.54 MB TIF)

Figure S8 Effects of MLV-Gag swap on integration near multiple chromosomal features: interactive heat map. Data was analyzed and is displayed as described in Figure 7c and Text S1. To view, download and open zip file, and follow instructions in the included ReadMe.txt document.
(0.29 MB ZIP)

Table S1 Integration site data sets used in this study.
(0.08 MB PDF)

Table S2 DNA and RNA oligonucleotides used in this study.
(0.08 MB PDF)

Text S1 Guide to Interpreting Genomic Heat Maps Summarizing Integration Site Distributions.
(0.12 MB PDF)

Text S2 Distributions of HIV integration sites after TNPO3 knockdown and rescue with siRNA insensitive allele.
(0.58 MB PDF)

Text S3 Integration site preference under gene silencing.
(1.33 MB PDF)

## References

[1] Roe T, Reynolds TC, Yu G, Brown PO (1993). Integration of murine leukemia virus DNA depends on mitosis.. EMBO J.

[2] Lewis PF, Emerman M (1994). Passage through mitosis is required for oncoretroviruses but not for the human immunodeficiency virus.. J Virol.

[3] Bukrinsky MI, Sharova N, Dempsey MP, Stanwick TL, Bukrinskaya AG (1992). Active nuclear import of human immunodeficiency virus type 1 preintegration complexes.. Proc Natl Acad Sci U S A.

[4] von Schwedler U, Kornbluth RS, Trono D (1994). The nuclear localization signal of the matrix protein of human immunodeficiency virus type 1 allows the establishment of infection in macrophages and quiescent T lymphocytes.. Proc Natl Acad Sci U S A.

[5] Heinzinger NK, Bukrinsky MI, Haggerty SA, Ragland AM, Kewalramani V K (1994). The vpr protein of human immunodeficiency virus type 1 influences nuclear localization of viral nucleic acids in nondividing host cells.. Proc Natl Acad Sci U S A.

[6] Brass AL, Dykxhoorn DM, Benita Y, Yan N, Engelman A (2008). Identification of host proteins required for HIV infection through a functional genomic screen.. Science.

[7] Konig R, Zhou Y, Elleder D, Diamond TL, Bonamy GM (2008). Global analysis of host-pathogen interactions that regulate early-stage HIV-1 replication.. Cell.

[8] Zhou H, Xu M, Huang Q, Gates AT, Zhang XD (2008). Genome-scale RNAi screen for host factors required for HIV replication.. Cell Host Microbe.

[9] Christ F, Thys W, De Rijck J, Gijsbers R, Albanese A (2008). Transportin-SR2 imports HIV into the nucleus.. Curr Biol.

[10] Suzuki Y, Craigie R (2007). The road to chromatin - nuclear entry of retroviruses.. Nat Rev Microbiol.

[11] Ebina H, Aoki J, Hatta S, Yoshida T, Koyanagi Y (2004). Role of Nup98 in nuclear entry of human immunodeficiency virus type 1 cDNA.. Microbes Infect.

[12] Katz RA, Greger JG, Boimel P, Skalka AM (2003). Human immunodeficiency virus type 1 DNA nuclear import and integration are mitosis independent in cycling cells.. J Virol.

[13] Schroder AR, Shinn P, Chen H, Berry C, Ecker JR (2002). HIV-1 integration in the human genome favors active genes and local hotspots.. Cell.

[14] Mitchell RS, Beitzel BF, Schroder AR, Shinn P, Chen H (2004). Retroviral DNA integration: ASLV, HIV, and MLV show distinct target site preferences.. PLoS Biol.

[15] Wu X, Li Y, Crise B, Burgess SM (2003). Transcription start regions in the human genome are favored targets for MLV integration.. Science.

[16] Carteau S, Hoffmann C, Bushman FD (1998). Chromosome structure and HIV-1 cDNA integration: Centromeric alphoid repeats are a disfavored target.. J. Virol.

[17] Holman AG, Coffin JM (2005). Symmetrical base preferences surrounding HIV-1, avian sarcoma/leukosis virus, and murine leukemia virus integration sites.. Proc Natl Acad Sci U S A.

[18] Berry C, Hannenhalli S, Leipzig J, Bushman FD (2006). Selection of target sites for mobile DNA integration in the human genome.. PLoS Comput Biol.

[19] Verdin E (1991). DNase I-hypersensitive sites are associated with both long terminal repeats and with the intragenic enhancer of integrated human immunodeficiency virus type 1.. J Virol.

[20] Lewinski M, Bisgrove D, Shinn P, Chen H, Verdin E (2005). Genome-wide analysis of chromosomal features repressing HIV transcription.. J Virol.

[21] Weinberger LS, Burnett JC, Toettcher JE, Arkin AP, Schaffer DV (2005). Stochastic gene expression in a lentiviral positive-feedback loop: HIV-1 tat fluctuations drive phenotypic diversity.. Cell.

[22] Cherepanov P, Maertens G, Proost P, Devreese B, Van Beeumen J (2003). HIV-1 integrase forms stable tetramers and associates with LEDGF/p75 protein in human cells.. J Biol Chem.

[23] Emiliani S, Mousnier A, Busschots K, Maroun M, Van Maele B (2005). Integrase mutants defective for interaction with LEDGF/p75 are impaired in chromosome tethering and HIV-1 replication.. J Biol Chem.

[24] Maertens G, Cherepanov P, Pluymers W, Busschots K, De Clercq E (2003). LEDGF/p75 is essential for nuclear and chromosomal targeting of HIV-1 integrase in human cells.. J Biol Chem.

[25] Llano M, Vanegas M, Fregoso O, Saenz D, Chung S (2004). LEDGF/p75 determines cellular trafficking of diverse lentiviral but not murine oncoretroviral integrase proteins and is a component of functional lentiviral preintegration complexes.. J Virol.

[26] Shun MC, Raghavendra NK, Vandegraaff N, Daigle JE, Hughes S (2007). LEDGF/p75 functions downstream from preintegration complex formation to effect gene-specific HIV-1 integration.. Genes Dev.

[27] Marshall H, Ronen K, Berry C, Llano M, Sutherland H (2007). Role of PSIP1/LEDGF/p75 in lentiviral infectivity and integration targeting.. PLoS One.

[28] Ciuffi A, Llano M, Poeschla E, Hoffmann C, Leipzig J (2005). A role for LEDGF/p75 in targeting HIV DNA integration.. Nat Med.

[29] Silvers RM, Smith JA, Schowalter M, Litwin S, Liang Z (2010). Modification of integration site preferences of an HIV-1-based vector by expression of a novel synthetic protein.. Hum Gene Ther.

[30] Gijsbers R, Ronen K, Vets S, Malani N, De Rijck J (2010). LEDGF hybrids efficiently retarget lentiviral integration into heterochromatin.. Mol Ther.

[31] Ferris AL, Wu X, Hughes CM, Stewart C, Smith SJ (2010). Lens epithelium-derived growth factor fusion proteins redirect HIV-1 DNA integration.. Proc Natl Acad Sci U S A.

[32] Lai MC, Lin RI, Huang SY, Tsai CW, Tarn WY (2000). A human importin-beta family protein, transportin-SR2, interacts with the phosphorylated RS domain of SR proteins.. J Biol Chem.

[33] Wu J, Matunis MJ, Kraemer D, Blobel G, Coutavas E (1995). Nup358, a cytoplasmically exposed nucleoporin with peptide repeats, ran-GTP binding sites, zinc fingers, a cyclophilin A homologous domain, and a leucine-rich region.. J Biol Chem.

[34] Lee K, Ambrose Z, Martin TD, Oztop I, Mulky A (2010). Flexible use of nuclear import pathways by HIV-1.. Cell Host Microbe.

[35] Yamashita M, Perez O, Hope TJ, Emerman M (2007). Evidence for direct involvement of the capsid protein in HIV infection of nondividing cells.. PLoS Pathog.

[36] Wang GP, Ciuffi A, Leipzig J, Berry CC, Bushman FD (2007). HIV integration site selection: Analysis by massively parallel pyrosequencing reveals association with epigenetic modifications.. Genome Res.

[37] Lander E (2001). Initial sequencing and analysis of the human genome.. Nature.

[38] Venter JC (2001). The sequence of the human genome.. Science.

[39] Krishnan L, Matreyek KA, Oztop I, Lee K, Tipper CH (2010). The requirement for cellular transportin 3 (TNPO3 or TRN-SR2) during infection maps to human immunodeficiency virus type 1 capsid and not integrase.. J Virol.

[40] Lewinski MK, Yamashita M, Emerman M, Ciuffi A, Marshall H (2006). Retroviral DNA integration: Viral and cellular determinants of target-site selection.. PLoS Pathog.

[41] Yamashita M, Emerman M (2004). Capsid is a dominant determinant of retrovirus infectivity in nondividing cells.. J Virol.

[42] Xing Y, Johnson CV, Moen PT, Jr, McNeil JA, Lawrence J (1995). Nonrandom gene organization: Structural arrangements of specific pre-mRNA transcription and splicing with SC-35 domains.. J Cell Biol.

[43] Simonis M, Klous P, Splinter E, Moshkin Y, Willemsen R (2006). Nuclear organization of active and inactive chromatin domains uncovered by chromosome conformation capture-on-chip (4C).. Nat Genet.

[44] Osborne CS, Chakalova L, Brown KE, Carter D, Horton A (2004). Active genes dynamically colocalize to shared sites of ongoing transcription.. Nat Genet.

[45] Casolari JM, Brown CR, Komili S, West J, Hieronymus H (2004). Genome-wide localization of the nuclear transport machinery couples transcriptional status and nuclear organization.. Cell.

[46] Brickner JH, Walter P (2004). Gene recruitment of the activated INO1 locus to the nuclear membrane.. PLoS Biol.

[47] Cabal GG, Genovesio A, Rodriguez-Navarro S, Zimmer C, Gadal O (2006). SAGA interacting factors confine sub-diffusion of transcribed genes to the nuclear envelope.. Nature.

[48] Taddei A, Van Houwe G, Hediger F, Kalck V, Cubizolles F (2006). Nuclear pore association confers optimal expression levels for an inducible yeast gene.. Nature.

[49] Kurshakova MM, Krasnov AN, Kopytova DV, Shidlovskii YV, Nikolenko JV (2007). SAGA and a novel drosophila export complex anchor efficient transcription and mRNA export to NPC.. EMBO J.

[50] Mendjan S, Taipale M, Kind J, Holz H, Gebhardt P (2006). Nuclear pore components are involved in the transcriptional regulation of dosage compensation in drosophila.. Mol Cell.

[51] Barr SD, Ciuffi A, Leipzig J, Shinn P, Ecker JR (2006). HIV integration site selection: Targeting in macrophages and the effects of different routes of viral entry.. Mol Ther.

[52] Follenzi A, Ailes LE, Bakovic S, Gueuna M, Naldini L (2000). Gene transfer by lentiviral vectors is limited by nuclear translocation and rescued by HIV-1 pol sequences.. Nat Genetics.

[53] Naldini L, Blomer U, Gallay P, Ory D, Mulligan R (1996). In vivo gene delivery and stable transduction of nondividing cells by a lentiviral vector. Science.

[54] Wang GP, Garrigue A, Ciuffi A, Ronen K, Leipzig J (2008). DNA bar coding and pyrosequencing to analyze adverse events in therapeutic gene transfer.. Nucleic Acids Res.

[55] Brady T, Agosto LM, Malani N, Berry CC, O'Doherty U (2009). HIV integration site distributions in resting and activated CD4+ T cells infected in culture.. AIDS.

[56] Brady T, Lee YN, Ronen K, Malani N, Berry CC (2009). Integration target site selection by a resurrected human endogenous retrovirus.. Genes Dev.

[57] Pryciak PM, Varmus HE (1992). Nucleosomes, DNA-binding proteins, and DNA sequence modulate retroviral integration target site selection.. Cell.

[58] Pryciak PM, Sil A, Varmus HE (1992). Retroviral integration into minichromosomes *in vitro*.. EMBO J.

[59] Pruss D, Bushman FD, Wolffe AP (1994). Human immunodeficiency virus integrase directs integration to sites of severe DNA distortion within the nucleosome core.. Proc Natl Acad Sci U S A.

[60] Pruss D, Reeves R, Bushman FD, Wolffe AP (1994). The influence of DNA and nucleosome structure on integration events directed by HIV integrase.. J Biol Chem.

