# Supplementary figures and images for: HIV Integration Targeting: A Pathway Involving Transportin-3 and the Nuclear Pore Protein RanBP2
Source: PLoS Pathog. 2011 Mar 10;7(3):e1001313. doi: 10.1371/journal.ppat.1001313 (PMC3053352; doi:10.1371/journal.ppat.1001313)

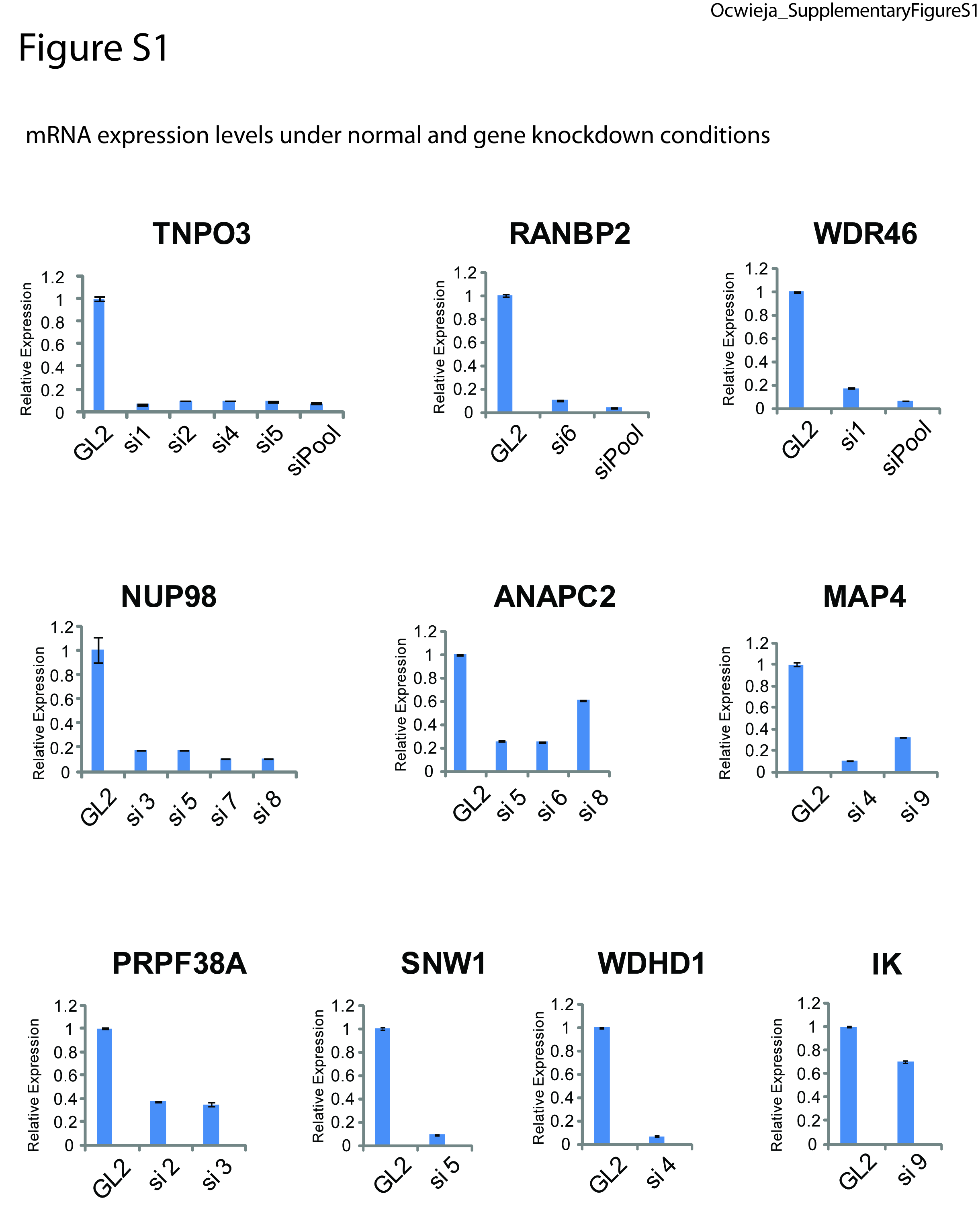

Supplement: Figure S1 — mRNA levels under normal and gene knockdown conditions. 293T cells were reverse transfected as described in Materials and Methods (8,000/well in 96 well plates using RNAiMax (Invitrogen, Carlsbad CA) with 25 pmol/mL siRNA, then incubated 48 hr at 37°C before harvest. RNA was purified from cells using either the RNeasy Mini Kit from Qiagen (Carlsbad, CA) or the RNAspin Mini Kit (GE Healthcare, Buckinghamshire UK) per manufacturer's instructions. RT-PCR was carried out using the High Capacity RNA to cDNA Kit (Applied Biosystems, Foster City CA) and relative RNA levels were measured by the ddCt method using Taqman Gene Expression Assays (Applied Biosystems, Foster City CA) with GUSB as the internal reference. Assays IDs were Hs00193785_m1, Hs00600887_m1, Hs00173172_m1, Hs00273527_m1, Hs00159048_m1, Hs00610583_m1, Hs01108576_m1, Hs00203499_m1, Hs00273351_m1, Hs00180522_m1 for genes measured for knockdown and product number 4333767F for the GUSB endogeneous control assay. All values were normalized the control siRNA, GL2. Data presented is representative of at least three replicate experiments. (1.58 MB TIF) [file ppat.1001313.s001.tif]

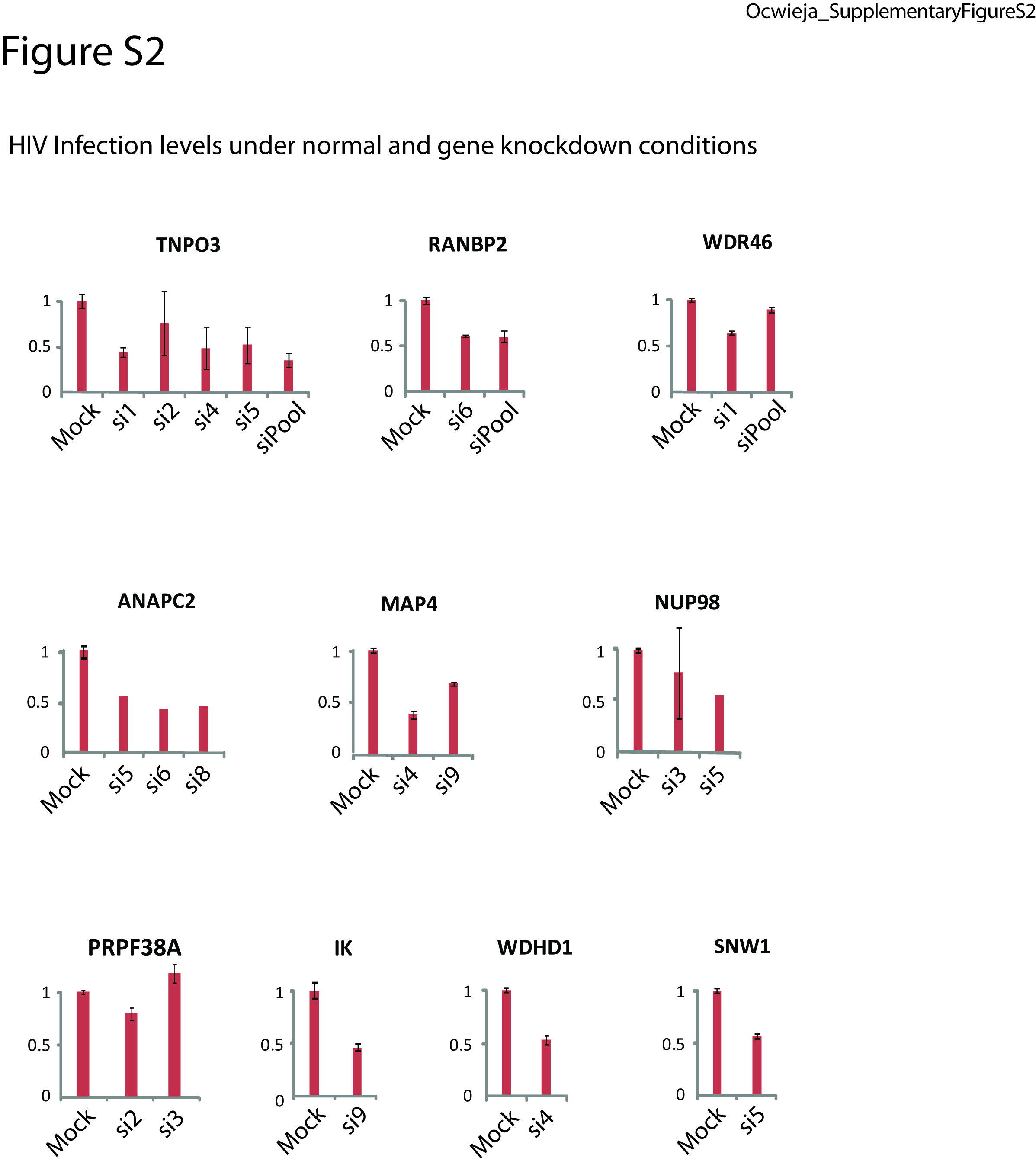

Supplement: Figure S2 — HIV infection levels under normal and gene knockdown conditions. 48 hr following siRNA transfection, media was replaced with 500 µL of D10 (12 well plates) plus 5 ug DEAE dextran and virus as described in Materials and Methods (0.06 µL concentrated virus stock corresponding to 1.32 ng p24 per well, innoculum determined by titration to result in infection of 30–60% of cells). Virus-containing media was replaced after 10–12 hours with 1 mL D10 and incubated an additional 38 hours before harvest. Infection level was measured by flow cytometry as the percentage of GFP positive cells. All values normalized to Mock controls. (0.77 MB TIF) [file ppat.1001313.s002.tif]

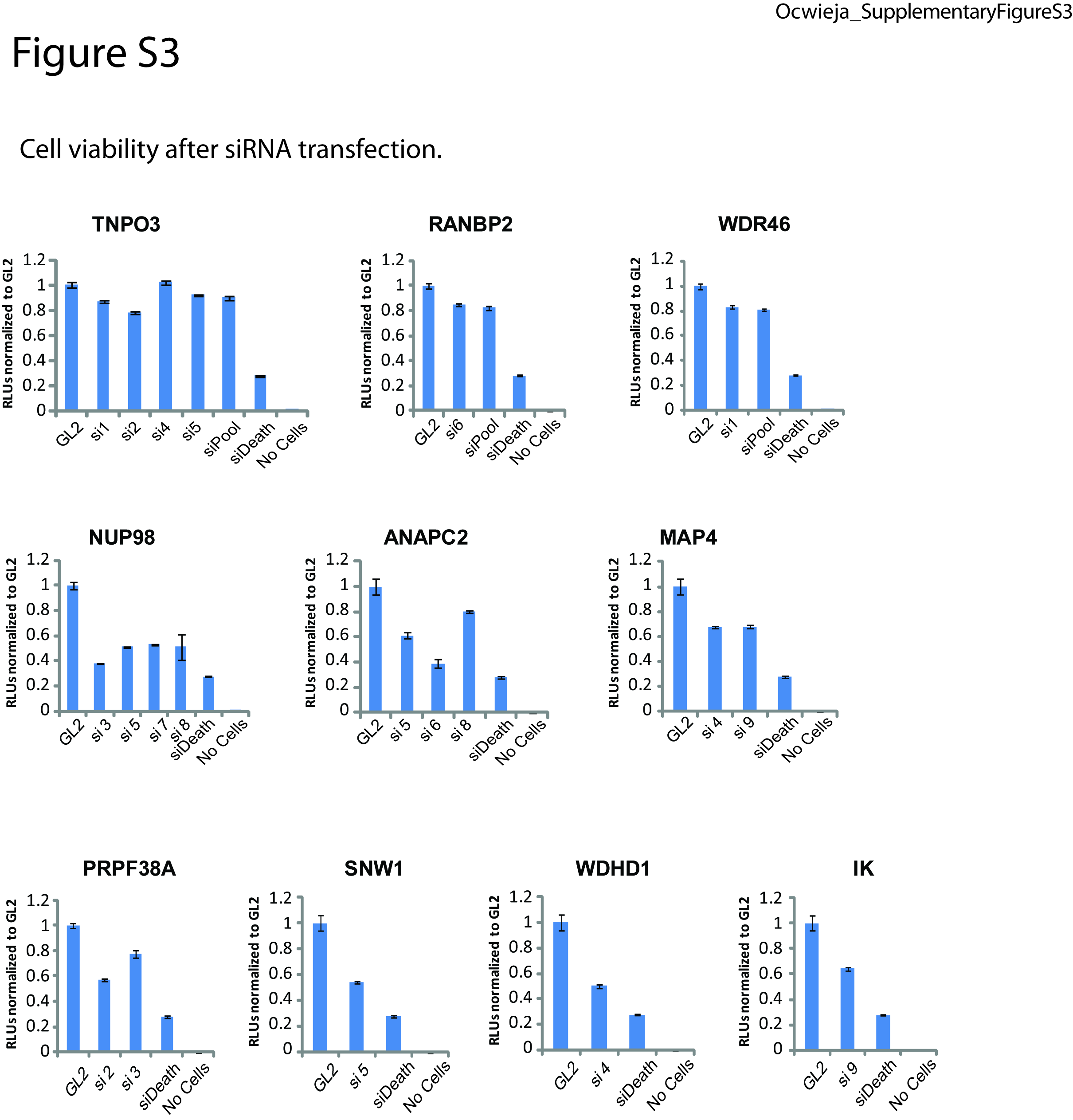

Supplement: Figure S3 — Cell viability after siRNA transfection. Toxicity of siRNAs was measured 48 hr after transfection both visually and by the CellTiter-Glo Luminescent Cell Viability Assay (Promega, Madison WI) following the manufacturer's instructions. Cells were reverse transfected in 96 well plates with the indicated siRNAs at 25 pmol/ml final concentration and incubated at 37°C. All values normalized to GL2 controls. Data shown is representative of at least two independent experiments. (1.49 MB TIF) [file ppat.1001313.s003.tif]

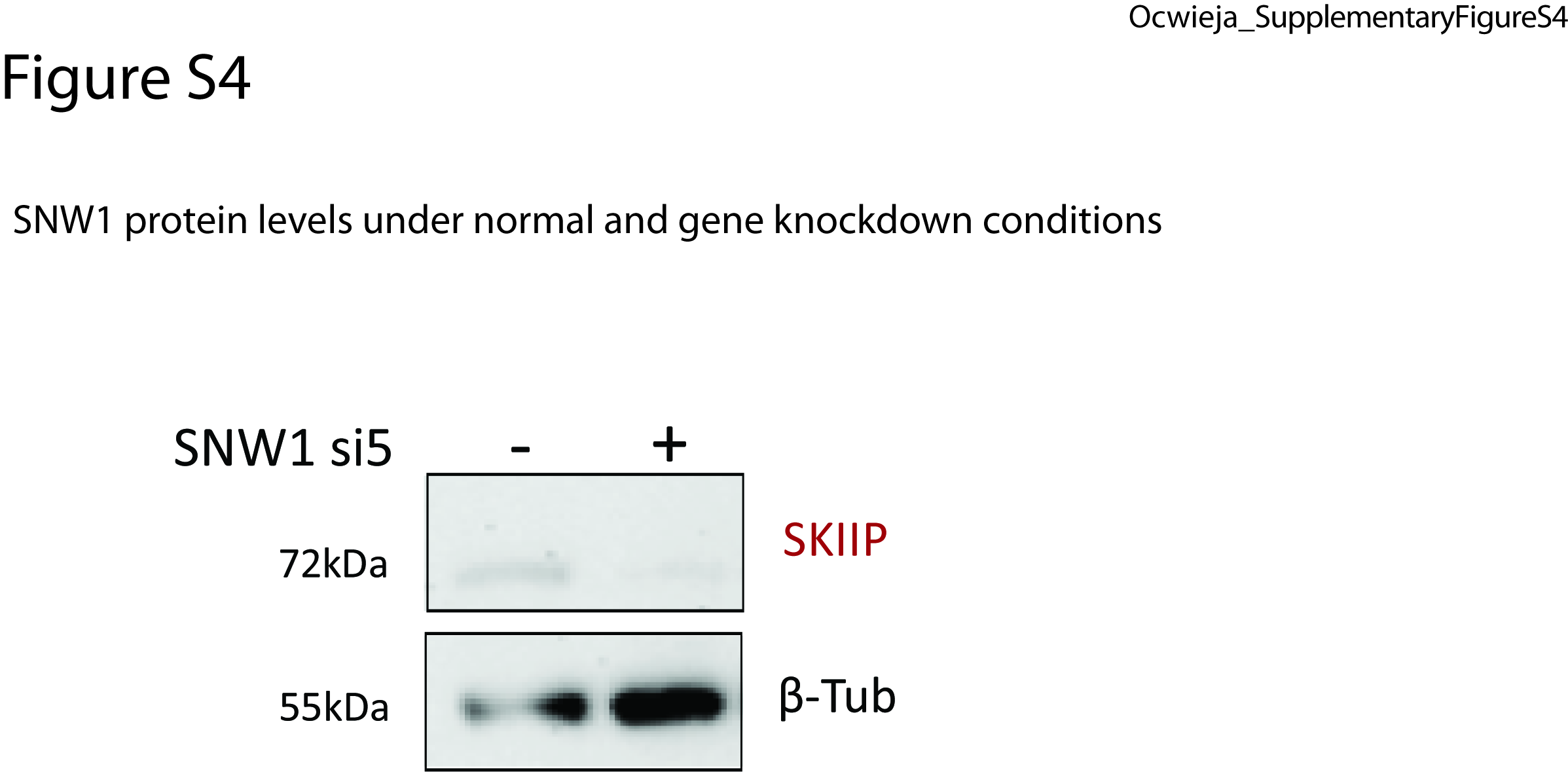

Supplement: Figure S4 — SNW1 protein levels under normal and gene knockdown conditions. Cells were reverse transfected with SNW1 si5 or with GL2 as described, incubated 48 hr, harvested, and lysed for protein analysis. Blotting was done using rabbit polyclonal antibody from Santa Cruz Biotechnology (Santa Cruz, CA; product SC-30139 Lot B1506). Following gel transfer, PVDF membranes were incubated 2.5 hr at RT (antibodies diluted 1∶2000 in PBST, 5% milk) followed by incubation for 1 hr at RT with secondary antibody was Abcam HRP conjugated Goat anti Rabbit (goat polyclonal to Rabbit IgG; ab6721-1 lot 142201, diluted 1∶2000 in PBST, 5% milk). Knockdown of protein levels for ANAPC2 could not be confirmed by western blot (Abcam, product ab18295). (1.12 MB TIF) [file ppat.1001313.s004.tif]

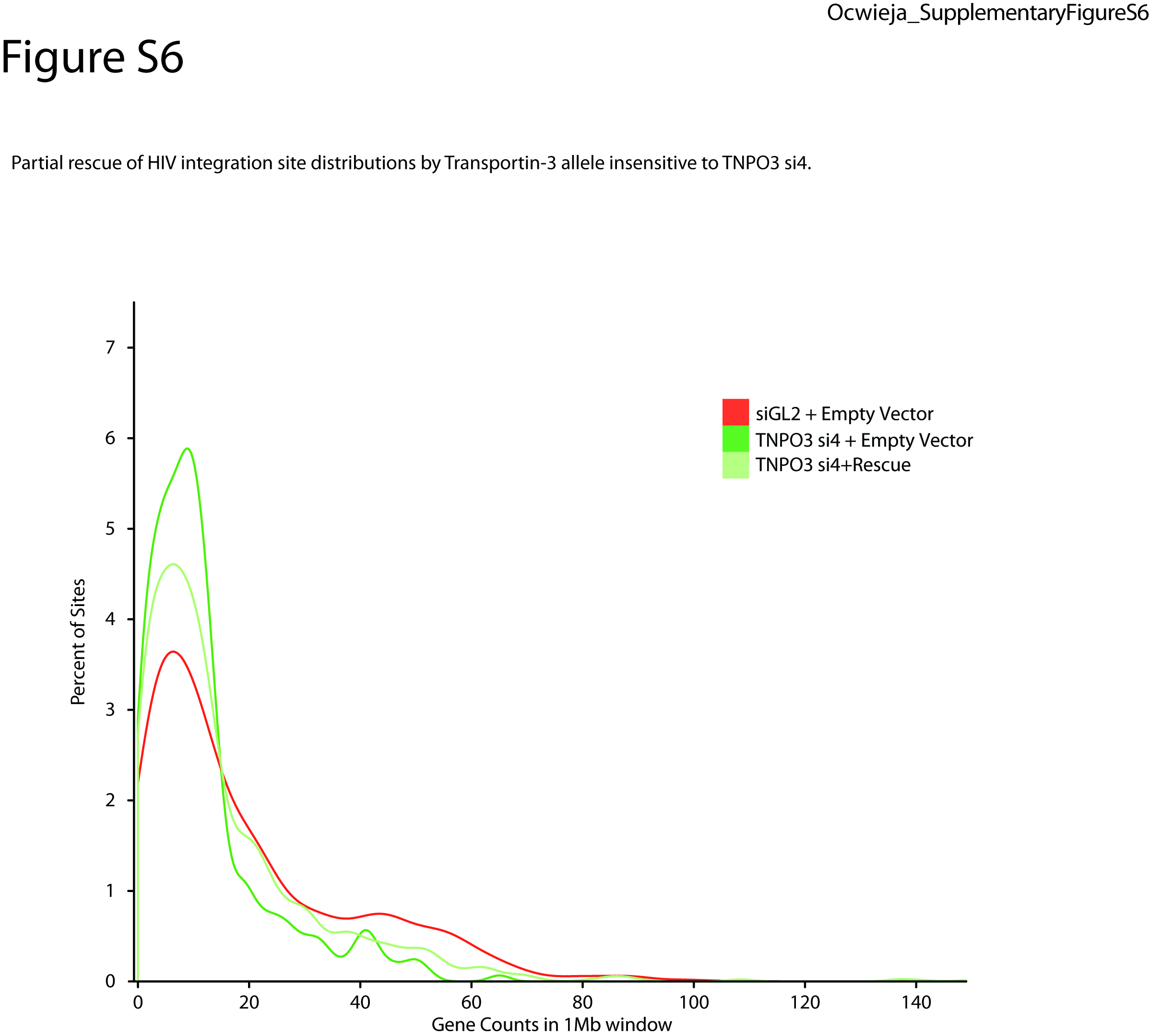

Supplement: Figure S6 — Partial rescue of HIV integration site distributions by Transportin-3 allele insensitive to TNPO3 si4. Cells were cotransfected with siRNA and either empty vector plasmid or rescue plasmid encoding siRNA-resistant alleles of Transportin-3, infected with a VSVG-pseudotyped HIV-1 vector, and harvested for integration site analysis as described. Histogram shown indicates distribution of integration sites with respect to gene density. Integration sites in each dataset were binned (along the X-axis) according to the number of genes within 1 MB interval surrounding each site (counted as shown in Figure 1B). Curves were computed from histogram plot using Gaussian kernal density estimates. (1.15 MB TIF) [file ppat.1001313.s006.tif]

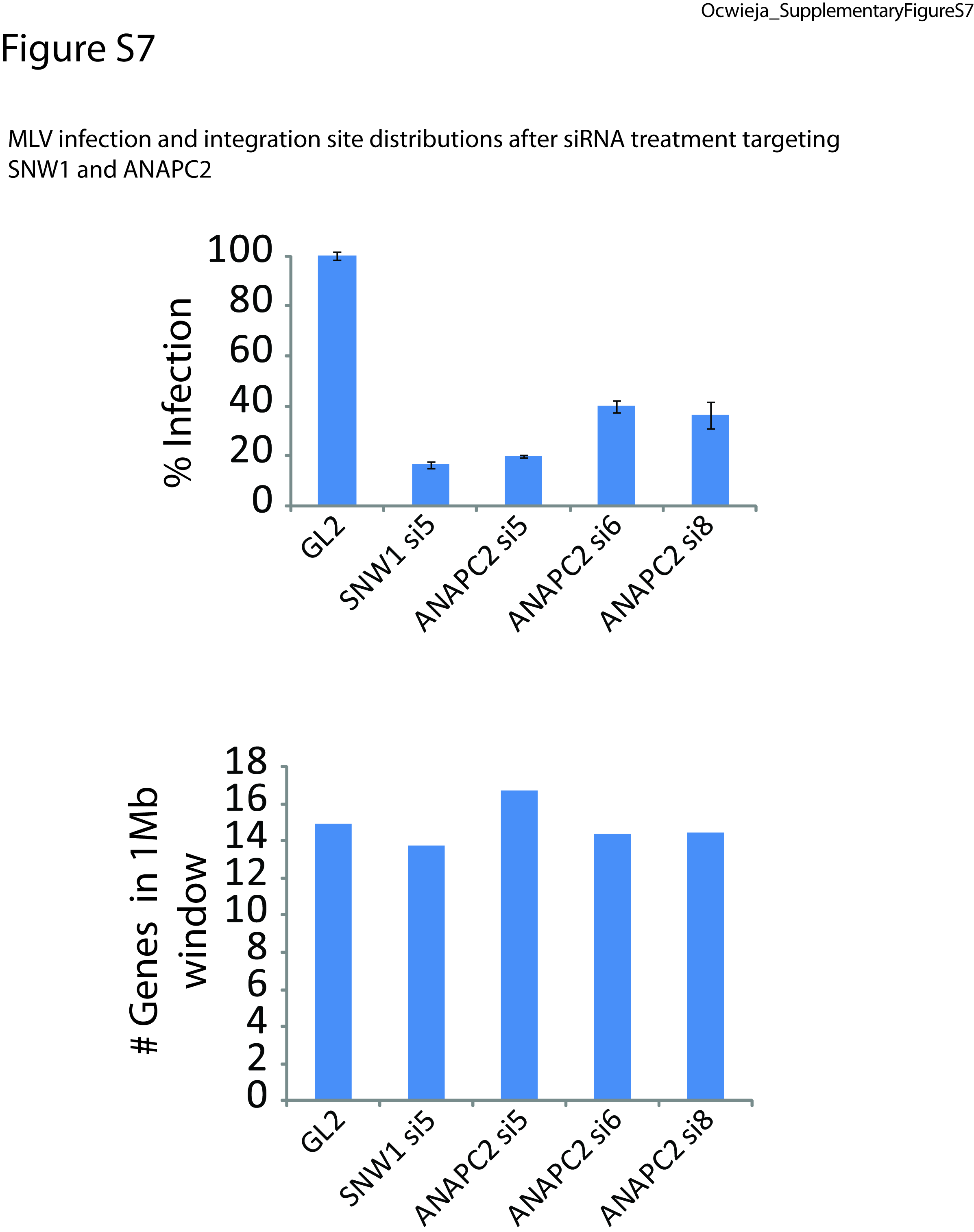

Supplement: Figure S7 — MLV infection and integration site distributions after siRNA treatment targeting SNW1 and ANAPC2. MLV infections were carried out using VSV-G pseudotyped, single round viral vectors in the same manner described for HIV infections (see Materials and Methods and Supplementary Figure 2). Infection level was measured by flow cytometry as the percentage of GFP positive cells. All values normalized to GL2 controls. (1.54 MB TIF) [file ppat.1001313.s007.tif]
