# Supplementary material for: HIV Integration Targeting: A Pathway Involving Transportin-3 and the Nuclear Pore Protein RanBP2
Source: PLoS Pathog. 2011 Mar 10;7(3):e1001313. doi: 10.1371/journal.ppat.1001313 (PMC3053352; doi:10.1371/journal.ppat.1001313)
Supplement: Table S1 — Integration site data sets used in this study. (0.08 MB PDF) [file ppat.1001313.s009.pdf]

Supplementary Table S1. Integration site data sets used in this study

siRNA Target	siRNA Number	Virus*	Target Cell Type	Number of Unique integration sites	References	Figures
Mock		HIV/high MOI	293T	270	this work	1d, 2, 5, 6
Mock		HIV	293T	43	this work	1d, 2, 5
Luciferase (GL2)		HIV	293T	4296	this work	1c, 1d, 2, 5, 6, 8
ANAPC2	5	HIV/high MOI	293T	295	this work	5, 6
ANAPC2	5	HIV	293T	316	this work	5, 6
ANAPC2	6	HIV/high MOI	293T	120	this work	5
ANAPC2	8	HIV/high MOI	293T	197	this work	5, 6
ANAPC2	8	HIV	293T	107	this work	5
IK	9	HIV/high MOI	293T	641	this work	5, 6
IK	9	HIV	293T	376	this work	5, 6
LEDGF/PSIP1/p75	shRNA	HIV/high MOI	293T	462	this work, cells described in Ciuffi et al., 2005	5, 6
MAP4	4	HIV/high MOI	293T	444	this work	5, 6
MAP4	4	HIV	293T	38	this work	5
MAP4	9	HIV/high MOI	293T	419	this work	5, 6
MAP4	9	HIV	293T	641	this work	5, 6
MAP4	3	HIV/high MOI	293T	3696	this work	5, 6
MAP4	4	HIV/high MOI	293T	3394	this work	5, 6
NUP98	3	HIV/high MOI	293T	128	this work	5
NUP98	3	HIV	293T	94	this work	5
NUP98	5	HIV/high MOI	293T	171	this work	5
NUP98	5	HIV	293T	493	this work	5, 6
NUP98	7	HIV/high MOI	293T	125	this work	5
NUP98	7	HIV	293T	75	this work	5
NUP98	8	HIV/high MOI	293T	84	this work	5
NUP98	8	HIV	293T	88	this work	5
RANBP2	6	HIV/high MOI	293T	458	this work	5, 6
RANBP2	6	HIV	293T	344	this work	1c, 1d, 2, 5, 6, 8
RANBP2	pool (2.4.5.6)	HIV/high MOI	293T	470	this work	5, 6
RANBP2	pool (2.4.5.6)	HIV	293T	363	this work	1d, 2, 5, 6
SNW1	5	HIV/high MOI	293T	153	this work	5
SNW1	5	HIV	293T	527	this work	5, 6
TNPO3	1	HIV/high MOI	293T	75	this work	1d, 5
TNPO3	1	HIV	293T	51	this work	1d, 2, 5
TNPO3	2	HIV/high MOI	293T	165	this work	5
TNPO3	2	HIV	293T	510	this work	2, 5,6
TNPO3	4	HIV/high MOI	293T	171	this work	5
TNPO3	4	HIV	293T	260	this work	1c, 1d, 2, 5, 6, 8
TNPO3	5	HIV/high MOI	293T	600	this work	5, 6
TNPO3	5	HIV	293T	297	this work	1d, 2, 5, 6
TNPO3	pool (1,2,4,5)	HIV/high MOI	293T	186	this work	5
TNPO3	pool (1,2,4,5)	HIV	293T	216	this work	1d, 2, 5, 6
WDHD1	4	HIV/high MOI	293T	240	this work	5, 6
WDHD1	4	HIV	293T	7	this work	5
WDR46	1	HIV/high MOI	293T	178	this work	5
WDR46	1	HIV	293T	220	this work	5, 6
WDR46	pool (1,2,C6Orf11_2, C6Orf11_4)	HIV/high MOI	293T	260	this work	5, 6
WDR46	pool (1,2,C6Orf11_2, C6Orf11_4)	HIV	293T	292	this work	5, 6
PRPF38A	2	HIV	293T	1577	this work	5, 6
PRPF38A	3	HIV	293T	705	this work	5, 6
GL2+Empty Vector		HIV	293T	1226	this work	3, S5
TNPO3+Rescue	4	HIV	293T	1492	this work	3, S5
TNPO3+Empty Vector	4	HIV	293T	314	this work	3, S5
GL2+Empty Vector		MLV	293T	616	this work	4, 8
TNPO3+Rescue	4	MLV	293T	354	this work	4
TNPO3+Empty Vector	4	MLV	293T	376	this work	4, 8
SNW1+Empty Vector	5	MLV	293T	90	this work	S6
ANAPC2 +Empty Vector	5	MLV	293T	79	this work	S6
ANAPC2 +Empty Vector	6	MLV	293T	32	this work	S6
ANAPC2 +Empty Vector	8	MLV	293T	251	this work	S6
None		HIVPuro	HeLa	485	Lewinski et al. PLOS Pathog 2006	7
None		HIVmGag	HeLa	452	Lewinski et al. PLOS Pathog 2006	7

* Infections were performed using enough HIV/MLV vector stock to infect 30-60% of untreated cells except where marked as “highMOI” where 90-100% of untreated cells were infected
