# Supplementary material for: HIV Integration Targeting: A Pathway Involving Transportin-3 and the Nuclear Pore Protein RanBP2
Source: PLoS Pathog. 2011 Mar 10;7(3):e1001313. doi: 10.1371/journal.ppat.1001313 (PMC3053352; doi:10.1371/journal.ppat.1001313)
Supplement: Table S2 — DNA and RNA oligonucleotides used in this study. (0.08 MB PDF) [file ppat.1001313.s010.pdf]

Supplementary Table S2 - DNA and RNA oligonucleotides used in this study

Primer Sequences

Oligo	Sequence	Description
**Mse linker+**	GTAATACGACTCACTATAGGGCTCCGCTTAAGGGAC	Linker oligo plus strand
**Mse linker-**	[Phosp]TAGTCCCTTAAGCGGAG-[AmC7-Q]	Linker oligo minus strand, 5'phosphate and 3' amide C7 modification
**MseL1**	GTAATACGACTCACTATAGGGC	Linker primer for first round PCR
**MKL-3**	CTTAAGCCTCAATAAAGCTTGCCTTGAG	HIV LTR primer for first round PCR
**GW-3**	GCCTCCCTCGCGCCATCAGAGGGCTCCGCTTAAGGGAC	Linker primer for second round PCR
**Barcoded B-HIV**	GCCTTGCCAGCCCGCTCAG**XXXXXXXX**AGACCCTTTTAGTCAGTGTGGAAAATC	HIV LTR primer for second round PCR, XXXXXXXX represents 8bp barcode for 454 sequencing
**KR-5**	CCTCTTGCAGTTGCATCCGAC	MLV LTR primer for first round PCR
**Barcoded B-MLV**	GCCTTGCCAGCCCGCTCAG**XXXXXXXX**TGATTGACTACCCGTCAGCGG	MLV LTR primer for second round PCR, XXXXXXXX represents 8bp barcode for 454 sequencing
**kr237**	gtcaccttgctgcggagcCAgGTcGTgATtCCcATtTTgCagtgggccattgcctctac	TNPO3 quikchange fwd siRNA_4
**kr238**	GTAGAGGCAATGGCCCACTGCAAAATGGGAATCACGACCTGGCTCCGCAGCAAGGTGAC	TNPO3 quikchange rev siRNA_4

siRNA Information and Sequences

Target	siRNA Number	siRNA sequence	Product ID	Product Name
**AllStarDeath**		Proprietary	1027298	AllStars Hs Cell Death Control siRNA
**ANAPC**	1	GAGAGTCTATATGCAGAGTAA	SI00101829	Hs_ANAPC2_1
	5	AAGGTTCTTCTACCGCATCTA	SI02652951	Hs_ANAPC2_5
	6	CTCACTGGATCGTATCTACAA	SI02653203	Hs_ANAPC2_6
	8	ACAGGCGGTGATCTTGCTGTA	SI04439456	Hs_ANAPC2_8
**AQR**	2	CAGGATGGATTTAGCCGACTA	SI00299530	Hs_AQR_2
	5	CAAGGCCTATTTAATGTCAAT	SI04168675	Hs_AQR_5
	6	CCCTACGGTGTCCCAAATCAA	SI04217899	Hs_AQR_6
	7	TTGCTGGTATCTCGTCATGAA	SI04366859	Hs_AQR_7
**GL2 (irrel siRNA)**		AACGTACGCGGAATACTTCGA	1022070	Luciferase GL2 siRNA
**IK**	1	AACCCGTAACAAGAAGCTTAA	SI00084336	Hs_IK_1
	3	CAGCGAGTATATGAACAACAA	SI00084350	Hs_IK_3
	4	CAGGCGCTTCAAGGAAACCAA	SI00084357	Hs_IK_4
	9	AATGACATTGTCATTAGCAAG	SI00300531	Hs_IK_9
**MAP4**	3	CCCAGATTTCCAAATCATTAA	SI00627809	Hs_MAP4_3
	4	AAGATTCTTATTGCACTTGTA	SI00627816	Hs_MAP4_4
	8	CAAGGTGGGATCCCTCGATAA	SI04315731	Hs_MAP4_8
	9	AACCCGAAAGCCTGAATCTAA	SI04337053	Hs_MAP4_9
**NUP98**	3	CTGGAGTTAGCACTAACATAA	SI00115332	Hs_NUP98_3
	5	CAGTGTATTACTGCTATGAAA	SI02635115	Hs_NUP98_5
	7	AACCCTATTGCCAAACCTATT	SI03028578	Hs_NUP98_7
	8	CTCACTAAGGTTGGTTACTAT	SI03088491	Hs_NUP98_8
**PRPF38A**	1	TCCGACGGAAATAGAATTGAA	SI00395801	Hs_FLJ14936_1
	2	CAGGCCTAGTCAGTAGCAGTA	SI04159225	Hs_PRPF38A_2
	3	TCCACGGACATCGATAATCAA	SI04186567	Hs_PRPF38A_3
	4	CGGCTTCAAGATGGTCGCCTA	SI04246634	Hs_PRPF38A_4
**RanBP**	2	CAGGTGGGAACAGGATTTAAA	SI00698250	Hs_RANBP2_2
	4	CAGCTTGGTTCTGATGATAGA	SI00698264	Hs_RANBP2_4
	5	AGCGCGGTGTTGGAGATATAA	SI03043901	Hs_RANBP2_5
	6	TCGTTTCGATGTAGAATCCAA	SI03117310	Hs_RANBP2_6
**SNW1**	2	TCGGATGGTAGAAATGCAGAA	SI00098609	Hs_SKIIP_2
	3	CACAGGTCTCTCCAAAGTGAA	SI00098616	Hs_SKIIP_3
	5	AACGTCTGGCTGCTGATGGAA	SI00301812	Hs_SKIIP_5

	7	CCCGATGAAGAAGCTATTAAA	SI02655478	Hs_SKIIP_7
**TNPO3**	1	ACCGAATGTCTTAGTGAACTA	SI00100513	Hs_TNPO3_1
	2	CTGGAGATCCTTACAGTGTTA	SI00100520	Hs_TNPO3_2
	4	CAAGTGGTCATCCCTATCTTA	SI00100534	Hs_TNPO3_4
	5	CTGGGAGATCATGCAGGTTGA	SI02639721	Hs_TNPO3_5
**WDHD1**	1	CTGCACTTAGTCGAACTACAA	SI00094472	Hs_WDHD1_1
	2	AAGGACGAGTAAATCCCTTTA	SI00094479	Hs_WDHD1_2
	4	TGCTGTGAATTTAGCCATTAA	SI00094493	Hs_WDHD1_4
	5	CAGGTAGTATTAATGGTCTAA	SI02638503	Hs_WDHD1_5
**WDR46**	1	CAGGGCCTCCTCGTAACAAGA	SI03071383	Hs_WDR46_1
	2	CAGTTTGGACCCTACAGACTA	SI03073609	Hs_WDR46_2
	11-2	TGCCCTTGATTGGGTAACAAA	SI00077455	Hs_C6orf11_2
	11-4	AAAGAAGTCTCGGATCTCTAA	SI00077469	Hs_C6orf11_4
