# Supplementary material for: HIV Integration Targeting: A Pathway Involving Transportin-3 and the Nuclear Pore Protein RanBP2
Source: PLoS Pathog. 2011 Mar 10;7(3):e1001313. doi: 10.1371/journal.ppat.1001313 (PMC3053352; doi:10.1371/journal.ppat.1001313)
Supplement: Text S1 — Guide to Interpreting Genomic Heat Maps Summarizing Integration Site Distributions. (0.12 MB PDF) [file ppat.1001313.s011.pdf]

Guide to Interpreting Genomic Heat Maps Summarizing Integration Site Distributions

General Description

We use heat maps to summarize the relationships of proviral distributions to genomic features. These maps were introduced in [1], which presents more background and examples of their uses. The goal of this Guide is to help new users interpret comparisons summarized in the genomic features heat maps.

The heat maps summarize information on integration site data sets in columns and different genomic features in rows. Colored tiles indicate the intensity and direction of any departures from the distributions of random controls for each genomic feature in each integration site data set.

This report covers heatmaps based on annotated genomic features. Other heat maps described elsewhere summarize integration intensity relative to epigenetic marks and bound proteins, measured by CHIP-seq.

Heat maps can be interactive, so that clicking on the map allows statistical comparisons to specific genomic features, to specific integration site data sets or to matched random controls. These steps are explained below. For publication, one particular statistical comparison is chosen and presented as an image file.

Abbreviations:
MRC: Matched Random Control
ROC: Receiver Operating Characteristic
TSS: Transcription Start Site

Generation of Tile Colors

Tile color indicates whether a chosen genomic feature is favored or disfavored by an integrating element, typically a retroviral vector, under the conditions studied. We determine this bias by asking how frequently that feature coincides with an integration site as compared to random sites in the genome using a *receiver operating characteristic (ROC) curve area*.

> Generation of Matched Random Control sites: The calculation of ROC area is based on comparisons between true integration sites and computationally selected random sites. Because there is a bias in recovering integration sites when using a restriction enzyme based method, k different matched random control (MRC) sites are selected for each individual integration site. Usually k will be at least 3 MRCs per integration site. Each MRC site lies the same distance from a restriction enzyme recognition sequence as the corresponding integration site but is otherwise randomly distributed in the human genome (described in [1, 2]). For example, if the enzyme MseI is used to recover integration sites, and integration site *A* lies 120 bases from the nearest MseI site, all k MRCs selected for site *A* will also lie 120 bases from the nearest MseI site.

Calculation of ROC area for genomic feature "J": The coincidence of genomic feature "J" with each integration site and matched random control site is measured (described for each feature below). Each integration site is then compared in a pair-wise fashion to its MRC sites, and a number is assigned indicating the relative rank of the integration site:

1 if the measurement of J is higher at the integration site than at the MRC site,

0 if the measurement of J is lower at the integration site than at the MRC site,

0.5 if the measurement of J is equal for the two sites.

All rank values thus calculated for a dataset of integration sites (all k rank values for all integration sites) are averaged to obtain the overall ROC area for the feature measured. An ROC area between 0 and 0.5 indicates the genomic feature occurs less frequently at/near integration sites than at/near random sites in the genome and is therefore disfavored. An ROC area between 0.5 and 1 indicates the genomic feature is enriched at integration sites. An ROC area of exactly 0.5 indicates that integration sites in the dataset are neither enriched nor depleted with respect to the feature of interest. The ROC area is converted to a color tile according to the colorimetric scale at the bottom of the heat map. Positive associations (enrichment compared with random) are shown as increasing shades of red, negative associations (depletion compared with random) as increasing shades of blue, and no difference from random as white. Each tile represents a comparison to the randomly sampled controls for one genomic feature (row) in one experimental dataset (column).

Note that we do *not* present the magnitude of effect in terms of the original units of measurement. We simply ask whether the average integration site has a higher rank for a given type of feature than its k matched random control sites. The color indicates the average quantile of each integration site relative to its random controls. This removes skewing effects contributed by non-normal distributions of the data and also reduces the effect of a few data points with extreme values for a feature.

Statistics

Statistical tests to determine whether the ROC areas calculated are significantly different from one another or from 0.5 (matched random controls). These are described for each genomic feature below and in [1] (Supplemental Material 3). All the tests rely on the variance-covariance matrix of the relative ranks of the integration sites to construct Wald-type test statistics.

For comparisons between integration site sets for specific genomic features, the Wald statistics are calculated and referred to the Chi Square distribution to obtain p values as described in [3] (Supplemental Material 3).

*=$p<0.05$, **=$p<0.01$, ***=$p<0.001$

The interactive heat maps allow comparison of integration site data sets to each other or genomic features to each other by clicking on appropriate column or row headings. If columns or rows are compared to a control, dashes overlay the control tiles. All tiles can be compared to matched random controls by clicking on the text to the upper right.

Columns: Experimental Sets
Each column of the heat map is a collection of colored tiles representing the preference of the vector for several genomic features under the condition tested (describe below). The vector/experimental condition is listed at the top of the column.

Rows: Genomic Features

<u>Gene Boundaries</u>
Analysis of integration sites with respect to the transcription start and stop sites of genes as defined by indicated databases.

1. <u>In Gene, Unigene:</u> This tile indicates how frequently integration sites occur within genes in the Unigene database. The color of the tile can be interpreted as follows: Red indicates that the average integration site in the experimental set is more often found within a gene than matched random sites. Blue indicates that the average integration site is less often located within a gene than matched random sites. If the tile is white, the integration sites are no more or less likely to fall within a gene than matched random sites.

    <u>ROC area calculation:</u> For each integration site-MRC site comparison, the integration site is scored:

    1 if the integration site lies in a gene and the MRC site is outside a gene,
    0 if the MRC site is in a gene and the integration site is not, and
    0.5 if both sites fall within a gene or outside a gene.

    ROC area is the average score across all comparisons (three per integration site) for all integration sites in the dataset.

    <u>Statistical Test for difference from Random (ROC area = 0.5)</u>: A one degree of freedom Wald test based on the variance mentioned above.

    <u>Significance:</u> HIV-1 has a known preference for integration within active transcription units [4], so a set of integration sites of HIV-1 in wild type human cells will yield a red tile in this row.

2. <u>In Gene, Refseq:</u> Calculations and tile interpretations are identical to those for "In Gene, Unigene" with the exception that genes are defined by the Refseq database.

3. General Width: Indicates the relative width of the gene or intergenic space occupied by integration sites in the experimental set. The color of the tile can be interpreted as follows: Red indicates that the average space (gene width or intergenic width) occupied by integration sites in the set is larger than the average space occupied by matched random sites in the genome. Blue indicates that this average space is smaller for integration sites than for matched random sites. White indicates that this average space is the same for integration sites as it is for a collection of random sites in the genome.

   ROC area Calculation: For an integration site that falls within a gene (Refseq), the width of the gene is measured in base pairs. Comparisons are made only to those of the three MRC sites that are also in genes. For an integration site outside a gene, the width of the interval between the nearest genes on either side of the site is measured. Comparisons are made to the MRC sites outside genes. For each comparison the integration site is scored:

   1 if the gene or intergenic interval within which an integration site lies is larger than that of the compared MRC site
   0 if the interval is smaller for the integration site, and
   0.5 if the intervals for the integration site and MRC site are matched in size.

   The ROC area is the average score of all integration sites in the dataset.

   Statistical Test for difference from Random Controls (ROC area = 0.5): A one degree of freedom Wald test based on the variance mentioned above

   Significance: The gene width values provide one measure of gene density, since gene dense regions are comprised of relatively short genes and intergenic regions. This value also correlates negatively with gene expression. Highly expressed genes tend to be shorter while less expressed genes are longer. Similarly, actively transcribed genes tend to cluster such that the intergenic width is shorter between expressed genes, longer between less expressed genes. HIV prefers to integrate in areas of the genome enriched for actively transcribed genes [4], therefore this tile will be blue for HIV-1 in wild type cells.

4. Gene Width: Displays the relative width of the gene occupied by integration sites in the experimental set. Red indicates that when integration occurs in genes, there is a bias for larger genes. Blue, that integration is favored in shorter genes. The tile is white if integration shows no bias for either short or long genes. Calculations and interpretations are similar to those for “General width”; however, for this tile, only those integration site-MRC site pairs in which both sites fall within genes are considered.

5. <u>Distance to Start</u>: Indicates whether integration is preferred near transcription start sites (TSS). A red tile means that integration sites in the experimental set are farther from TSSs than matched random sites in the genome and that gene start sites are disfavored for integration. Blue indicates that integration sites lie closer to gene starts than do random sites (gene start sites are favored). If the tile is white, TSSs are neither favored nor disfavored for integration. Note that in contrast to most features in the heat map, gene start sites are *favored* when this tile is *blue*, not red, indicating the shorter distance to start sites.

   <u>ROC area Calculation</u>: For sites that fall within genes, we measure the distance in base pairs to the TSS of that gene. For sites outside of genes, we measure the distance to the nearest transcription start site (according to RefSeq). An integration site is scored:

   1 if the distance to the nearest TSS is larger for the integration site than for the compared MRC site
   0 if the distance to the nearest TSS is smaller for the integration site
   0.5 if the integration site and MRC site are equidistant from the nearest TSS.

   The ROC area is the average score of all integration sites in the dataset.

   <u>Statistical Test for difference from Random Controls (ROC area = 0.5)</u>: A one degree of freedom Wald test based on the variance mentioned above

   <u>Example/Significance:</u> MLV and other gammaretroviruses show a strong preference for integration within gene promoters [5] and therefore, this tile would appear blue for an MLV infection of wild type cells. This makes such viruses relatively dangerous for use as gene therapy vectors, as integration in promoters may alter the control of important host genes [6-10]. By contrast, lentiviruses show little preference for gene starts and this tile appears white for HIV-1 infection of wild type cells [2, 4]. This measurement is also influenced by the preference of the vector studied for integration within genes as well as width of gene or intergenic space occupied.

6. <u>Distance to Boundary</u>: Indicates whether integration is preferred near the boundaries (transcription start or end) of genes. As with “Distance to Start” blue indicates that gene boundaries are favored for integration. Red indicates that integration disfavors gene boundaries and white indicates that the virus has no preference for gene boundaries under the conditions studied.

   <u>ROC area Calculation</u>: Calculations are similar to those for “Distance to Start” except that for all sites, the distance in base pairs to the nearest gene boundary (start or end) is considered.

Statistical Test for difference from Random Controls (ROC area = 0.5): A one degree of freedom Wald test based on the variance mentioned above

Significance: This measure is influenced by the preference of the vector studied for integration within genes, the width of gene or intergenic space occupied and the preference for transcription start sites (described above).

7. < 50 kb from Oncogene: Indicates how frequently integration sites occur within 50 kb of an oncogene (UCSC hg18 goldenpath database for genes, compared to the allOnco cancer-related gene list at http://microb230.med.upenn.edu/protocols/cancergenes.html). Red indicates that integration sites occur more frequently than random sites near oncogenes. Blue indicates that integration near oncogenes is disfavored. If the tile is white, the integration sites are no more or less likely to fall near an oncogene than matched random sites.

   ROC area calculation: An integration site is scored:

   1 if the integration site falls < 50kb from an oncogene and the compared MRC site is ≥50 kb from an oncogene,
   0 if the MRC site is <50 kb from an oncogene and integration site is not, and
   0.5 if both sites lie within or outside 50 kb of an oncogene.

   ROC area is the average score for all integration sites in the dataset.

   Statistical Test for difference from Random Controls (ROC area = 0.5): A one degree of freedom Wald test based on the variance mentioned above

   Significance: Proximity of integration sites to oncogenes is important to consider when choosing vectors to use in gene therapy applications.

DNase Sites

Analysis of integration sites with respect to the location of DNase hypersensitive sites in the genome. We ask whether integration sites fall more or less frequently within the indicated distance of a DNase hypersensitive site (<1Mb, <100kb and <10kb) than would be expected for a random distribution (location of DNase sites from UCSC hg18 goldenpath database). Red indicates a preference for integration near a DNase hypersensitive site. Blue indicates that integration is disfavored near DNase hypersensitive sites. If the tile is white, the integration sites are no more or less likely to fall within the indicated distance of a DNase hypersensitive site than matched random sites.

ROC area Calculation (eg <1Mb): An integration site is scored:

1 if the integration site falls within 1 Mb of a DNase hypersensitive site and the compared MRC site does not,
0 if the MRC site is <1 Mb from a DNase site and the integration site is not,
0.5 if both sites lie within or outside 1 Mb of a DNase hypersensitive site.

ROC area is the average score for all integration sites in the dataset.

Statistical Test for difference from Random Controls (ROC area = 0.5): A one degree of freedom Wald test based on the variance mentioned above

Significance: DNase hypersensitive sites are exposed in more active chromatin and are protected in heterochromatin. Constitutively accessible DNase hypersensitive sites are surrogate markers for open active areas of chromatin. HIV-1 prefers to integrate within actively transcribed regions, and therefore in regions rich in DNase sites. This preference appears most strongly at larger window sizes and serves as a marker for gene dense regions. HIV disfavors integration near DNAseI sites at very short distances, paralleling the disfavoring of CpG islands and gene start sites ([2, 11]. MLV, in contrast, strongly favors integration very near DNAseI hypersensitive sites [11].

CpG Islands

Analysis of integration sites with respect to the location of CpG islands within the genome (UCSC hg18 goldenpath database).

1. CpG Density, 1Mb, 100kb, and 10kb: We ask whether integration sites fall within regions more or less dense in CpG islands than would be expected for a random site in the genome. To do so we consider the density of CpG islands within the indicated genomic intervals surrounding each integration site. A red tile indicates that integration occurs in regions enriched for CpG islands. Blue indicates that integration favors regions poor in CpG islands compared to random. White indicates that integration sites show no bias for or against regions enriched in CpG islands.

    ROC area Calculation (e.g. CpG Density, 1Mb): We count the number of CpG islands falling in a 1Mb window surrounding each integration and MRC site (ie within 500 kb of a site). An integration site is scored:

    1 if more CpG islands lie in the 1Mb surrounding the integration site than in the same interval surrounding the compared MRC site,
    0 if fewer CpG islands lie in the genomic interval around the integration site,
    0.5 if same number of CpG islands fall in the interval surrounding each site.

    ROC area is the average score for all integration sites in the dataset.

    Statistical Test for difference from Random Controls (ROC area = 0.5): A one degree of freedom Wald test based on the variance mentioned above

2. <5kb and <1kb: Indicates whether integration sites fall more or less frequently within the indicated distance of a CpG island than would be expected for a random distribution (location of CpG islands from UCSC hg18 goldenpath database). Red indicates a preference for integration near a CpG island. Blue indicates that integration is disfavored near CpG islands. If the tile is white, the integration sites are no more or less likely to fall near CpG islands than matched random sites.

    ROC area Calculation (e.g. <5kb): An integration site is scored:

    1 if the integration site falls <5kb from a CpG island and the compared MRC site does not,
    0 if the MRC site is <5kb from a CpG island and the integration site is not,
    0.5 if both sites lie within or outside 1 Mb of a CpG island.

    ROC area is the average score for all integration sites in the dataset.

    Statistical Test for difference from Random Controls (ROC area = 0.5): A one degree of freedom Wald test based on the variance mentioned above

Significance: CpG islands are enriched in/near gene promoters, especially those of housekeeping genes, and are otherwise rare within the genome due to methylation and deamination of the cytosine. They are therefore markers of promoters when small genomic windows surrounding CpG islands are considered, and of gene dense regions when larger windows are considered. That is if a site is very close to a CpG island it is likely to be close to a gene promoter, and if a site is in a broad region enriched for CpG islands, it is likely to be in a more gene dense area of the genome.

Gene Density

Analysis of integration sites with respect to the local density of genes. We consider the indicated genomic interval surrounding each integration site (1Mb, 100kb, or 10kb) and ask whether integration sites fall within regions more or less dense in genes than would be expected for a random site in the genome. A red tile indicates that integration occurs in regions enriched for genes. Blue indicates that integration favors regions poor in genes compared to random. White indicates that integration sites show no bias for or against gene dense regions.

ROC area Calculation (e.g. Density 1Mb): We count the number of genes (RefSeq) falling in a 1Mb window surrounding each integration and matched random control site (i.e. within 500 kb of a site). An integration site is scored:

1 if more genes lie in this interval for the integration site than for the compared MRC site,
0 if fewer genes lie in the interval around the integration site, and
0.5 if same number of genes fall in a 1Mb interval surrounding each site.

ROC area is the average score for all integration sites in the dataset.

Statistical Test for difference from Random Controls (ROC area = 0.5): A one degree of freedom Wald test based on the variance mentioned above

Significance/Example: HIV has a known preference for integration in regions of the chromosome that are enriched for genes. This may be because it prefers to integrate within active transcription units [4]. However it may also be independently guided to regions of chromatin enriched for genes. In either case, this preference is hypothesized to afford HIV better access to transcription factors.

Expression Intensity

Analysis of integration sites with respect to the local density of sets of genes on an Affymetrix Gene Chip. We consider density of total genes as well as density of highly expressed genes. For this analysis we use microarray data measuring relative expression of genes in the cell type used in the study. We note that manipulations to cells in certain experiments may change gene expression profiles, and this must be considered in interpretation of the data.

1. All Genes Density, 1Mb: Calculations and tile interpretations are identical to those for "Density, 1Mb" in the Gene Density section with the exception that the "genes" counted are loci identified by expression probe sets on the relevant Affymetrix GeneChip.

2. Top ½ Expression, 1Mb: Calculations and tile interpretations are identical to those for "All Genes Density, 1Mb" with the exception that only the top ½ most expressed loci are counted.

3. Top 1/16th Expression, 1Mb: Calculations and tile interpretations are identical to those for "All Genes Density, 1Mb" with the exception that only the top 1/16th most expressed loci are counted.

Statistical Test for difference from Random Controls (ROC area = 0.5): A one degree of freedom Wald test based on the variance mentioned above

Significance: This analysis allows us to consider whether the preference for total genes is the same as the preference for highly expressed genes. This would be the case if the tile colors are the same for all three measures. If highly expressed genes are preferred more than are genes in general, the "Top 1/16th Expression" and "Top 1/2 expression" tiles may be red shifted from the "All Genes Density" tile.

GC Content

Analysis of integration sites with respect to the local GC content. A red tile indicates that for the genomic interval considered (see below for discussion of window size) integration sites lie in regions that are GC-rich compared to random sites in the genome. Blue indicates

that integration is disfavored in GC rich regions (favored in AT rich regions). White indicates that the vector shows no bias for GC content at the genomic interval considered.

ROC area Calculation: GC content is measured within the indicated interval surrounding each integration and matched random site. An integration site is ranked:

1 if the defined region surrounding an integration site is more GC rich than that surrounding the compared MRC site
0 if the integration site lies in a less GC rich region than the MRC site, and
0.5 if the integration and MRC sites are located in equally GC rich regions.

ROC area is the average rank for all integration sites in the dataset.

Statistical Test for difference from Random Controls (ROC area = 0.5): A one degree of freedom Wald test based on the variance mentioned above

Significance: GC content is positively correlated with genes and therefore when considering broad windows surrounding integration sites, a high GC content may indicate a preference for genes/gene dense regions. However, due to local sequence constraints that affect the choice of integration sites of many viruses, small windows surrounding integration sites may show different effects. For example, HIV prefers to integrate in gene dense regions and, predictably, GC content is high if measured in large windows surrounding HIV-1 integration sites in wild type cells. However if we consider only small windows surrounding integration sites, we find that HIV-1 prefers relatively AT-rich loci within those large GC rich regions. This is likely to be secondary to HIV-1's preference for integration on nucleosomes which position in relatively AT rich DNA [12]. The AT-hooks of LEDGF-p75, a known tether for HIV integrase, may also contribute to this effect [13]. For this reason, we display ROC area tiles for GC content in several genomic intervals.

References

1. Berry C, Hannenhalli S, Leipzig J, Bushman FD. (2006) Selection of target sites for mobile DNA integration in the human genome. PLoS Comput Biol 2(11): e157.

2. Mitchell RS, Beitzel BF, Schroder AR, Shinn P, Chen H, et al. (2004) Retroviral DNA integration: ASLV, HIV, and MLV show distinct target site preferences. PLoS Biol 2(8): E234.

3. Brady T, Lee YN, Ronen K, Malani N, Berry CC, et al. (2009) Integration target site selection by a resurrected human endogenous retrovirus. Genes Dev 23(5): 633-642.

4. Schroder AR, Shinn P, Chen H, Berry C, Ecker JR, et al. (2002) HIV-1 integration in the human genome favors active genes and local hotspots. Cell 110(4): 521-529.

5. Wu X, Li Y, Crise B, Burgess SM. (2003) Transcription start regions in the human genome are favored targets for MLV integration. Science 300(5626): 1749-1751.

6. Hacein-Bey-Abina S, Von Kalle C, Schmidt M, McCormack MP, Wulffraat N, et al. (2003) LMO2-associated clonal T cell proliferation in two patients after gene therapy for SCID-X1. Science 302(5644): 415-419.

7. Hacein-Bey-Abina S, von Kalle C, Schmidt M, Le Deist F, Wulffraat N, et al. (2003) A serious adverse event after successful gene therapy for X-linked severe combined immunodeficiency. N Engl J Med 348(3): 255-256.

8. Deichmann A, Hacein-Bey-Abina S, Schmidt M, Garrigue A, Brugman MH, et al. (2007) Vector integration is nonrandom and clustered and influences the fate of lymphopoiesis in SCID-X1 gene therapy. J Clin Invest 117(8): 2225-2232.

9. Hacein-Bey-Abina S, Garrigue A, Wang GP, Soulier J, Lim A, et al. (2008) Insertional oncogenesis in 4 patients after retrovirus-mediated gene therapy of SCID-X1. J Clin Invest 118(9): 3132-3142.

10. Stein S, Ott MG, Schultze-Strasser S, Jauch A, Burwinkel B, et al. (2010) Genomic instability and myelodysplasia with monosomy 7 consequent to EVI1 activation after gene therapy for chronic granulomatous disease. Nat Med 16(2): 198-204.

11. Lewinski MK, Yamashita M, Emerman M, Ciuffi A, Marshall H, et al. (2006) Retroviral DNA integration: Viral and cellular determinants of target-site selection. PLoS Pathog 2(6): e60.

12. Wang GP, Ciuffi A, Leipzig J, Berry CC, Bushman FD. (2007) HIV integration site selection: Analysis by massively parallel pyrosequencing reveals association with epigenetic modifications. Genome Res 17(8): 1186-1194.

13. Ciuffi A, Llano M, Poeschla E, Hoffmann C, Leipzig J, et al. (2005) A role for LEDGF/p75 in targeting HIV DNA integration. Nat Med 11(12): 1287-1289.
