# Supplementary material for: HIV Integration Targeting: A Pathway Involving Transportin-3 and the Nuclear Pore Protein RanBP2
Source: PLoS Pathog. 2011 Mar 10;7(3):e1001313. doi: 10.1371/journal.ppat.1001313 (PMC3053352; doi:10.1371/journal.ppat.1001313)
Supplement: Text S2 — Distributions of HIV integration sites after TNPO3 knockdown and rescue with siRNA insensitive allele. (0.58 MB PDF) [file ppat.1001313.s012.pdf]

Distributions of HIV integration sites after TNPO3 knockdown and rescue with siRNA insensitive allele

Charles C. Berry

January 3, 2011

Contents

1 Introduction **3**

2 Preference for Genes **5**
2.1 Acembly Genes 5
2.2 refGenes 7
2.3 ensGenes 8
2.4 genScan Genes 10
2.5 uniGenes 12
2.6 oncogenes 15

3 CpG Island Neighborhoods **16**
3.1 1 kilobase neighborhoods 16
3.2 2 kilobase neighborhoods 17
3.3 5 kilobase neighborhoods 18
3.4 10 kilobase neighborhoods 18
3.5 25 kilobase neighborhoods 19
3.6 50 kilobase neighborhoods 20

4 Gene Density, Expression 'Density', and CpG Island Density **22**
4.1 25 kilobase Window 22
4.2 50 kilobase Window 26
4.3 100 kilobase Window 31
4.4 250 kilobase Window 36
4.5 500 kilobase Window 41
4.6 1 megabase Window 46
4.7 2 megabase Window 51
4.8 4 megabase Window 56
4.9 8 megabase Window 61
4.10 16 megabase Window 66
4.11 32 megabase Window 71

5 Juxtaposition with Gene Start and End Positions **77**
5.1 Acembly Annotations 77
5.2 RefSeq Annotations 81
5.3 genScan Annotations 85
5.4 uniGene Annotations 89

6 GC content **93**

7 Cytobands **94**

1 Introduction

In this document, I examine the association of integration sites with various genomic features.

The data consist of both actual integration sites and sets of control sites, each set chosen to match the spacing (in bases) from the nearest restriction site (according to the direction in which the sequence was read) to an integration site. The numbers of insertion and matching sites for several data sets are shown below:

```
                  type
Origin.of.data.set insertion match
    siGL2               1226  2856
    siTNPO3-4            314   756
    siTNPO3-4+Resc      1492  3589
```

The advantage of choosing 'control' sites that match the spacing from the nearest restriction site is that biases due to location and density of restriction sites are eliminated by applying the classical multinomial logit model (reviewed in [2]). This model allows regression procedures to be applied to the study of integration intensity as a function of genomic features. The `clogit` function of the R `survival` library) implements estimation and fitting for such models along with the usual likelihood ratio and Wald tests.

The distribution of relative frequency of insertions across the chromosomes is given in this barplot:

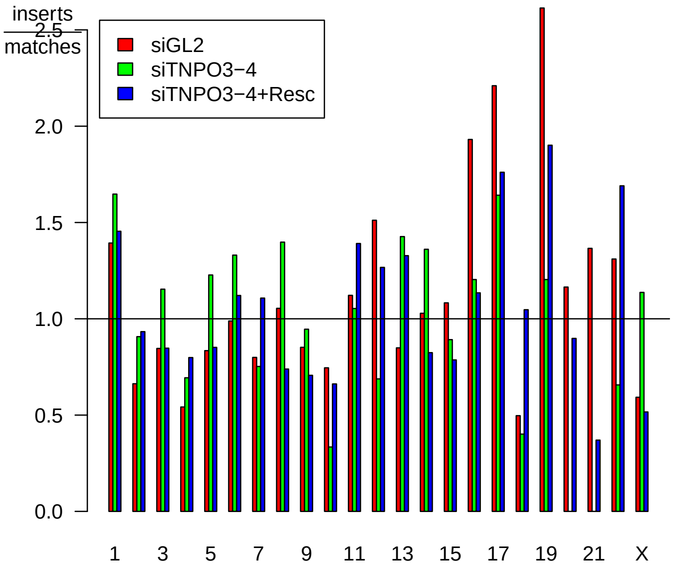

It seems evident that there are some chromosomes that are particularly favored for integration. This is reinforced by a test of statistical significance. The test performed used the likelihood ratio statistic for the multinomial logit model (reviewed in [2]) as implemented by the `clogit` function of the R `survival` library). The null hypothesis tested is that the ratio of true integration events to matched control sites is constant across all chromosomes. This test attains a p-value of $< 2.22e - 16$.

2 Preference for Genes

2.1 Acembly Genes

Here we examine the preference that integration events have for genes. In the following plot we show the relative frequency of integrations in genes according to the 'Acembly' annotation. The bars grouped over the label "In Gene" give the relative frequency of integration events (compared to control sites) between bases located within Acembly gene annotations, while the label "Not in Gene" give the relative frequency of integration events (compared to control sites) between bases not located within Acembly gene annotations.

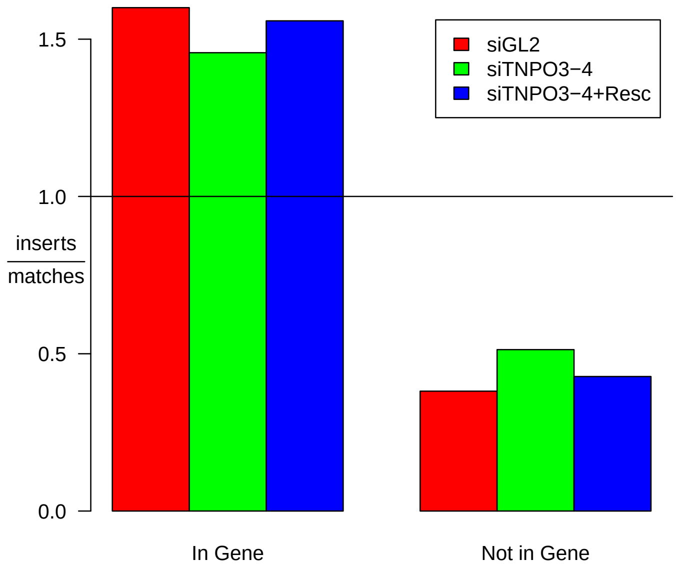

It seems evident that there is a strong tendency for insertions to occur in genes. A formal test of significance bears this out with a p-value of $< 2.22e-16$. Also, it appears that the tendency of different viruses to integrate into genes varies, and a test for this hypothesis attains 0.037701. Here is the table of coefficients of the log ratio of intensities for true insertion sites versus control insertion sites along with their standard errors, z statistics, and p-values for each data set:

	coef	se	z	p
siGL2	1.48	0.0876	16.90	9.75e-64
siTNPO3-4	1.03	0.1540	6.68	2.41e-11
siTNPO3-4+Resc	1.30	0.0744	17.50	1.58e-68

As is evident, there are some differences in the coefficients. The largest coefficient is seen in the siGL2 data set, while the smallest is seen in the siTNPO3-4 data set.

In the following plot we show the relative frequency of insertions in exons according to the 'Acembly' annotation. The bars grouped over the label "In Exon" give the relative frequency of integration events (compared to control sites) between bases located in exons according to the Acembly annotation, while the label "Not in Exon" give the relative frequency of integration events (compared to control sites) between bases not located in exons according to the Acembly gene annotation.

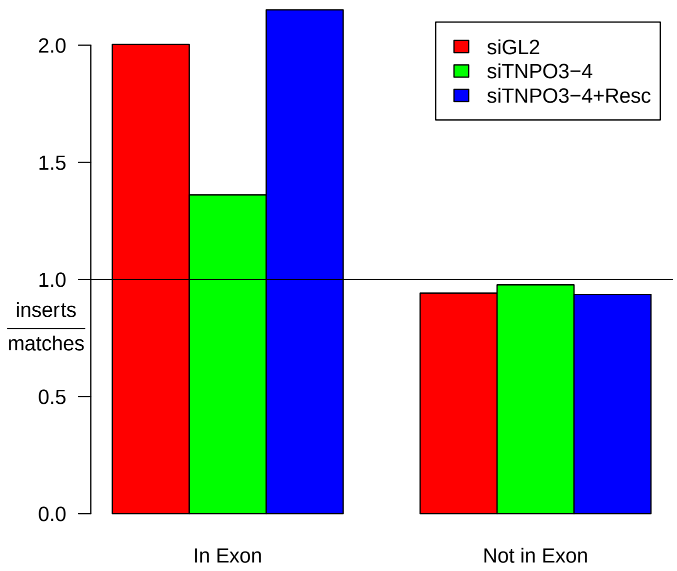

Here is the table of coefficients of the log ratio of intensities for true insertion sites versus control insertion sites along with their standard errors, z statistics, and p-values for each data set:

	coef	se	z	p
siGL2	0.254	0.129	1.970	0.04840
siTNPO3-4	-0.110	0.265	-0.414	0.67900
siTNPO3-4+Resc	0.338	0.115	2.950	0.00316

The model on which these coefficients are based include terms for whether the site is in a gene or not. Thus, the effect shown as "In Exon" is net of that due to being in a gene. Note that in the barplot above the 'Not in Exon' bars include both the introns and intergenic regions, so the impression given by the

table may differ from that for the barplot.

2.2 refGenes

Here we examine the preference that insertions have for genes. In the following plot we show the relative frequency of insertions in genes according to the 'refGene' annotation.

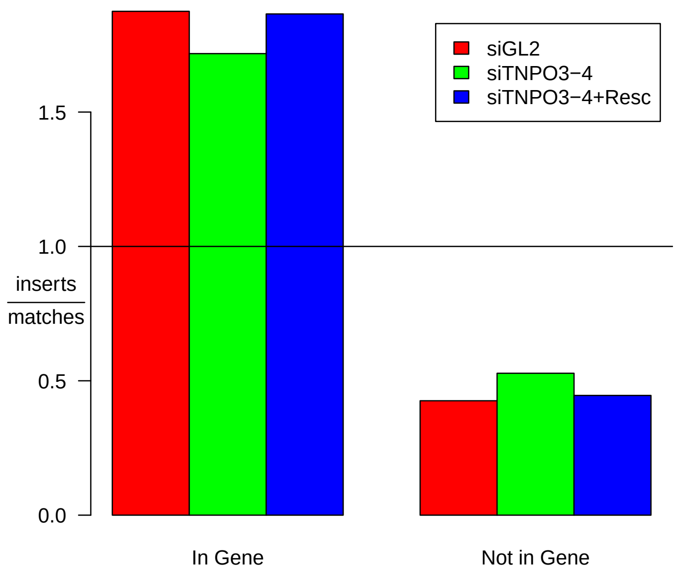

It seems evident that there is a strong tendency for insertions to occur in genes. A formal test of significance bears this out with a p-value of $< 2.22e-16$. Also, it appears that the tendency of different viruses to integrate into genes varies, and a test for this hypothesis attains 0.094082. Here is the table of coefficients of the log ratio of intensities for true insertion sites versus control insertion sites along with their standard errors, z statistics, and p-values for each data set:

	coef	se	z	p
siGL2	1.48	0.0798	18.50	2.04e-76
siTNPO3-4	1.13	0.1430	7.89	3.02e-15
siTNPO3-4+Resc	1.45	0.0714	20.20	3.70e-91

As is evident, there are some differences in the coefficients. The largest coefficient is seen in the siGL2 data set, while the smallest is seen in the siTNPO3-4 data set.

In the following plot we show the relative frequency of insertions in exons according to the 'refGene' annotation.

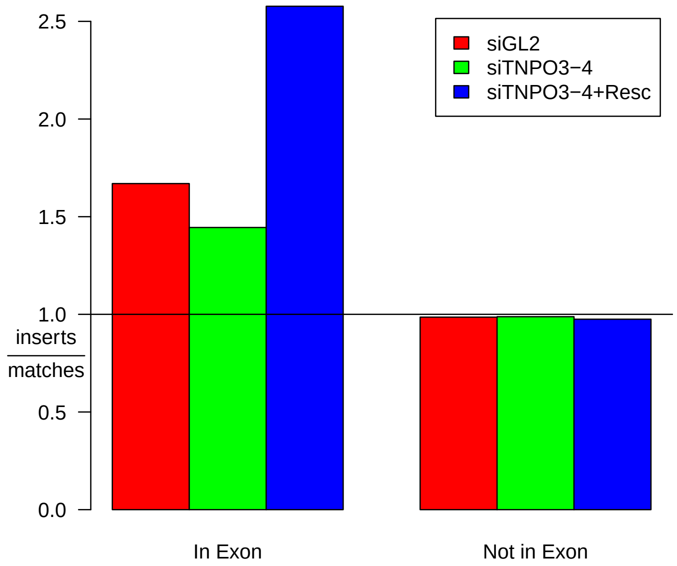

Here is the table of coefficients of the log ratio of intensities for true insertion sites versus control insertion sites along with their standard errors, z statistics, and p-values for each data set:

	coef	se	z	p
siGL2	-0.0813	0.208	-0.391	0.6960
siTNPO3-4	-0.2430	0.384	-0.632	0.5270
siTNPO3-4+Resc	0.3410	0.197	1.730	0.0835

The model on which these coefficients are based include terms for whether the site is in a gene or not. Thus, the effect shown as "In Exon" is net of that due to being in a gene.

2.3 ensGenes

Here we examine the preference that insertions have for genes. In the following plot we show the relative frequency of insertions in genes according to the 'ensGene' annotation.

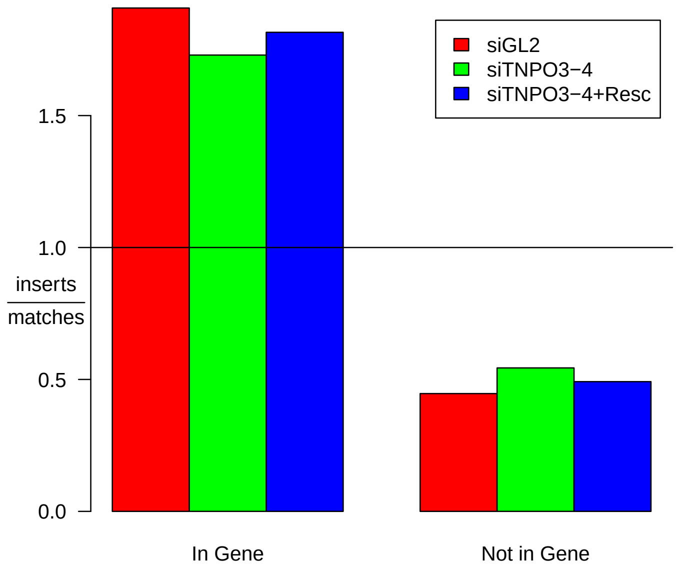

It seems evident that there is a strong tendency for insertions to occur in genes. A formal test of significance bears this out with a p-value of $< 2.22e-16$. Also, it appears that the tendency of different viruses to integrate into genes varies, and a test for this hypothesis attains 0.083195. Here is the table of coefficients of the log ratio of intensities for true insertion sites versus control insertion sites along with their standard errors, z statistics, and p-values for each data set:

	coef	se	z	p
siGL2	1.46	0.0793	18.50	3.19e-76
siTNPO3-4	1.12	0.1420	7.85	4.33e-15
siTNPO3-4+Resc	1.32	0.0695	19.00	1.92e-80

As is evident, there are some differences in the coefficients. The largest coefficient is seen in the siGL2 data set, while the smallest is seen in the siTNPO3-4 data set.

In the following plot we show the relative frequency of insertions in exons according to the 'ensGene' annotation.

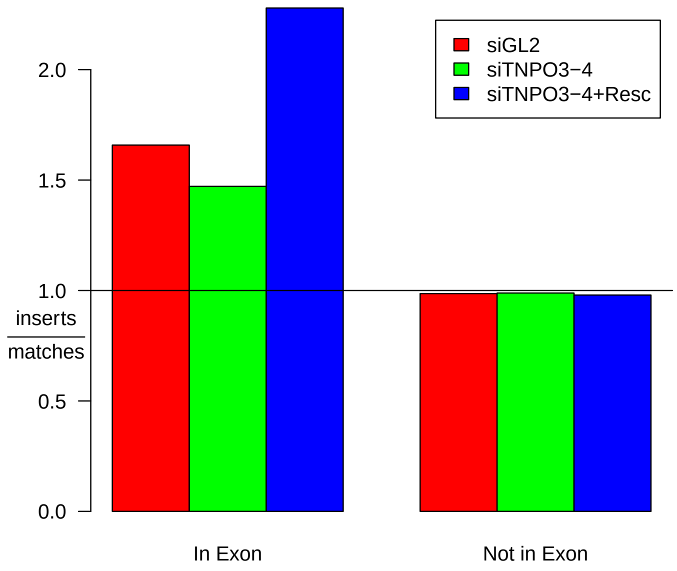

Here is the table of coefficients of the log ratio of intensities for true insertion sites versus control insertion sites along with their standard errors, z statistics, and p-values for each data set:

	coef	se	z	p
siGL2	-0.105	0.211	-0.497	0.619
siTNPO3-4	-0.179	0.396	-0.451	0.652
siTNPO3-4+Resc	0.210	0.200	1.050	0.293

The model on which these coefficients are based include terms for whether the site is in a gene or not. Thus, the effect shown as "In Exon" is net of that due to being in a gene.

2.4 genScan Genes

Here we examine the preference that insertions have for genes. In the following plot we show the relative frequency of insertions in genes according to the 'genScan' annotation.

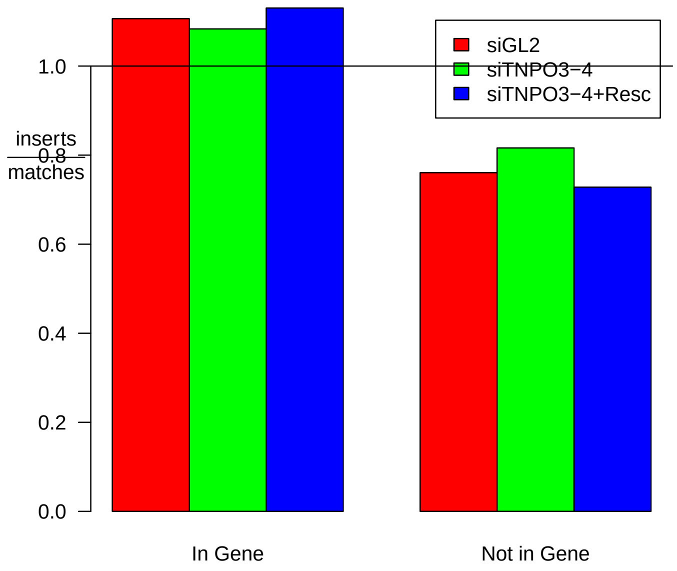

It seems evident that there is a strong tendency for insertions to occur in genes. A formal test of significance bears this out with a p-value of $3.0965e-16$. Also, it appears that the tendency of different viruses to integrate into genes varies, and a test for this hypothesis attains 0.55462. Here is the table of coefficients of the log ratio of intensities for true insertion sites versus control insertion sites along with their standard errors, z statistics, and p-values for each data set:

	coef	se	z	p
siGL2	0.374	0.0791	4.73	2.29e-06
siTNPO3-4	0.284	0.1530	1.86	6.32e-02
siTNPO3-4+Resc	0.450	0.0716	6.29	3.09e-10

As is evident, there are some differences in the coefficients. The largest coefficient is seen in the siTNPO3-4+Resc data set, while the smallest is seen in the siTNPO3-4 data set.

In the following plot we show the relative frequency of insertions in exons according to the 'genScan' annotation.

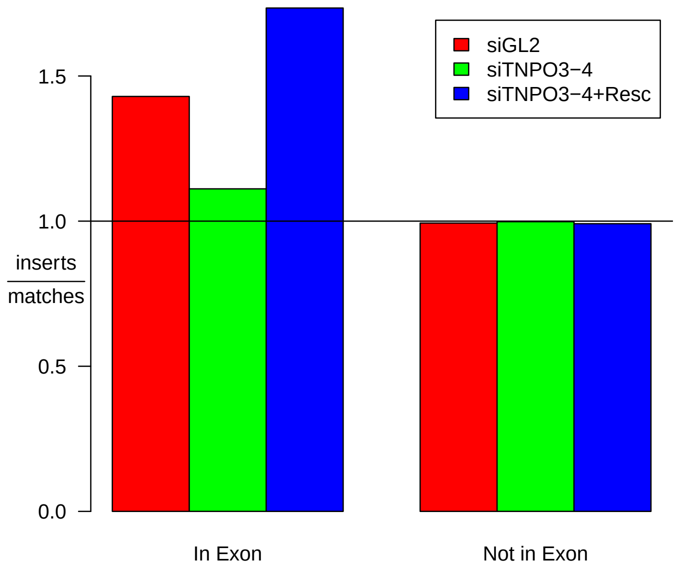

Here is the table of coefficients of the log ratio of intensities for true insertion sites versus control insertion sites along with their standard errors, z statistics, and p-values for each data set:

	coef	se	z	p
siGL2	0.2230	0.247	0.905	0.3650
siTNPO3-4	-0.0618	0.497	-0.124	0.9010
siTNPO3-4+Resc	0.4210	0.241	1.740	0.0814

The model on which these coefficients are based include terms for whether the site is in a gene or not. Thus, the effect shown as ”In Exon” is net of that due to being in a gene.

2.5 uniGenes

Here we examine the preference that insertions have for genes. In the following plot we show the relative frequency of insertions in genes according to the ’uniGene’ annotation.

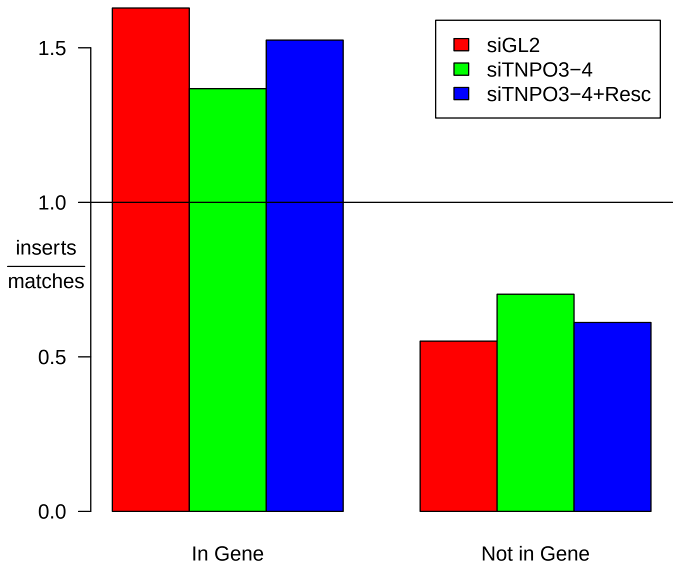

It seems evident that there is a strong tendency for insertions to occur in genes. A formal test of significance bears this out with a p-value of $< 2.22e-16$. Also, it appears that the tendency of different viruses to integrate into genes varies, and a test for this hypothesis attains 0.015377. Here is the table of coefficients of the log ratio of intensities for true insertion sites versus control insertion sites along with their standard errors, z statistics, and p-values for each data set:

	coef	se	z	p
siGL2	1.090	0.0746	14.60	3.28e-48
siTNPO3-4	0.644	0.1380	4.67	2.98e-06
siTNPO3-4+Resc	0.931	0.0661	14.10	4.32e-45

As is evident, there are some differences in the coefficients. The largest coefficient is seen in the siGL2 data set, while the smallest is seen in the siTNPO3-4 data set.

In the following plot we show the relative frequency of insertions in exons according to the 'uniGene' annotation.

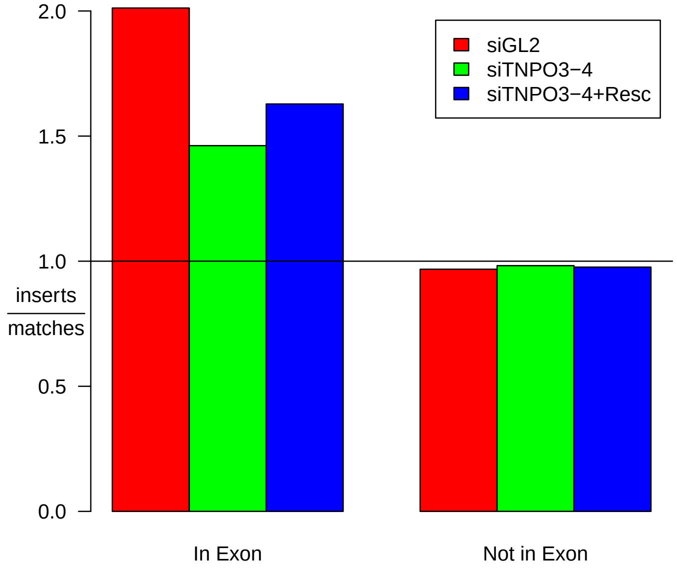

Here is the table of coefficients of the log ratio of intensities for true insertion sites versus control insertion sites along with their standard errors, z statistics, and p-values for each data set:

	coef	se	z	p
siGL2	0.2760	0.168	1.640	0.100
siTNPO3-4	0.0366	0.326	0.112	0.911
siTNPO3-4+Resc	0.0752	0.147	0.512	0.609

The model on which these coefficients are based include terms for whether the site is in a gene or not. Thus, the effect shown as "In Exon" is net of that due to being in a gene.

2.6 oncogenes

Here we examine the preference that insertions have for oncogenes. In the following plot we show the relative frequency of insertions with 50kb of an oncogene 5' end.

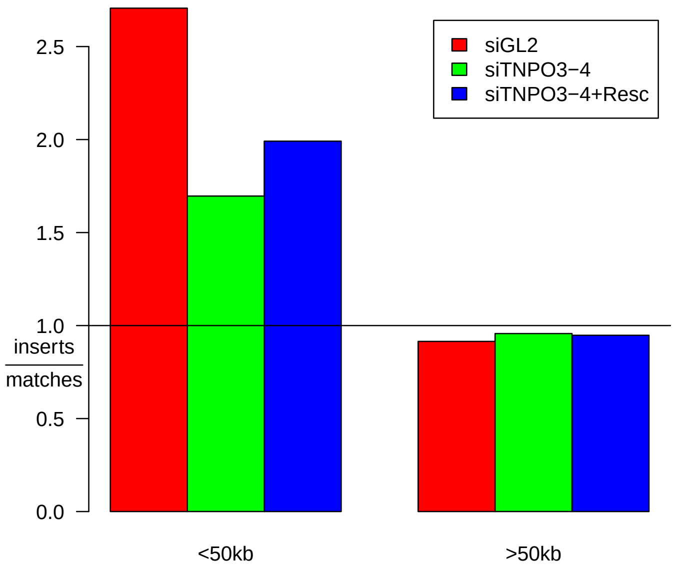

A formal test of oncogenic insertion returns p-value of $< 2.22e - 16$. The tendency of different viruses to integrate near oncogenes may vary, and a test for this hypothesis attains 0.035113. Here is the table of coefficients of the log ratio of intensities for true insertion sites versus control insertion sites along with their standard errors, z statistics, and p-values for each data set:

	coef	se	z	p
siGL2	-1.110	0.127	-8.75	2.04e-18
siTNPO3-4	-0.576	0.251	-2.30	2.15e-02
siTNPO3-4+Resc	-0.716	0.116	-6.17	6.93e-10
siGL2	NA	0.000	NA	NA
siTNPO3-4	NA	0.000	NA	NA
siTNPO3-4+Resc	NA	0.000	NA	NA

As is evident, there are some differences in the coefficients. The largest coefficient is seen in the siTNPO3-4 data set, while the smallest is seen in the siGL2 data set.

3 CpG Island Neighborhoods

Here we study the effect of being in the neighborhood of CpG Islands. Following Wu et al [3], who found that the neighborhoods within ±1kb of CpG islands are enriched for MLV insertions, we study such neighborhoods.

3.1 1 kilobase neighborhoods

The following plot shows the effect of being in or within ±1kb of a CpG island:

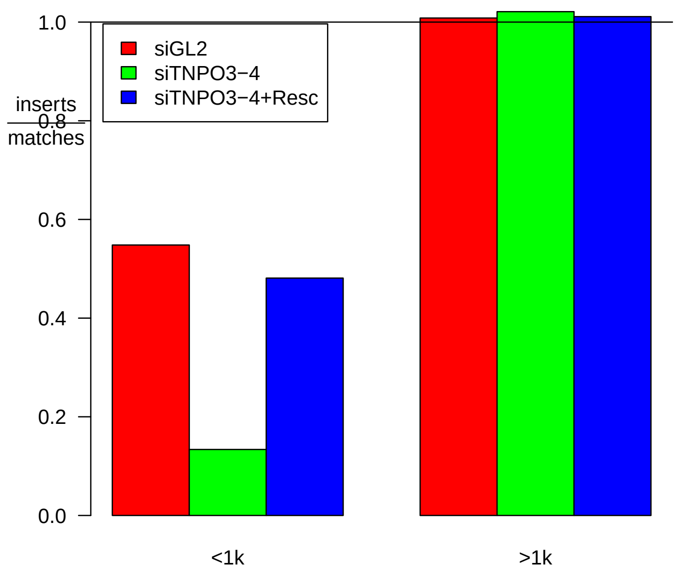

A formal test of significance comparing the difference attains a p-value of $4.7283e-05$. A test for differences between viruses attains 0.33821. Here is the table of coefficients of the log ratio of intensities for true insertion sites versus control insertion sites along with their standard errors, z statistics, and p-values for each data set:

	coef	se	z	p
siGL2	-0.611	0.322	-1.90	0.05750
siTNPO3-4	-1.960	1.030	-1.90	0.05690
siTNPO3-4+Resc	-0.745	0.284	-2.63	0.00861

The largest coefficient is seen in the siGL2 data set, while the smallest is seen in the siTNPO3-4 data set.

3.2 2 kilobase neighborhoods

The following plot shows the effect of being in or within ±2kb of a CpG island:

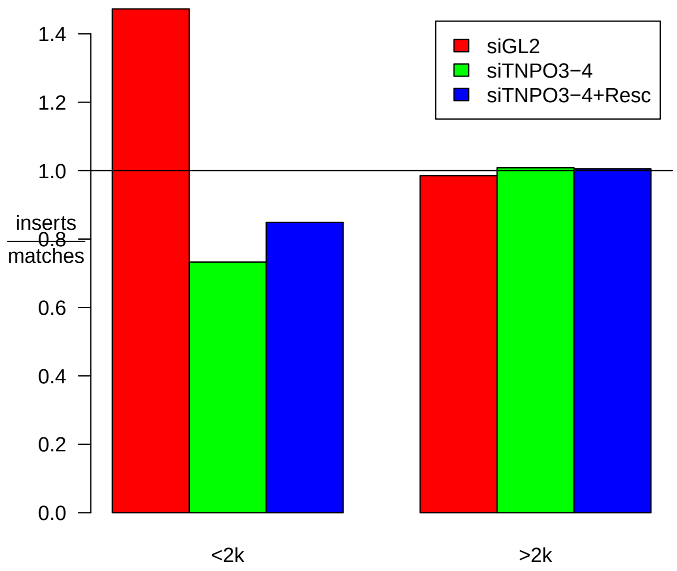

A formal test of significance comparing the difference attains a p-value of 0.57773. A test for differences between viruses attains 0.044963. Here is the table of coefficients of the log ratio of intensities for true insertion sites versus control insertion sites along with their standard errors, z statistics, and p-values for each data set:

	coef	se	z	p
siGL2	0.396	0.176	2.250	0.0244
siTNPO3-4	-0.305	0.433	-0.703	0.4820
siTNPO3-4+Resc	-0.188	0.183	-1.030	0.3050

The largest coefficient is seen in the siGL2 data set, while the smallest is seen in the siTNPO3-4 data set.

3.3 5 kilobase neighborhoods

The following plot shows the effect of being in or within ±5kb of a CpG island:

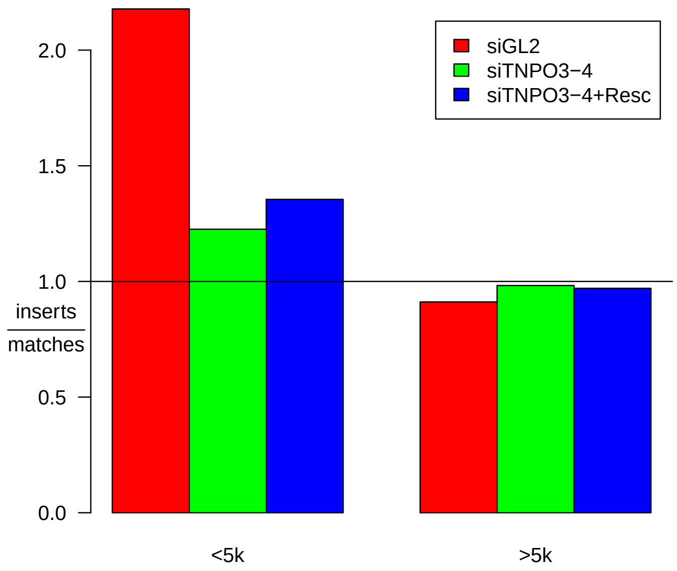

A formal test of significance comparing the difference attains a p-value of $3.6569e-14$. A test for differences between viruses attains 0.00016490. Here is the table of coefficients of the log ratio of intensities for true insertion sites versus control insertion sites along with their standard errors, z statistics, and p-values for each data set:

	coef	se	z	p
siGL2	0.895	0.110	8.15	3.63e-16
siTNPO3-4	0.205	0.241	0.85	3.95e-01
siTNPO3-4+Resc	0.308	0.105	2.95	3.19e-03

The largest coefficient is seen in the siGL2 data set, while the smallest is seen in the siTNPO3-4 data set.

3.4 10 kilobase neighborhoods

The following plot shows the effect of being in or within ±10kb of a CpG island:

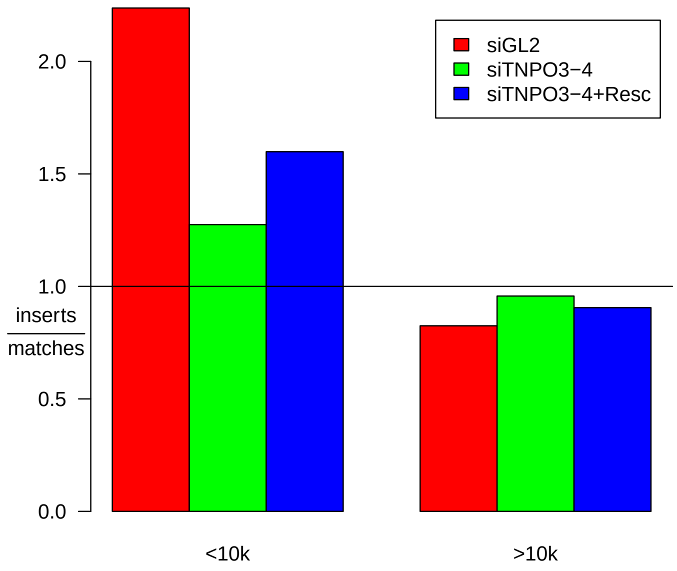

A formal test of significance comparing the difference attains a p-value of $< 2.22e - 16$. A test for differences between viruses attains $1.7387e - 05$. Here is the table of coefficients of the log ratio of intensities for true insertion sites versus control insertion sites along with their standard errors, z statistics, and p-values for each data set:

	coef	se	z	p
siGL2	1.000	0.0875	11.50	2.29e-30
siTNPO3-4	0.268	0.1840	1.46	1.45e-01
siTNPO3-4+Resc	0.536	0.0796	6.73	1.66e-11

The largest coefficient is seen in the siGL2 data set, while the smallest is seen in the siTNPO3-4 data set.

3.5 25 kilobase neighborhoods

The following plot shows the effect of being in or within ±25kb of a CpG island:

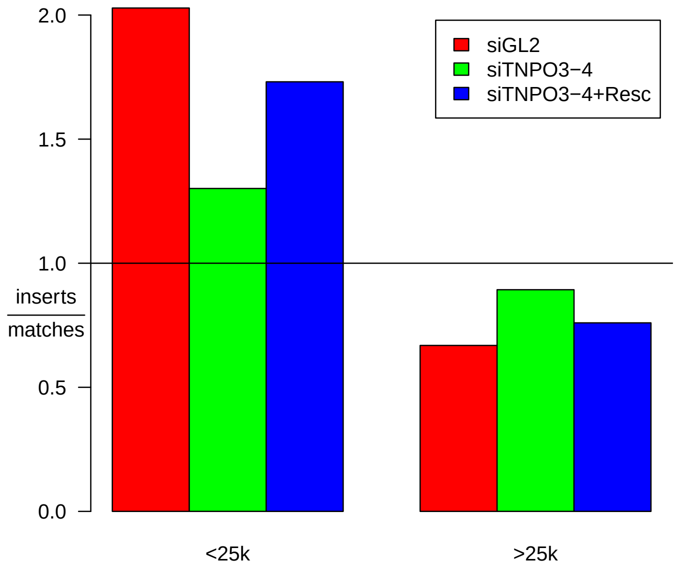

A formal test of significance comparing the difference attains a p-value of $< 2.22e - 16$. A test for differences between viruses attains $8.0664e - 06$. Here is the table of coefficients of the log ratio of intensities for true insertion sites versus control insertion sites along with their standard errors, z statistics, and p-values for each data set:

	coef	se	z	p
siGL2	1.100	0.0745	14.80	1.09e-49
siTNPO3-4	0.364	0.1460	2.49	1.28e-02
siTNPO3-4+Resc	0.790	0.0658	12.00	3.76e-33

The largest coefficient is seen in the siGL2 data set, while the smallest is seen in the siTNPO3-4 data set.

3.6 50 kilobase neighborhoods

The following plot shows the effect of being in or within ±50kb of a CpG island:

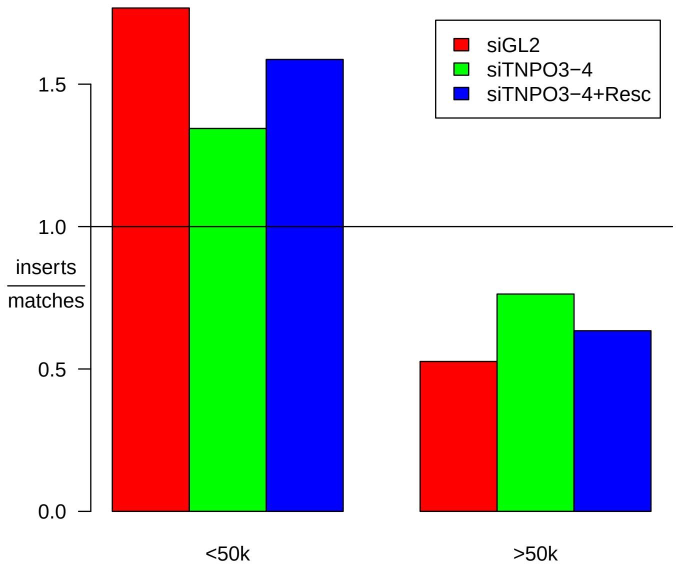

A formal test of significance comparing the difference attains a p-value of $< 2.22e - 16$. A test for differences between viruses attains $2.2027e - 05$. Here is the table of coefficients of the log ratio of intensities for true insertion sites versus control insertion sites along with their standard errors, z statistics, and p-values for each data set:

	coef	se	z	p
siGL2	1.210	0.0753	16.10	2.95e-58
siTNPO3-4	0.542	0.1380	3.94	8.11e-05
siTNPO3-4+Resc	0.892	0.0644	13.90	1.05e-43

The largest coefficient is seen in the siGL2 data set, while the smallest is seen in the siTNPO3-4 data set.

4 Gene Density, Expression 'Density', and CpG Island Density

In this section the association with gene density is examined. For expression analysis, the 'genes' that are counted are the genes represented on the microarray. In addition, we the number of such genes expressed at various levels. The levels are

low.ex Count genes whose expression is in the upper half and divide by number of bases

med.ex Count genes whose expression is in the upper $1/8^{th}$ and divide by number of bases

high.ex Count genes whose expression is in the upper $1/16^{th}$ and divide by number of bases

The bolded terms are used as abbreviations in what follows. The abbreviation **dens** is used to indicate gene density as number of genes per base.

4.1 25 kilobase Window

In the barplot that follows we examine the association of insertion sites with gene density in a 25 kilobase window surrounding each locus. More such plots will follow and the method of their construction is always to try to divide the data according to the deciles of density. However, it often happens that there is a very skewed distribution of density and even the 90th percentile is zero. In that case, the barplots simply show the sites for which the density is zero and those for which it is non-zero. If there are fewer than ten groups of bars, the groupings contain ten percent of the sites each except for the leftmost grouping which will contain all of the remaining sites.

Also note that the title of the plot contains clues as to its content; the prefix indicates the type of variable studied while the suffix indicates the window width in the number of bases. The p-value given is the result of fitting a cubic polynomial to the gene density values.

The following expression data and probe set were used for this report:

```
[1] "ledgf293TS-HU133Plus2"

[1] "HG-U133"

                 coef     se     z        p
siGL2           1.090 0.0737 14.80 1.15e-49
siTNPO3-4       0.587 0.1420  4.13 3.57e-05
siTNPO3-4+Resc  0.940 0.0669 14.00 7.92e-45
```

Here are the results for expression density. First, we count just genes that are in the upper half.

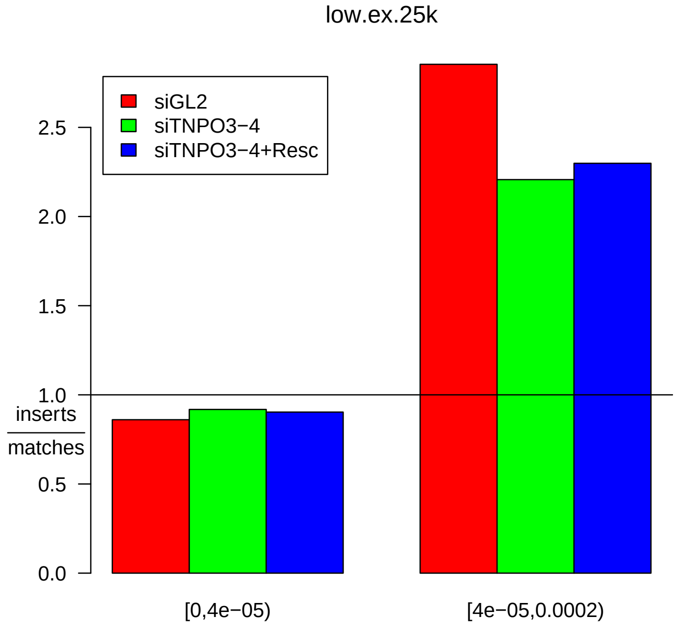

	coef	se	z	p
siGL2	1.440	0.0857	16.80	2.63e-63
siTNPO3-4	0.964	0.1670	5.77	8.01e-09
siTNPO3-4+Resc	1.090	0.0753	14.50	1.48e-47

Now we count genes in the upper $1/8^{th}$:

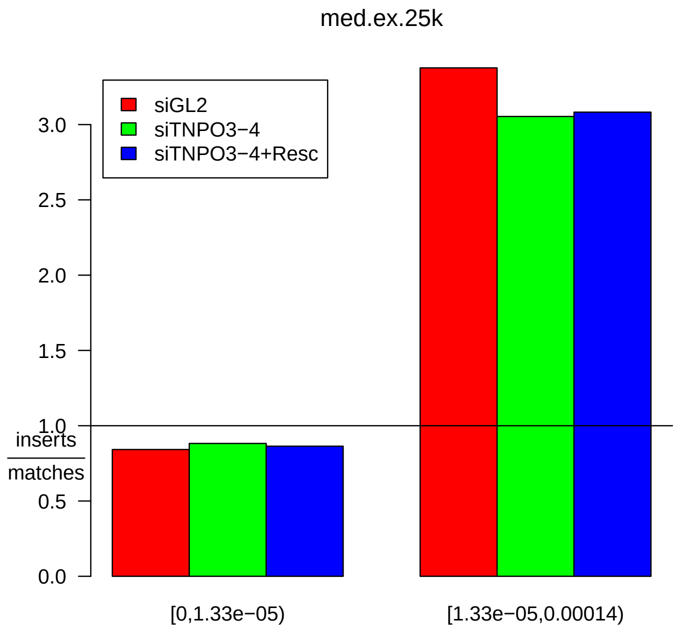

	coef	se	z	p
siGL2	1.57	0.1040	15.10	1.96e-51
siTNPO3-4	1.21	0.2060	5.86	4.51e-09
siTNPO3-4+Resc	1.36	0.0917	14.80	9.73e-50

And here we count genes in the upper $1/16^{th}$:

```
Density data too sparse for barplot

                coef    se     z         p
siGL2           1.45 0.128 11.30 9.30e-30
siTNPO3-4       1.24 0.263  4.73 2.30e-06
siTNPO3-4+Resc  1.29 0.115 11.20 2.74e-29
```

Here the effect of density of CpG islands is studied:

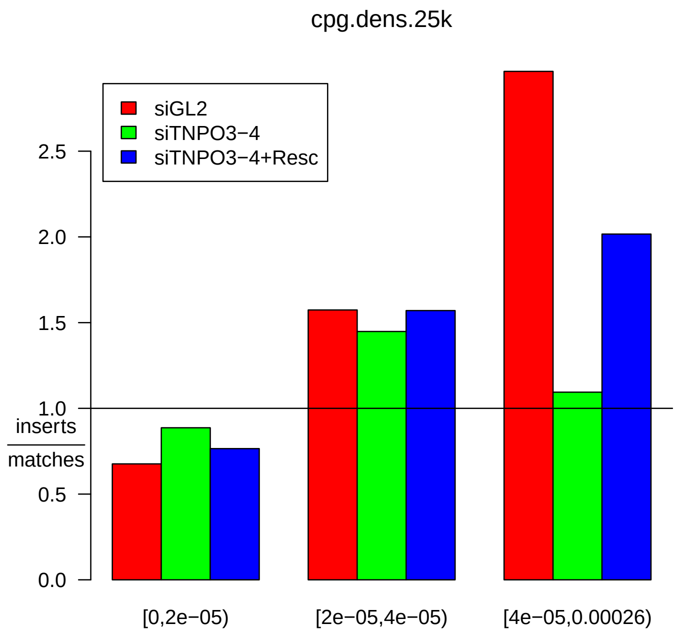


```
                coef     se     z        p
siGL2          1.090 0.0744 14.60 3.30e-48
siTNPO3-4      0.389 0.1460  2.66 7.76e-03
siTNPO3-4+Resc 0.780 0.0659 11.80 2.82e-32
```

4.2 50 kilobase Window

In the barplot that follows we examine the association of insertion sites with expression density in a 50 kilobase window surrounding each locus. First, we count just the number of genes represented on the chip.

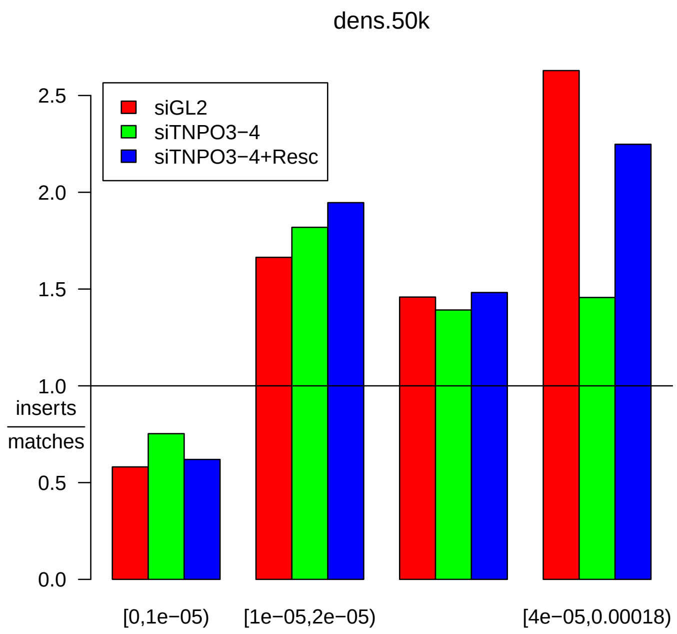

	coef	se	z	p
siGL2	1.220	0.0744	16.40	2.47e-60
siTNPO3-4	0.767	0.1370	5.58	2.42e-08
siTNPO3-4+Resc	1.090	0.0660	16.60	7.04e-62

Here are the results for expression density. First, we count just genes that are in the upper half.

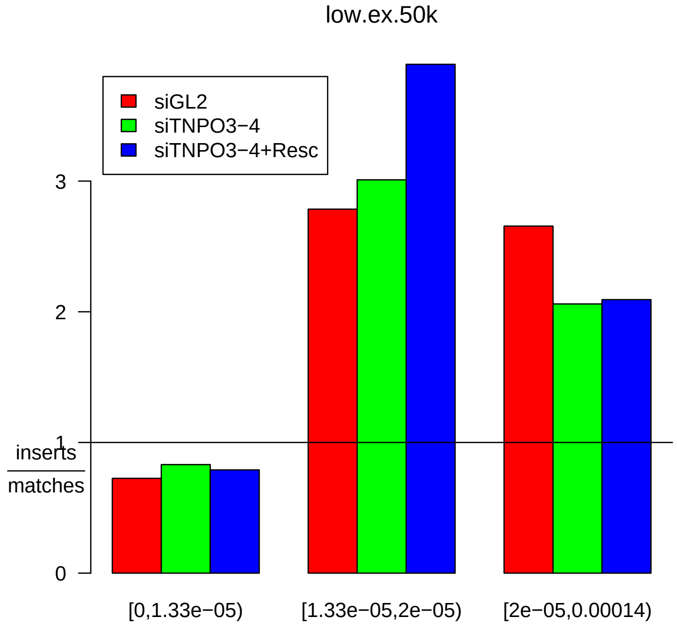

	coef	se	z	p
siGL2	1.50	0.0778	19.30	4.84e-83
siTNPO3-4	1.01	0.1470	6.82	9.13e-12
siTNPO3-4+Resc	1.14	0.0670	17.10	2.06e-65

Now we count genes in the upper $1/8^{th}$:

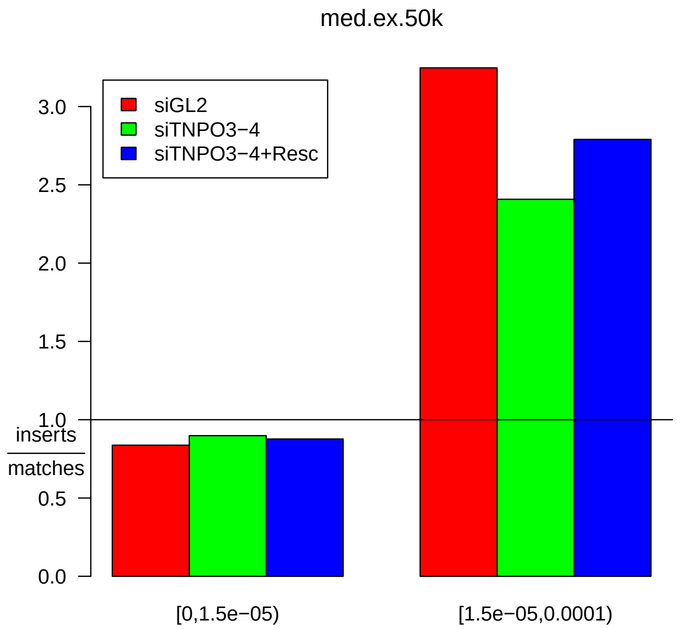

	coef	se	z	p
siGL2	1.59	0.0882	18.00	2.12e-72
siTNPO3-4	1.15	0.1680	6.85	7.29e-12
siTNPO3-4+Resc	1.28	0.0749	17.00	3.79e-65

And here we count genes in the upper $1/16^{th}$:

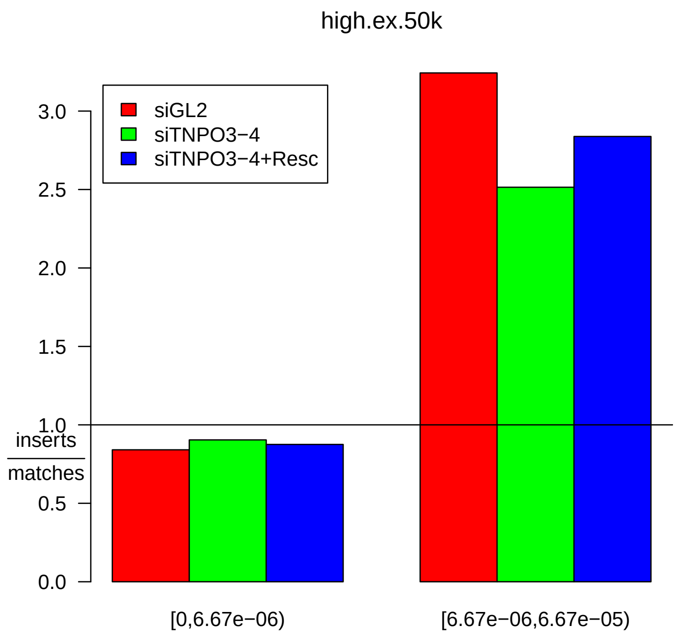

	coef	se	z	p
siGL2	1.410	0.0999	14.10	2.92e-45
siTNPO3-4	0.945	0.2060	4.59	4.37e-06
siTNPO3-4+Resc	1.190	0.0894	13.30	2.56e-40

Here the effect of density of CpG islands is studied:

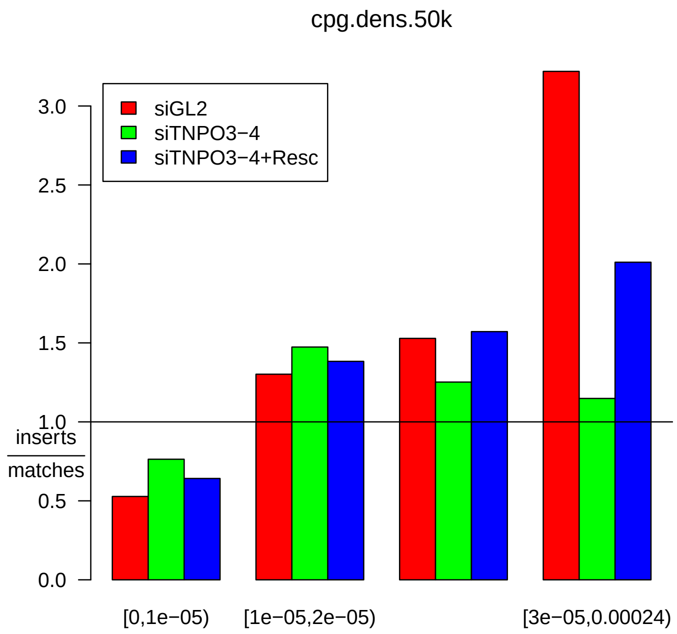

	coef	se	z	p
siGL2	1.220	0.0753	16.20	1.04e-58
siTNPO3-4	0.543	0.1370	3.96	7.54e-05
siTNPO3-4+Resc	0.878	0.0643	13.70	1.80e-42

4.3 100 kilobase Window

In the barplot that follows we examine the association of insertion sites with expression density in a 100 kilobase window surrounding each locus. First, we count just the number of genes represented on the chip.

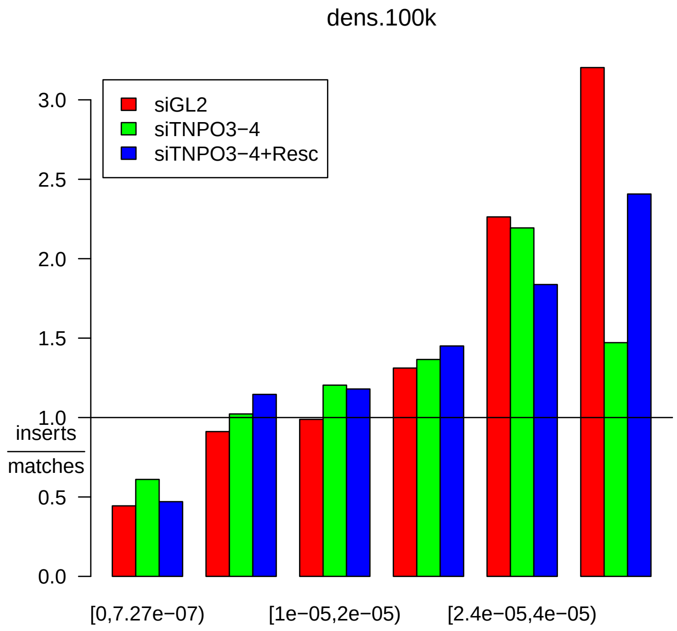

	coef	se	z	p
siGL2	1.260	0.0741	17.00	9.92e-65
siTNPO3-4	0.855	0.1470	5.81	6.31e-09
siTNPO3-4+Resc	0.962	0.0647	14.90	6.21e-50

Here are the results for expression density. First, we count just genes that are in the upper half.

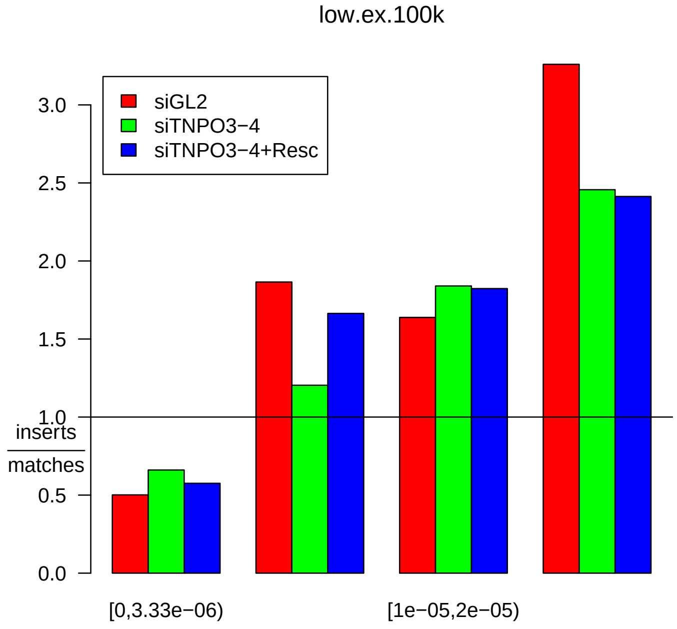

	coef	se	z	p
siGL2	1.49	0.0773	19.30	1.22e-82
siTNPO3-4	1.06	0.1430	7.38	1.64e-13
siTNPO3-4+Resc	1.23	0.0663	18.50	2.03e-76

Now we count genes in the upper $1/8^{th}$:

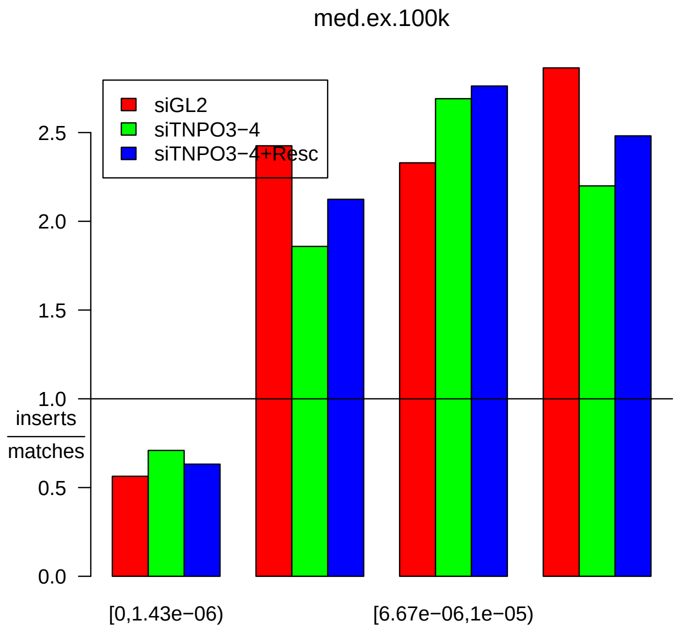

	coef	se	z	p
siGL2	1.59	0.0802	19.90	9.42e-88
siTNPO3-4	1.15	0.1530	7.51	5.70e-14
siTNPO3-4+Resc	1.31	0.0686	19.10	2.90e-81

And here we count genes in the upper $1/16^{th}$:

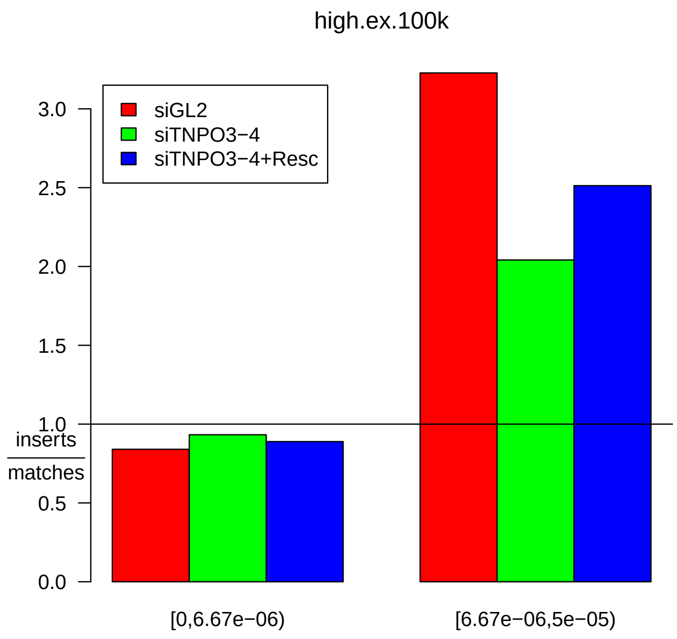

	coef	se	z	p
siGL2	1.44	0.0863	16.70	1.01e-62
siTNPO3-4	1.00	0.1780	5.64	1.68e-08
siTNPO3-4+Resc	1.18	0.0760	15.60	1.05e-54

Here the effect of density of CpG islands is studied:

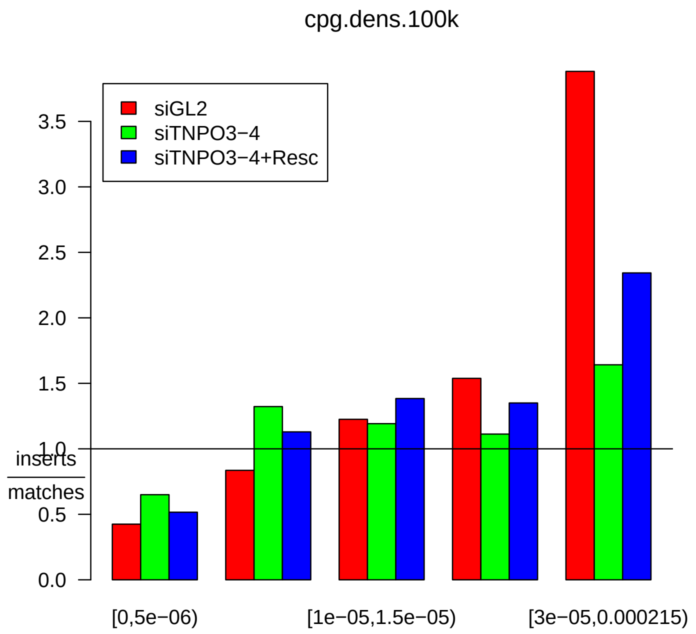

	coef	se	z	p
siGL2	1.260	0.0754	16.70	1.70e-62
siTNPO3-4	0.344	0.1410	2.45	1.45e-02
siTNPO3-4+Resc	0.801	0.0640	12.50	6.78e-36

4.4 250 kilobase Window

In the barplot that follows we examine the association of insertion sites with expression density in a 250 kilobase window surrounding each locus. First, we count just the number of genes represented on the chip.

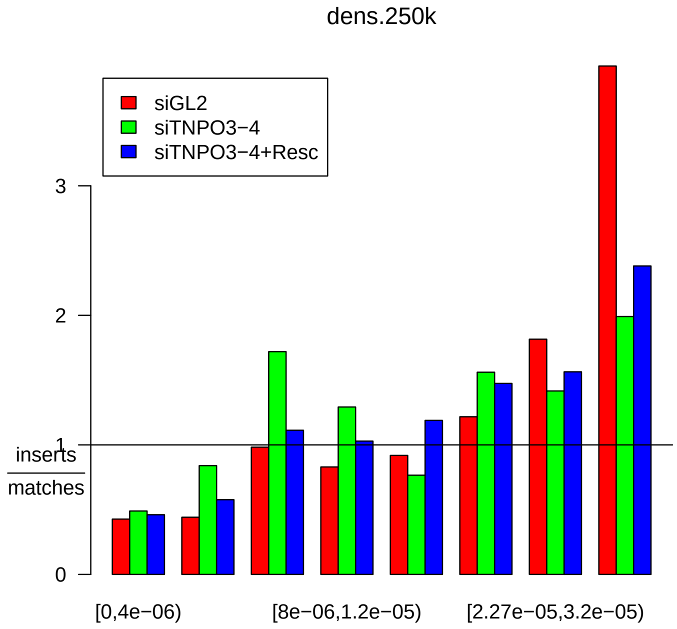

	coef	se	z	p
siGL2	1.180	0.0752	15.70	2.11e-55
siTNPO3-4	0.598	0.1340	4.46	8.12e-06
siTNPO3-4+Resc	0.964	0.0648	14.90	5.47e-50

Here are the results for expression density. First, we count just genes that are in the upper half.

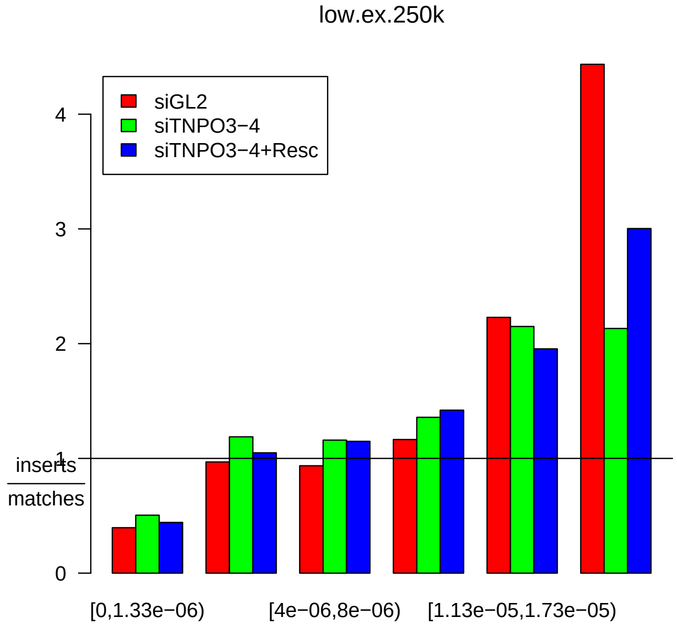

	coef	se	z	p
siGL2	1.340	0.0745	18.00	4.35e-72
siTNPO3-4	0.827	0.1360	6.06	1.33e-09
siTNPO3-4+Resc	1.030	0.0645	16.00	2.21e-57

Now we count genes in the upper $1/8^{th}$:

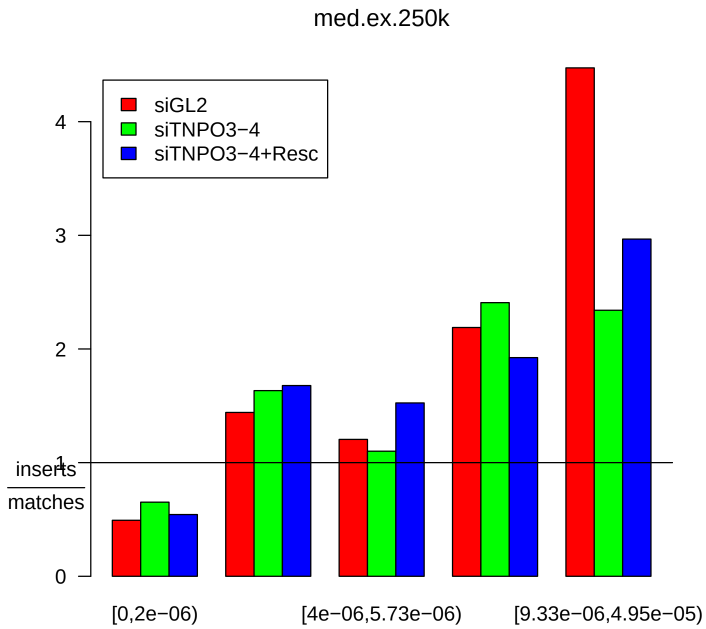


```
                coef     se     z        p
siGL2          1.480 0.0802 18.50 4.37e-76
siTNPO3-4      0.906 0.1420  6.39 1.67e-10
siTNPO3-4+Resc 1.320 0.0681 19.40 4.95e-84
```

And here we count genes in the upper $1/16^{th}$:

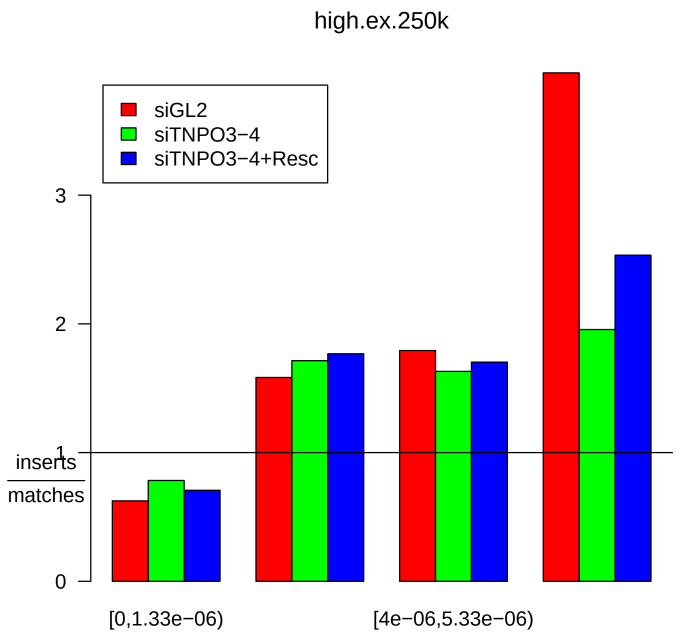

	coef	se	z	p
siGL2	1.380	0.0765	18.00	1.60e-72
siTNPO3-4	0.818	0.1430	5.72	1.05e-08
siTNPO3-4+Resc	1.110	0.0665	16.60	4.04e-62

Here the effect of density of CpG islands is studied:

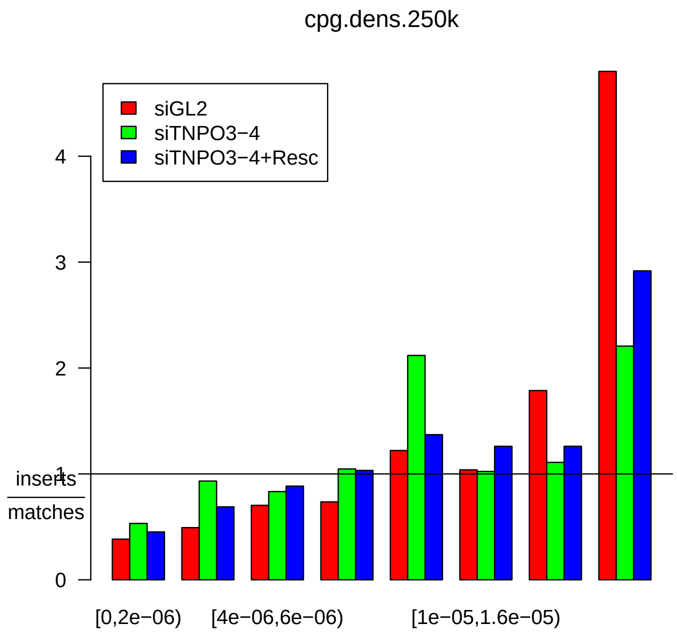

	coef	se	z	p
siGL2	1.290	0.0766	16.80	1.33e-63
siTNPO3-4	0.591	0.1380	4.27	1.96e-05
siTNPO3-4+Resc	0.805	0.0639	12.60	1.95e-36

4.5 500 kilobase Window

In the barplot that follows we examine the association of insertion sites with expression density in a 500 kilobase window surrounding each locus. First, we count just the number of genes represented on the chip.

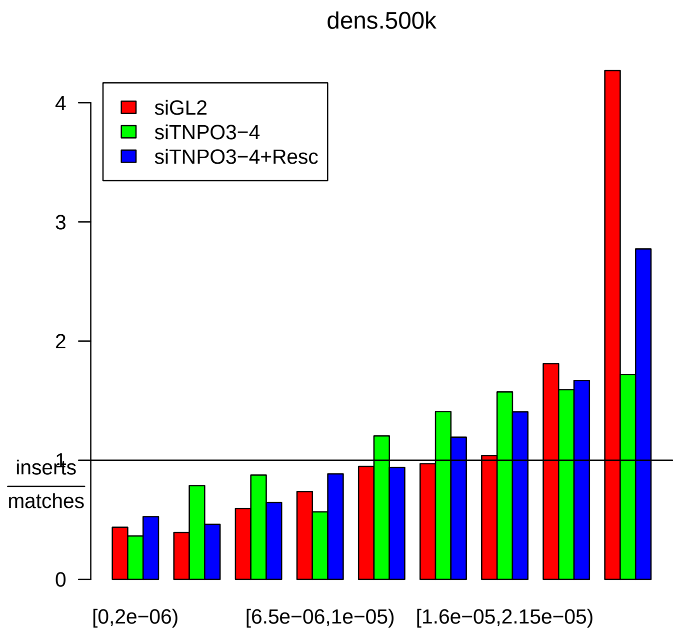

	coef	se	z	p
siGL2	1.160	0.0755	15.30	6.86e-53
siTNPO3-4	0.863	0.1390	6.22	4.88e-10
siTNPO3-4+Resc	0.910	0.0647	14.10	5.94e-45

Here are the results for expression density. First, we count just genes that are in the upper half.

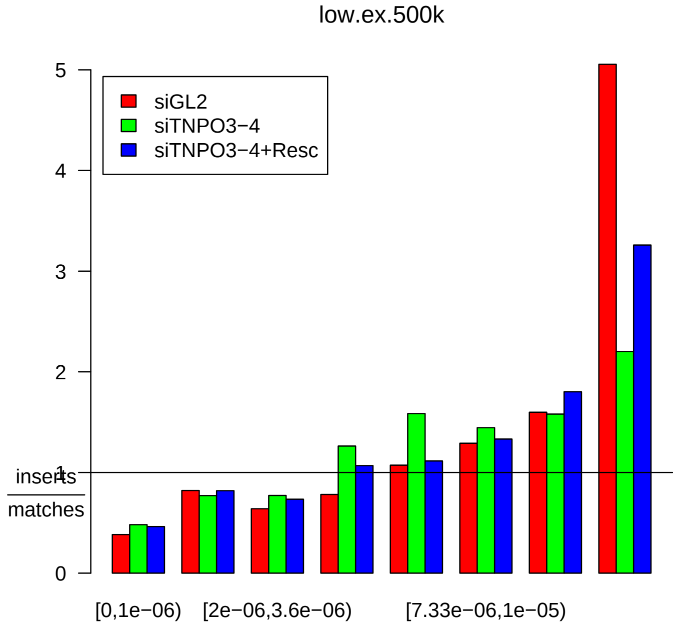

	coef	se	z	p
siGL2	1.200	0.0763	15.7	2.28e-55
siTNPO3-4	0.931	0.1430	6.5	7.81e-11
siTNPO3-4+Resc	0.971	0.0659	14.7	4.41e-49

Now we count genes in the upper $1/8^{th}$:

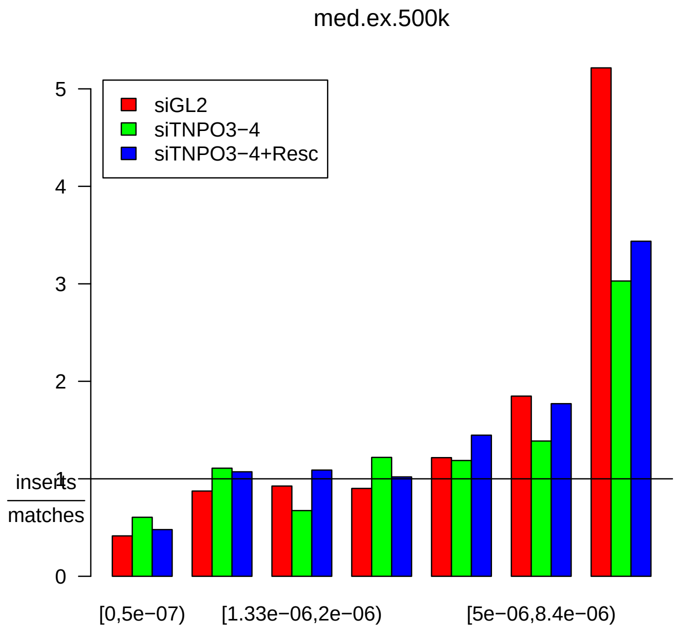

	coef	se	z	p
siGL2	1.250	0.0775	16.20	6.71e-59
siTNPO3-4	0.641	0.1380	4.66	3.23e-06
siTNPO3-4+Resc	0.978	0.0663	14.80	2.39e-49

And here we count genes in the upper $1/16^{th}$:

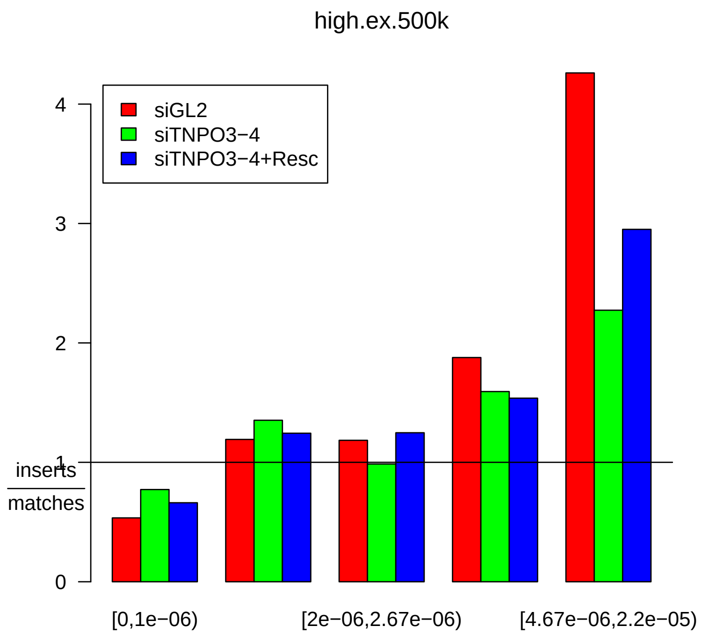

	coef	se	z	p
siGL2	1.280	0.0768	16.60	4.87e-62
siTNPO3-4	0.600	0.1390	4.33	1.51e-05
siTNPO3-4+Resc	0.912	0.0654	14.00	2.92e-44

Here the effect of density of CpG islands is studied:

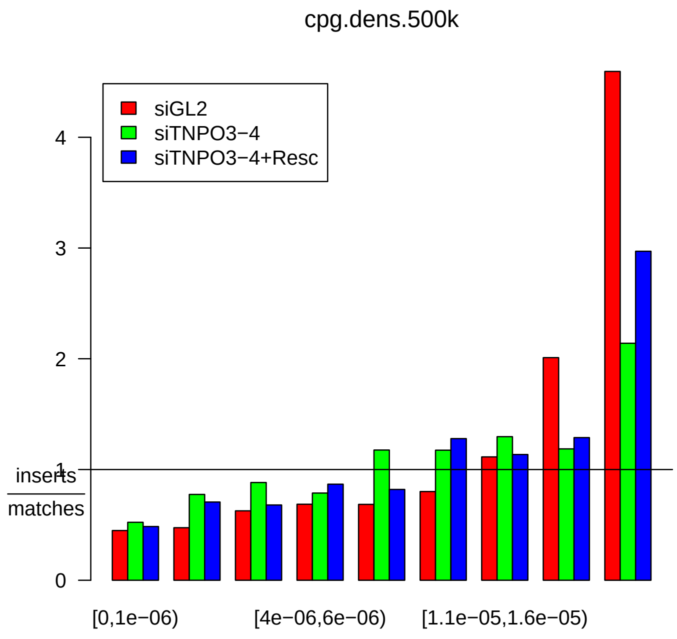

	coef	se	z	p
siGL2	1.070	0.0740	14.5	8.71e-48
siTNPO3-4	0.542	0.1390	3.9	9.67e-05
siTNPO3-4+Resc	0.741	0.0637	11.6	2.72e-31

4.6 1 megabase Window

In the barplot that follows we examine the association of insertion sites with expression density in a 1 megabase window surrounding each locus. First, we count just the number of genes represented on the chip.

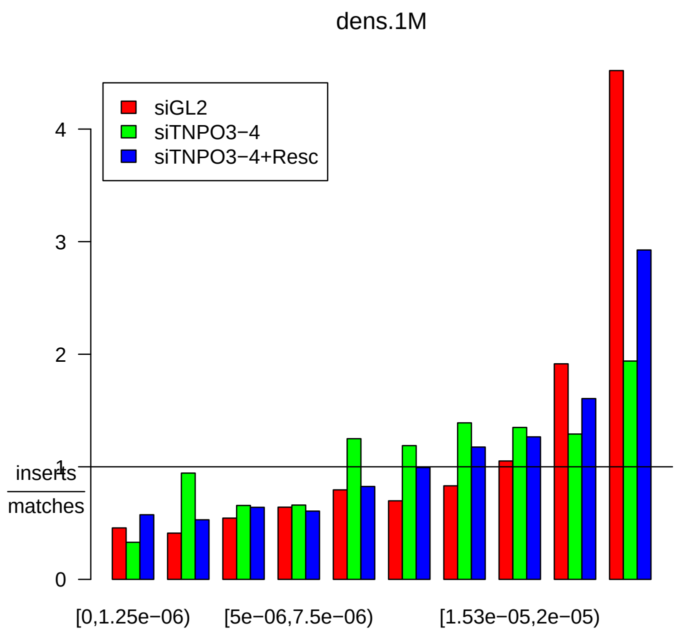

	coef	se	z	p
siGL2	0.966	0.0731	13.2	7.98e-40
siTNPO3-4	0.660	0.1400	4.7	2.57e-06
siTNPO3-4+Resc	0.831	0.0639	13.0	1.11e-38

Here are the results for expression density. First, we count just genes that are in the upper half.

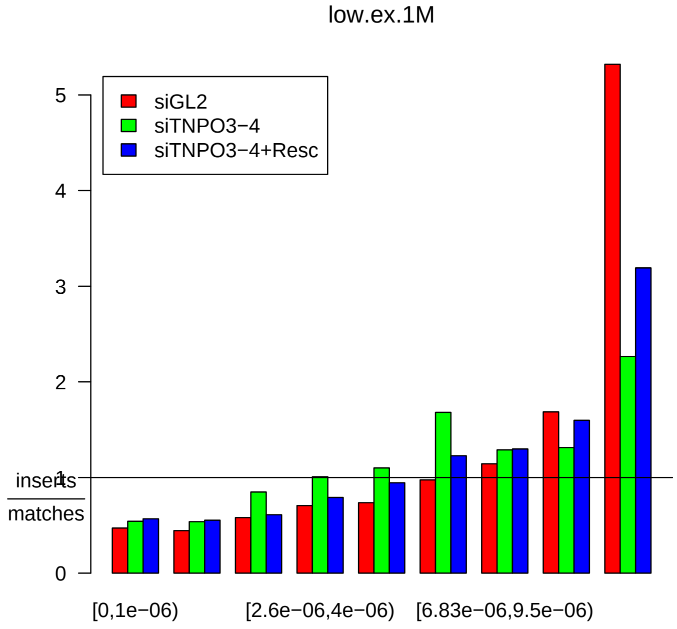

	coef	se	z	p
siGL2	1.100	0.0752	14.60	3.62e-48
siTNPO3-4	0.726	0.1410	5.15	2.56e-07
siTNPO3-4+Resc	0.877	0.0652	13.40	3.38e-41

Now we count genes in the upper $1/8^{th}$:

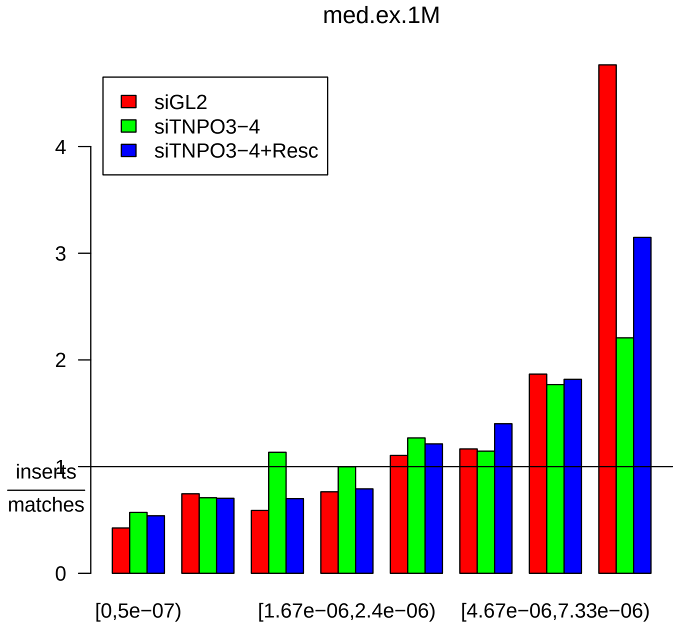

	coef	se	z	p
siGL2	1.160	0.0759	15.30	3.94e-53
siTNPO3-4	0.601	0.1390	4.33	1.46e-05
siTNPO3-4+Resc	0.923	0.0662	13.90	3.39e-44

And here we count genes in the upper $1/16^{th}$:

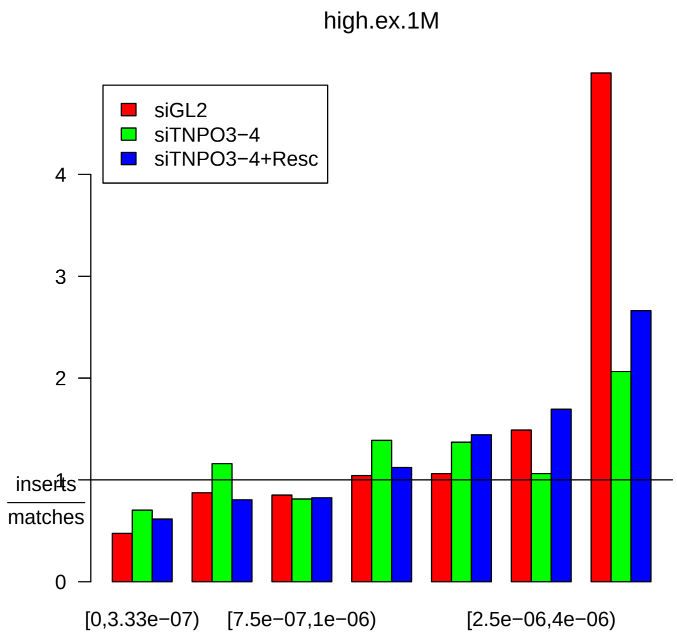

	coef	se	z	p
siGL2	1.060	0.0749	14.20	1.76e-45
siTNPO3-4	0.465	0.1360	3.43	6.04e-04
siTNPO3-4+Resc	0.776	0.0646	12.00	2.98e-33

Here the effect of density of CpG islands is studied:

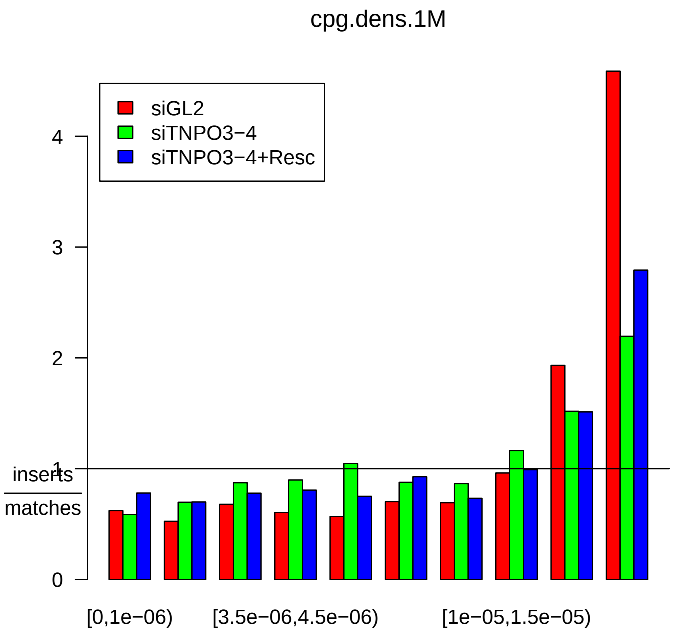

	coef	se	z	p
siGL2	0.910	0.0726	12.50	5.04e-36
siTNPO3-4	0.349	0.1390	2.51	1.20e-02
siTNPO3-4+Resc	0.521	0.0631	8.26	1.44e-16

4.7 2 megabase Window

In the barplot that follows we examine the association of insertion sites with expression density in a 2 megabase window surrounding each locus. First, we count just the number of genes represented on the chip.

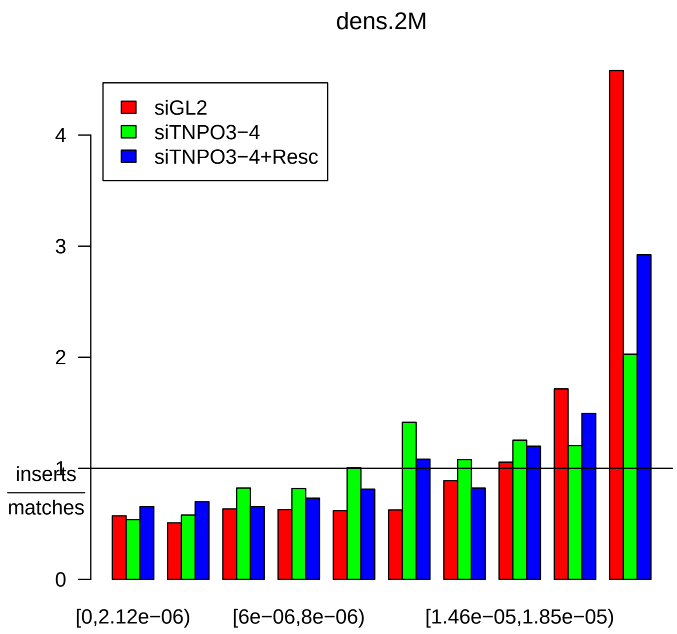

	coef	se	z	p
siGL2	0.944	0.0745	12.70	7.25e-37
siTNPO3-4	0.601	0.1420	4.22	2.42e-05
siTNPO3-4+Resc	0.646	0.0638	10.10	4.37e-24

Here are the results for expression density. First, we count just genes that are in the upper half.

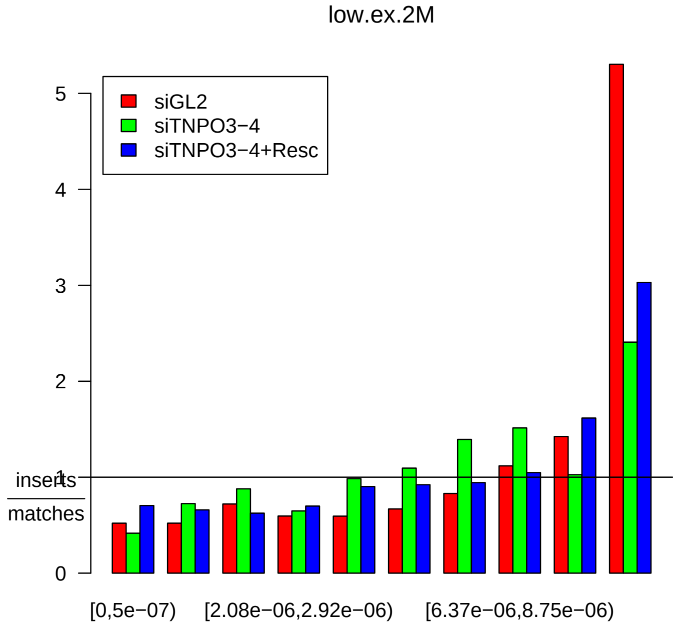

	coef	se	z	p
siGL2	0.955	0.0745	12.80	1.09e-37
siTNPO3-4	0.653	0.1410	4.63	3.66e-06
siTNPO3-4+Resc	0.620	0.0637	9.73	2.20e-22

Now we count genes in the upper $1/8^{th}$:

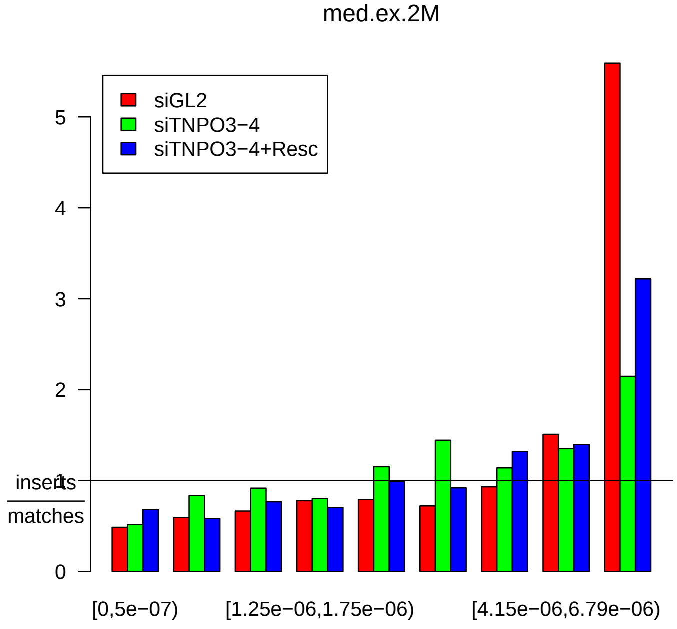

	coef	se	z	p
siGL2	0.915	0.0743	12.30	7.27e-35
siTNPO3-4	0.638	0.1400	4.55	5.24e-06
siTNPO3-4+Resc	0.729	0.0642	11.40	6.48e-30

And here we count genes in the upper $1/16^{th}$:

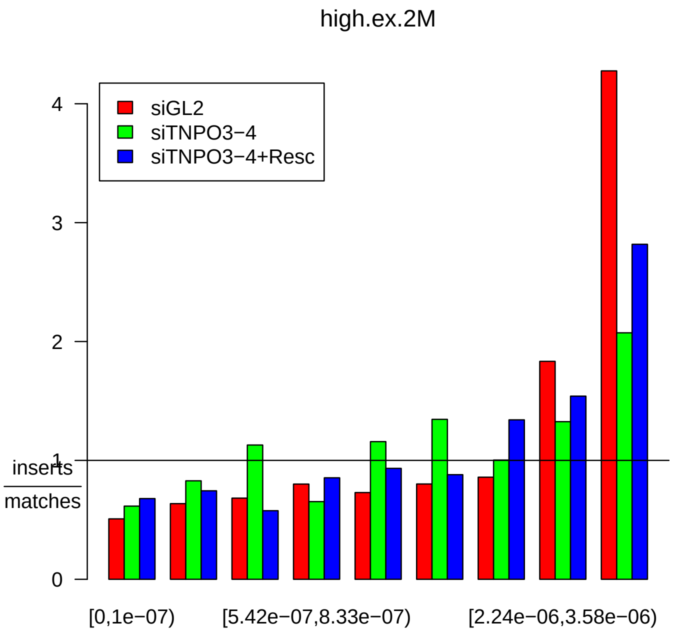

	coef	se	z	p
siGL2	0.861	0.0740	11.60	3.05e-31
siTNPO3-4	0.490	0.1370	3.59	3.33e-04
siTNPO3-4+Resc	0.642	0.0642	10.00	1.49e-23

Here the effect of density of CpG islands is studied:

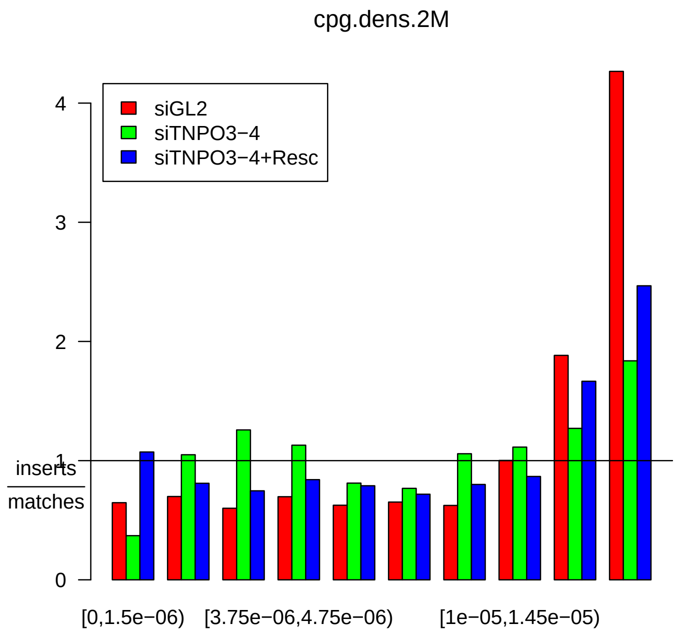

	coef	se	z	p
siGL2	0.784	0.0727	10.80	4.21e-27
siTNPO3-4	0.217	0.1360	1.60	1.10e-01
siTNPO3-4+Resc	0.335	0.0631	5.31	1.09e-07

4.8 4 megabase Window

In the barplot that follows we examine the association of insertion sites with expression density in a 4 megabase window surrounding each locus. First, we count just the number of genes represented on the chip.

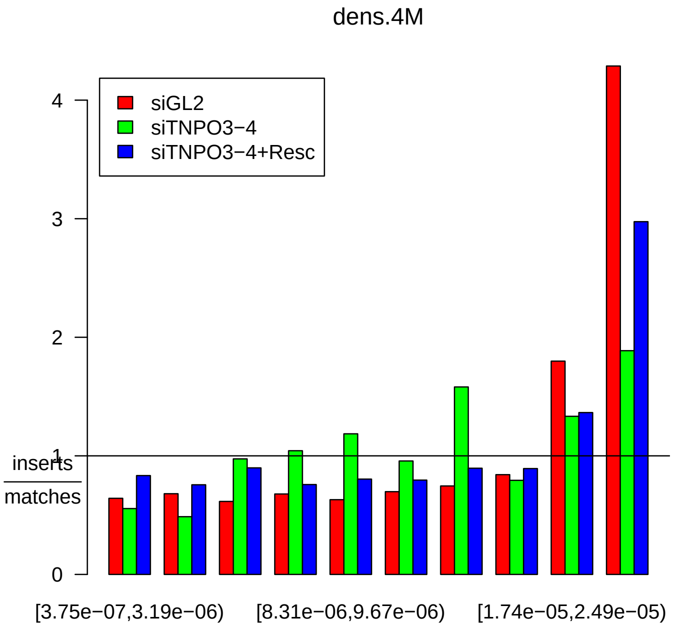

	coef	se	z	p
siGL2	0.780	0.0730	10.70	1.24e-26
siTNPO3-4	0.380	0.1360	2.80	5.13e-03
siTNPO3-4+Resc	0.397	0.0629	6.31	2.79e-10

Here are the results for expression density. First, we count just genes that are in the upper half.

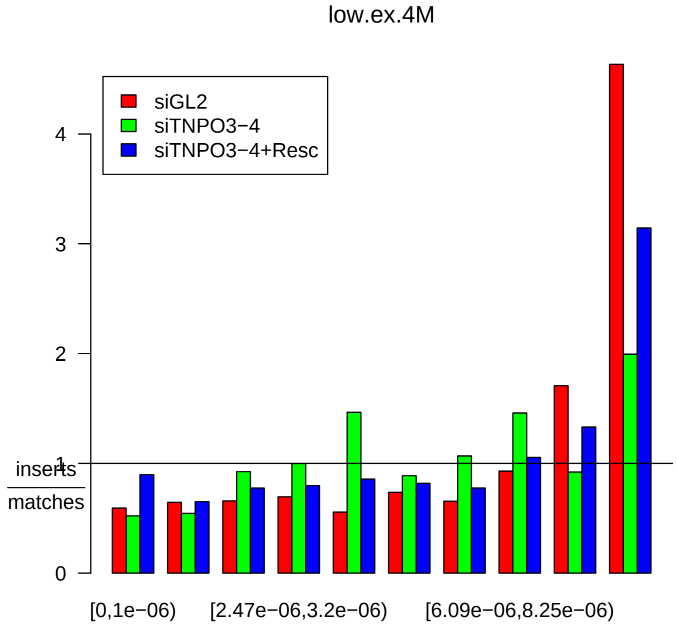

	coef	se	z	p
siGL2	0.841	0.0736	11.40	2.68e-30
siTNPO3-4	0.317	0.1380	2.29	2.19e-02
siTNPO3-4+Resc	0.424	0.0626	6.77	1.33e-11

Now we count genes in the upper $1/8^{th}$:

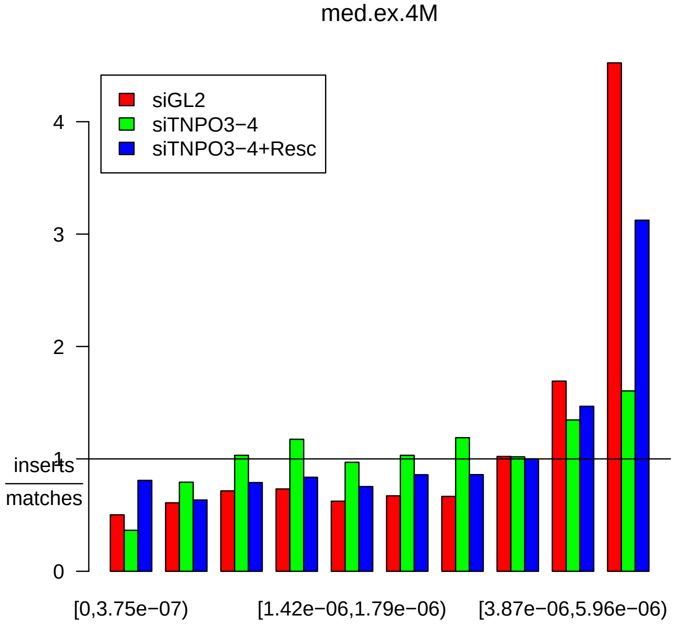

	coef	se	z	p
siGL2	0.838	0.0740	11.30	1.06e-29
siTNPO3-4	0.336	0.1330	2.52	1.18e-02
siTNPO3-4+Resc	0.502	0.0634	7.92	2.29e-15

And here we count genes in the upper $1/16^{th}$:

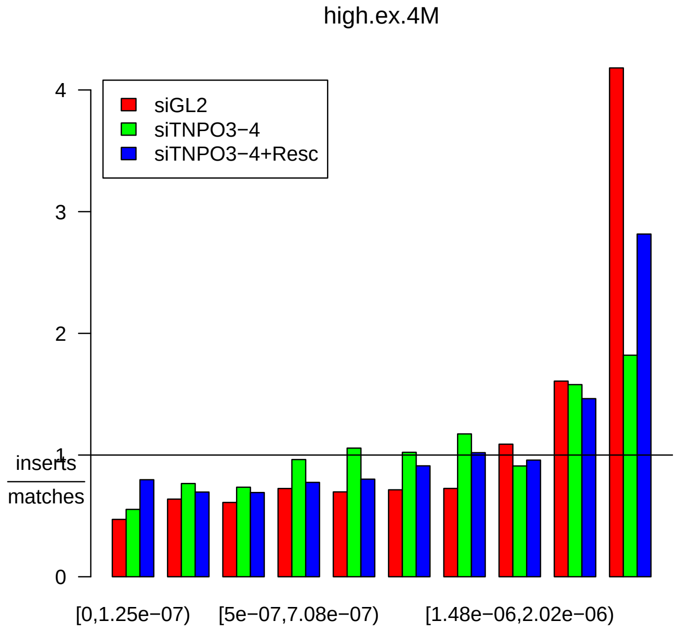

	coef	se	z	p
siGL2	0.858	0.0740	11.60	4.13e-31
siTNPO3-4	0.394	0.1340	2.95	3.21e-03
siTNPO3-4+Resc	0.530	0.0634	8.36	6.39e-17

Here the effect of density of CpG islands is studied:

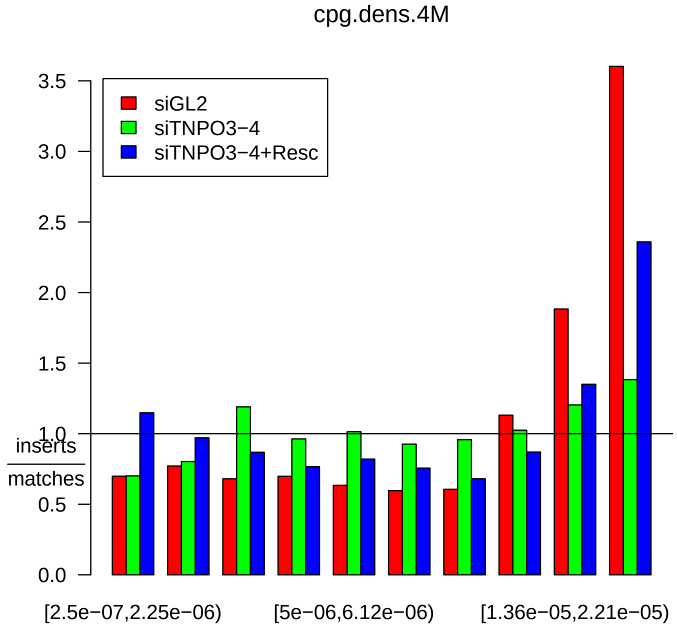

	coef	se	z	p
siGL2	0.663	0.0719	9.230	2.71e-20
siTNPO3-4	0.101	0.1360	0.745	4.56e-01
siTNPO3-4+Resc	0.180	0.0622	2.900	3.75e-03

4.9 8 megabase Window

In the barplot that follows we examine the association of insertion sites with expression density in a 8 megabase window surrounding each locus. First, we count just the number of genes represented on the chip.

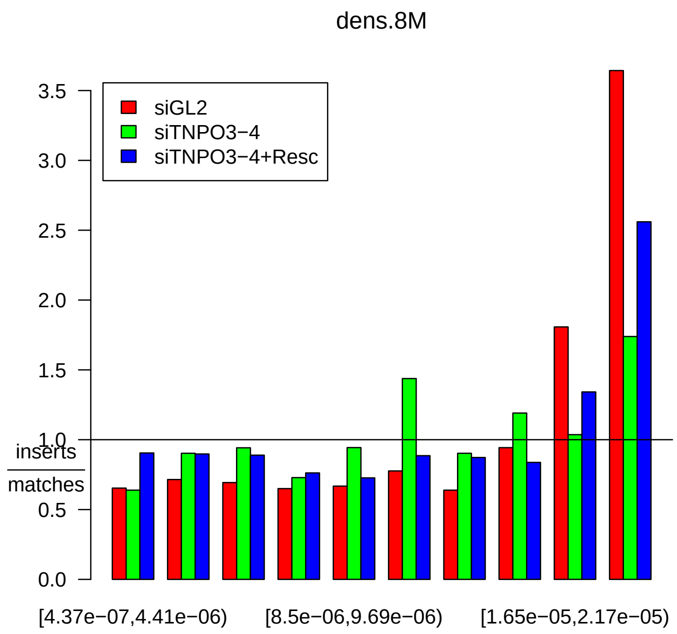

	coef	se	z	p
siGL2	0.729	0.0724	10.10	7.33e-24
siTNPO3-4	0.388	0.1370	2.83	4.62e-03
siTNPO3-4+Resc	0.339	0.0624	5.43	5.58e-08

Here are the results for expression density. First, we count just genes that are in the upper half.

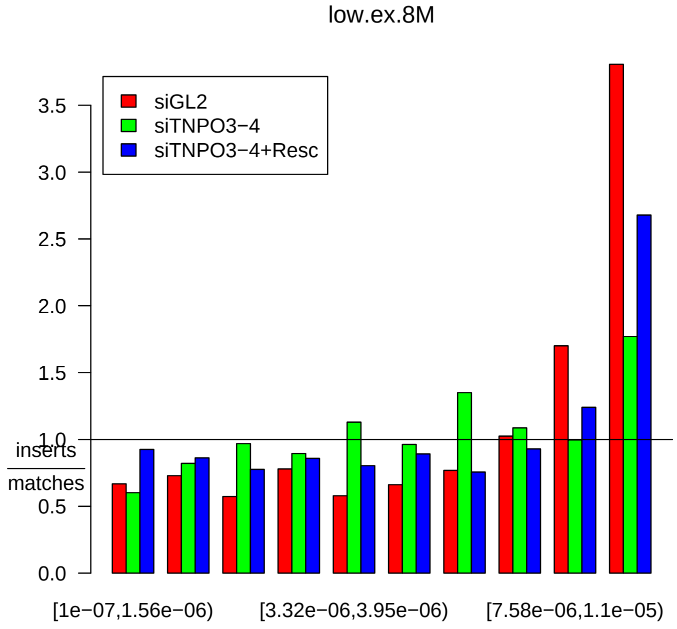

	coef	se	z	p
siGL2	0.738	0.0724	10.20	2.21e-24
siTNPO3-4	0.286	0.1380	2.08	3.74e-02
siTNPO3-4+Resc	0.312	0.0622	5.02	5.26e-07

Now we count genes in the upper $1/8^{th}$:

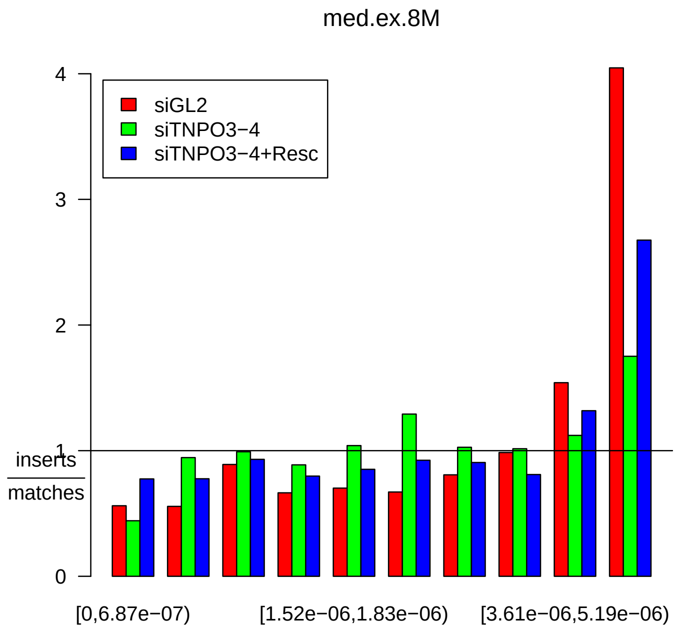

	coef	se	z	p
siGL2	0.749	0.0728	10.30	8.11e-25
siTNPO3-4	0.333	0.1340	2.48	1.31e-02
siTNPO3-4+Resc	0.357	0.0630	5.66	1.52e-08

And here we count genes in the upper $1/16^{th}$:

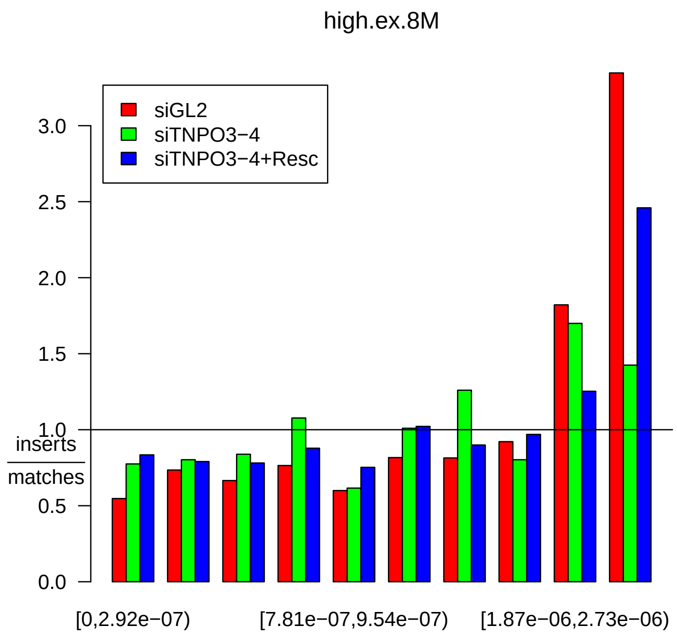

	coef	se	z	p
siGL2	0.773	0.0725	10.70	1.71e-26
siTNPO3-4	0.400	0.1350	2.96	3.10e-03
siTNPO3-4+Resc	0.410	0.0629	6.52	7.02e-11

Here the effect of density of CpG islands is studied:

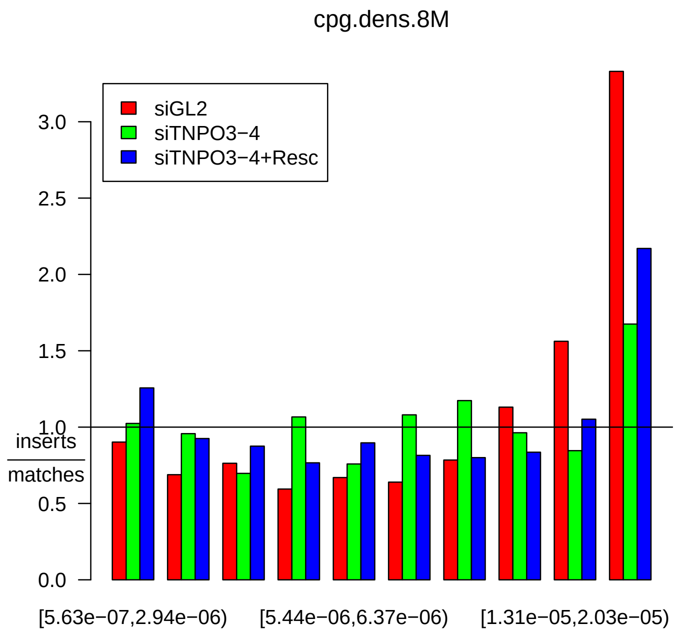

	coef	se	z	p
siGL2	0.625	0.0714	8.75	2.17e-18
siTNPO3-4	0.188	0.1360	1.38	1.67e-01
siTNPO3-4+Resc	0.128	0.0624	2.06	3.94e-02

4.10 16 megabase Window

In the barplot that follows we examine the association of insertion sites with expression density in a 16 megabase window surrounding each locus. First, we count just the number of genes represented on the chip.

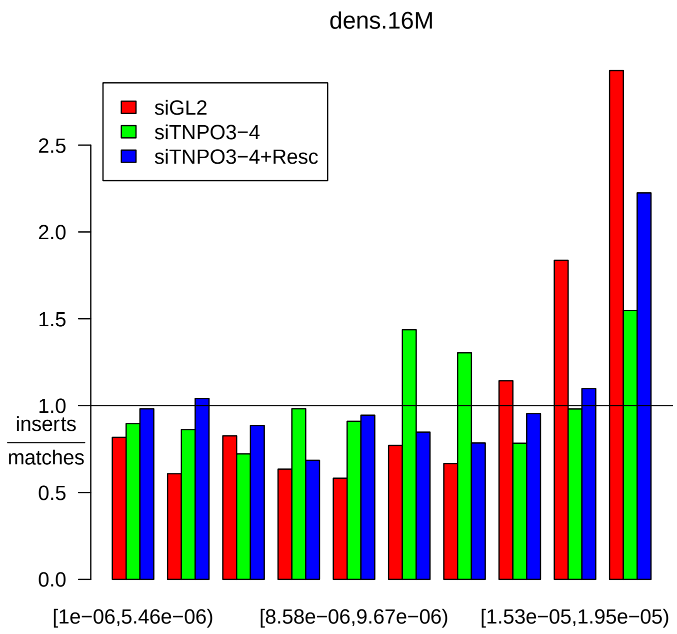

	coef	se	z	p
siGL2	0.683	0.0723	9.45	3.33e-21
siTNPO3-4	0.265	0.1370	1.93	5.33e-02
siTNPO3-4+Resc	0.190	0.0624	3.04	2.37e-03

Here are the results for expression density. First, we count just genes that are in the upper half.

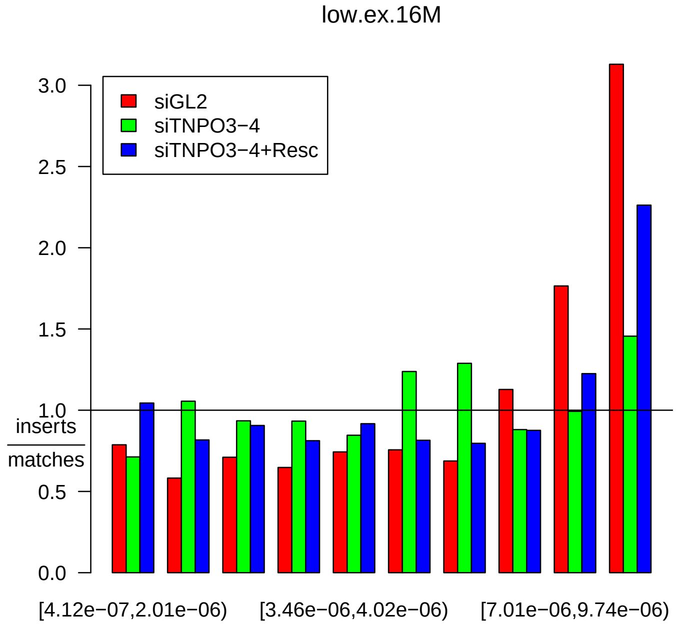

	coef	se	z	p
siGL2	0.683	0.072	9.49	2.41e-21
siTNPO3-4	0.247	0.137	1.80	7.13e-02
siTNPO3-4+Resc	0.208	0.063	3.30	9.67e-04

Now we count genes in the upper $1/8^{th}$:

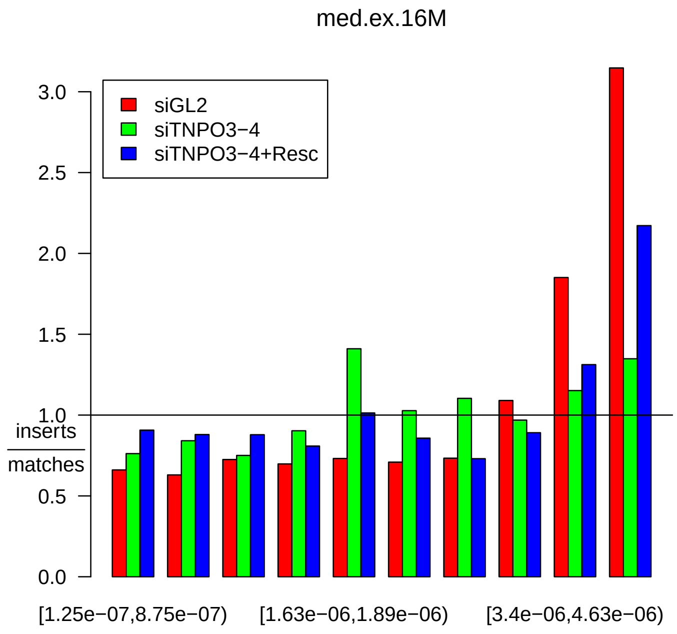

	coef	se	z	p
siGL2	0.687	0.0716	9.60	8.32e-22
siTNPO3-4	0.181	0.1350	1.34	1.81e-01
siTNPO3-4+Resc	0.197	0.0625	3.15	1.66e-03

And here we count genes in the upper $1/16^{th}$:

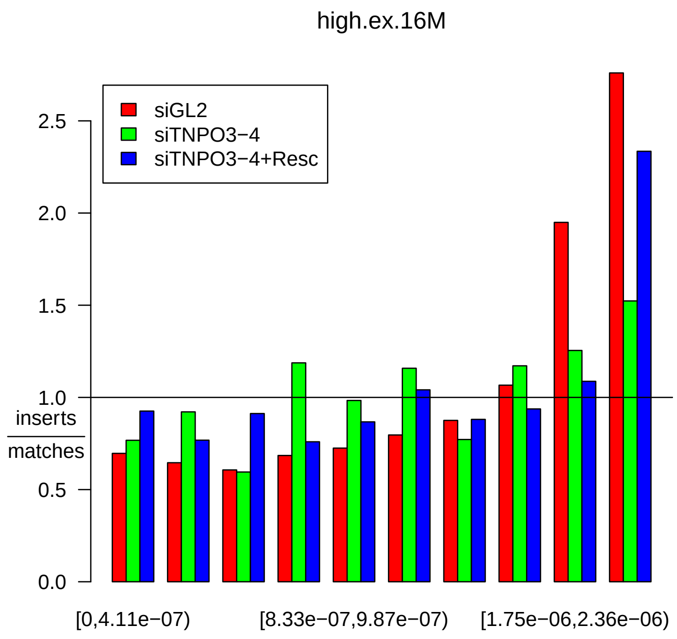

	coef	se	z	p
siGL2	0.722	0.0713	10.10	4.15e-24
siTNPO3-4	0.267	0.1340	1.99	4.70e-02
siTNPO3-4+Resc	0.314	0.0627	5.01	5.52e-07

Here the effect of density of CpG islands is studied:

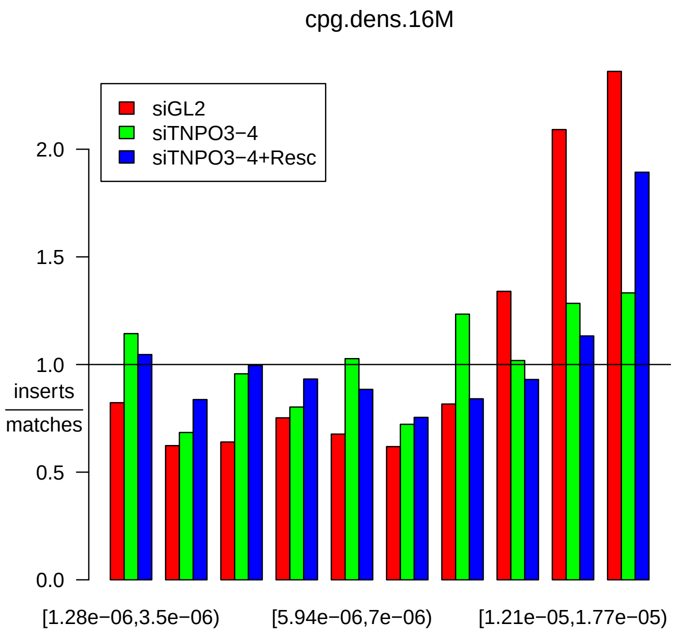

	coef	se	z	p
siGL2	0.654	0.0716	9.13	7.14e-20
siTNPO3-4	0.171	0.1350	1.26	2.08e-01
siTNPO3-4+Resc	0.116	0.0621	1.86	6.28e-02

4.11 32 megabase Window

In the barplot that follows we examine the association of insertion sites with expression density in a 32 megabase window surrounding each locus. First, we count just the number of genes represented on the chip.

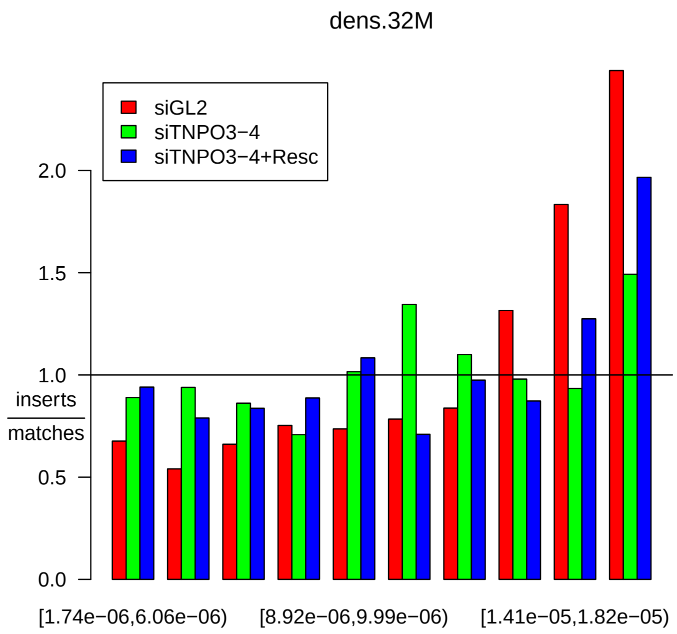

	coef	se	z	p
siGL2	0.747	0.0727	10.30	8.85e-25
siTNPO3-4	0.257	0.1370	1.88	6.00e-02
siTNPO3-4+Resc	0.184	0.0624	2.94	3.26e-03

Here are the results for expression density. First, we count just genes that are in the upper half.

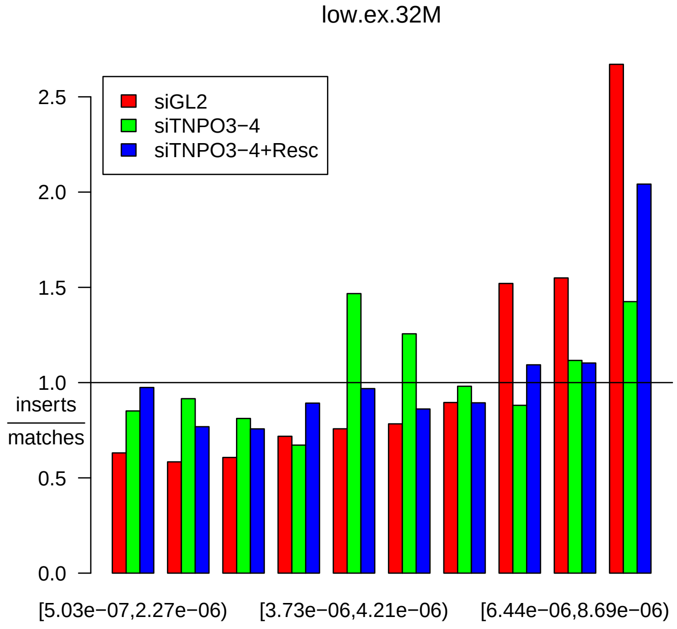

	coef	se	z	p
siGL2	0.778	0.0730	10.70	1.68e-26
siTNPO3-4	0.180	0.1360	1.32	1.86e-01
siTNPO3-4+Resc	0.267	0.0627	4.27	1.98e-05

Now we count genes in the upper $1/8^{th}$:

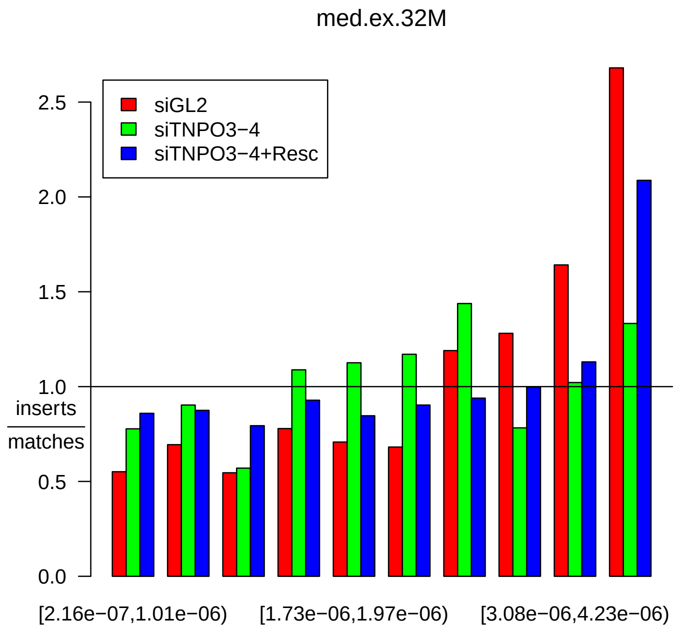

	coef	se	z	p
siGL2	0.775	0.0720	10.80	5.32e-27
siTNPO3-4	0.237	0.1360	1.74	8.12e-02
siTNPO3-4+Resc	0.295	0.0627	4.70	2.64e-06

And here we count genes in the upper $1/16^{th}$:

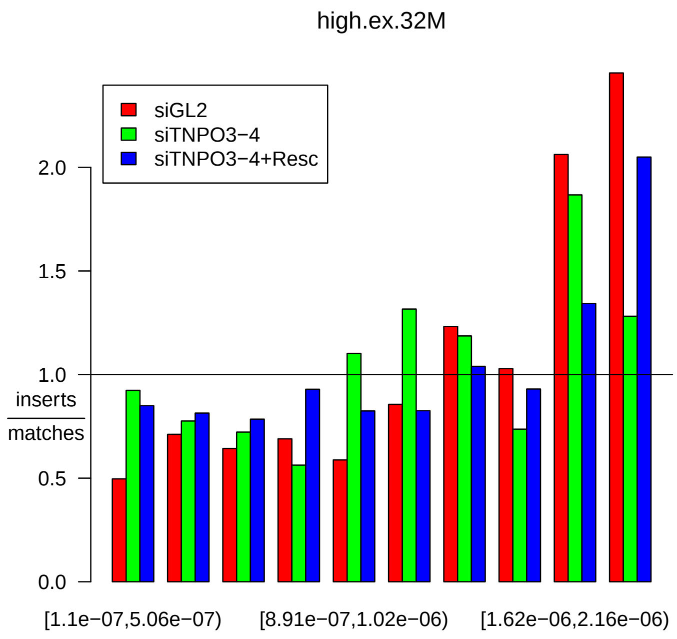

	coef	se	z	p
siGL2	0.859	0.0730	11.80	6.15e-32
siTNPO3-4	0.388	0.1370	2.83	4.59e-03
siTNPO3-4+Resc	0.332	0.0626	5.30	1.13e-07

Here the effect of density of CpG islands is studied:

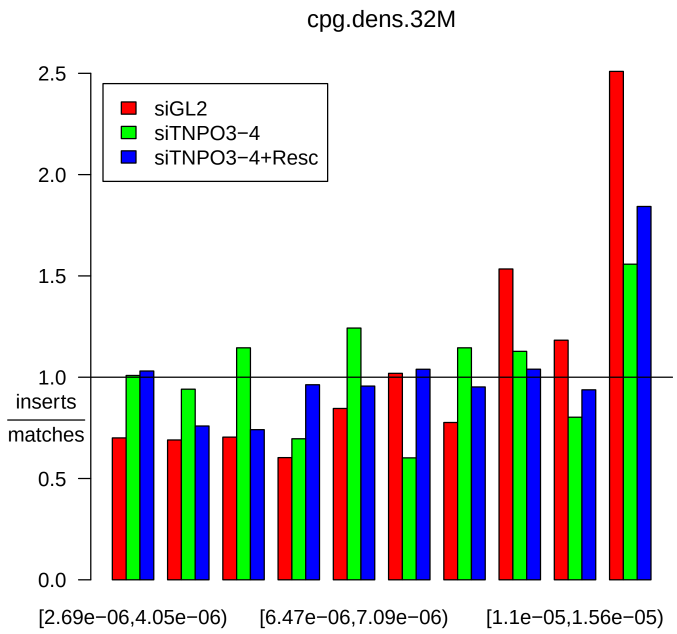

	coef	se	z	p
siGL2	0.63900	0.0711	8.9900	2.43e-19
siTNPO3-4	0.00622	0.1370	0.0455	9.64e-01
siTNPO3-4+Resc	0.21400	0.0615	3.4800	4.98e-04

5 Juxtaposition with Gene Start and End Positions

5.1 Acembly Annotations

In this section we study the effect of juxtaposition in terms of gene start and end positions. The first barplot shows the effect of gene width for those insertions that are located within an Acembly gene. The table following the barplot shows the p-values for a test of the hypothesis that the proportions in each of the categories that define the bars are equal in the insertions and their matches. This p-value is obtained from the $5 \times 2 \times k$ table of counts defined by gene width category, insertion/match status, and stratum (consisting of an insertion and its matched sites) using a likelihood ratio test for the hypothesis of no association between gene width category and insertion/match status. The test used compared the log-linear model [1] with all two-way configurations to that with no gene width category and insertion/match status configuration.

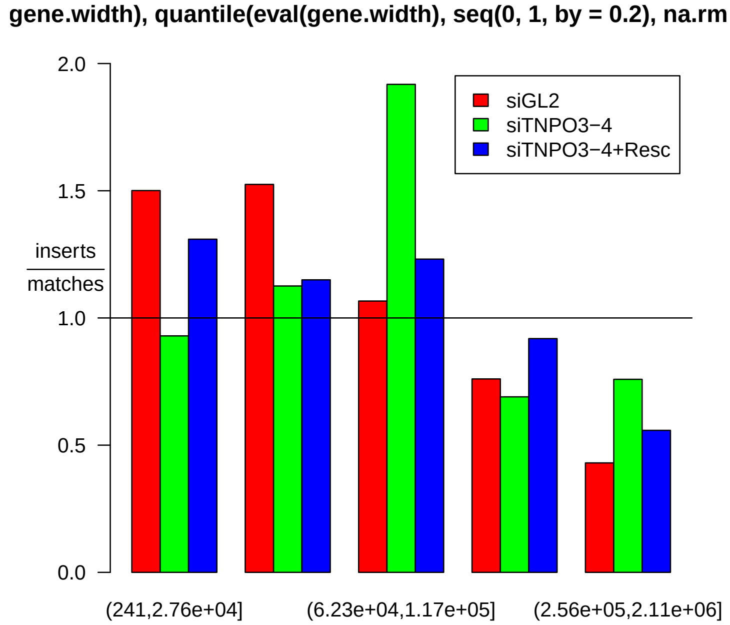


```
   siGL2      siTNPO3-4 siTNPO3-4+Resc
1.39e-28       2.95e-05       6.84e-15
```

The next plot uses the width of a non-gene region for insertions that fall into such regions.

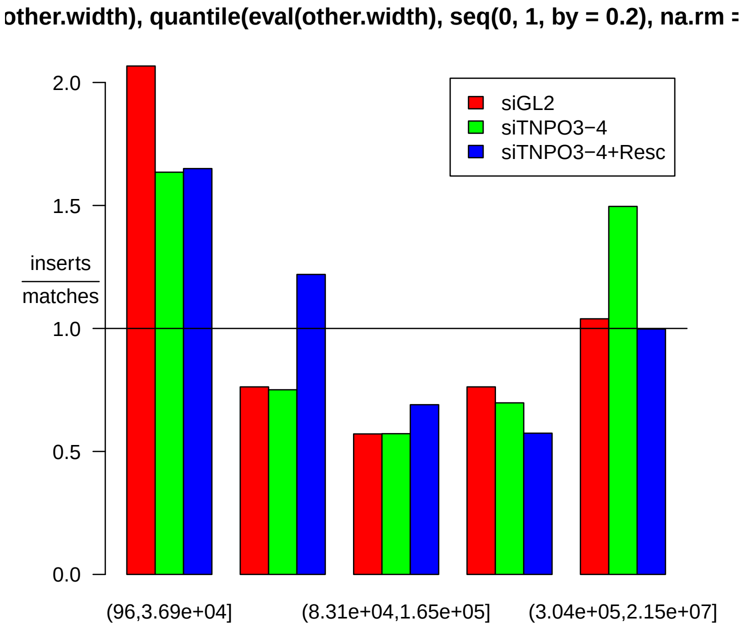


```
    siGL2       siTNPO3-4 siTNPO3-4+Resc
 4.36e-14        1.06e-02       2.07e-06
```

The next plot studies the distance to the nearest boundary between a gene and a non-gene region. The distance is expressed as a fraction of the length of the region. Thus, '0.25' refers to one quarter of the distance from the site to nearest boundary divided by the total width of the region.

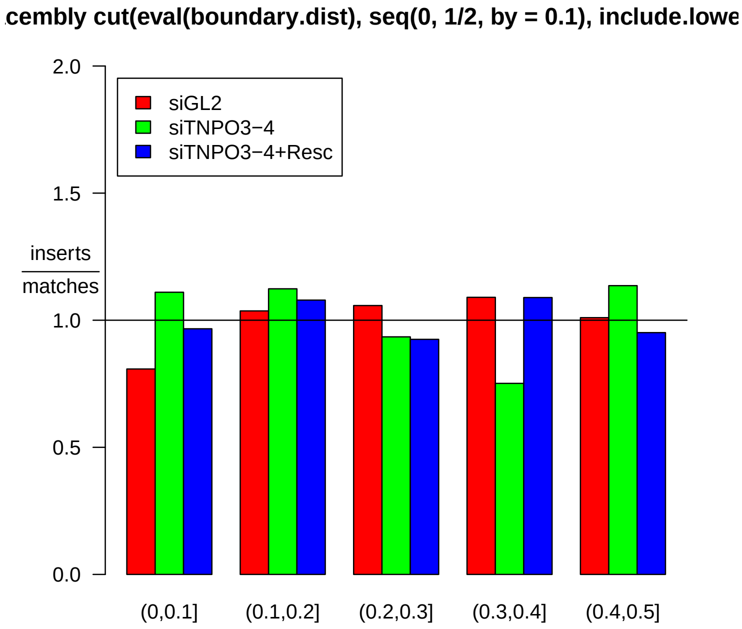

siGL2	siTNPO3-4	siTNPO3-4+Resc
0.0216	0.0556	0.1690

This plot studies the effect of nearness to the beginning of a transcript. For sites in genes, it is the distance to the start of the gene divided by the width of the gene. For other sites it is the distance from the site to the nearer gene if that gene boundary is also a transcription starting point. Locations near '0' are relatively near the beginning of transcription, while those near '1' are near the termination of the transcript.

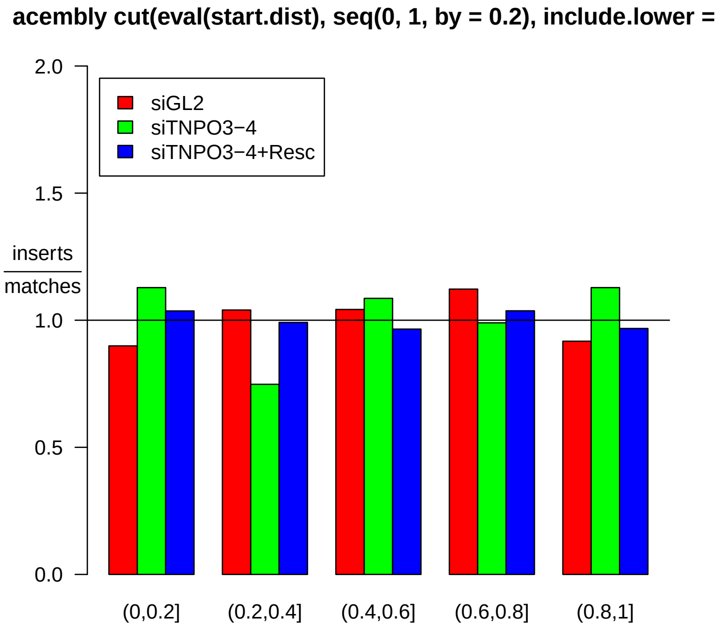

siGL2	siTNPO3-4	siTNPO3-4+Resc
0.1580	0.0841	0.9050

5.2 RefSeq Annotations

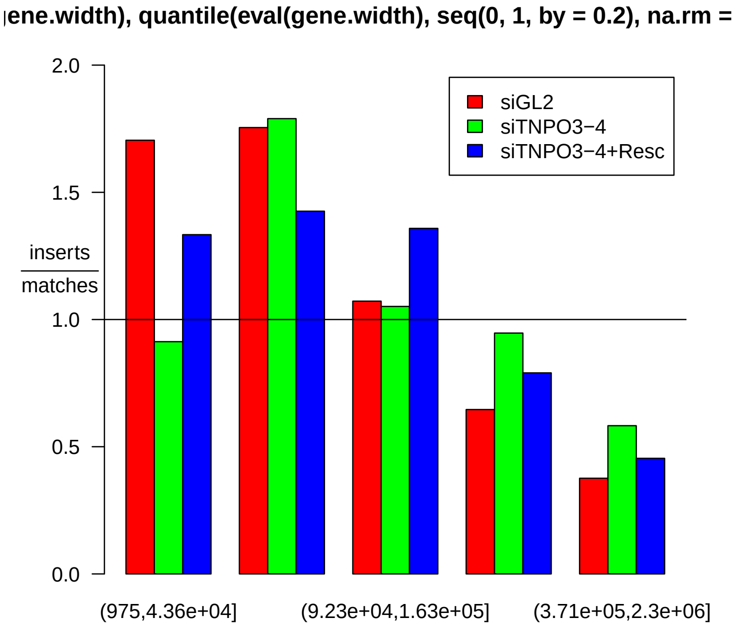


```
     siGL2       siTNPO3-4 siTNPO3-4+Resc
  6.03e-34        2.93e-04       2.93e-26
```

ther.width), quantile(eval(other.width), seq(0, 1, by = 0.2), na.rm =

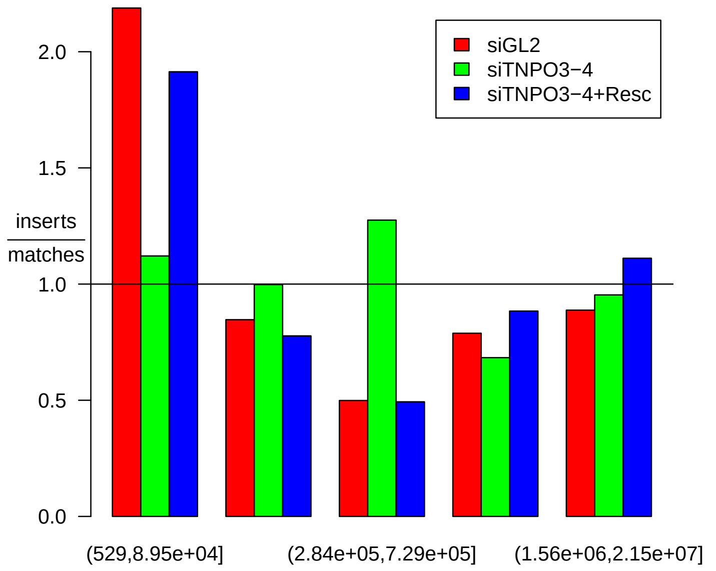


```
    siGL2       siTNPO3-4 siTNPO3-4+Resc
 4.33e-15        4.95e-01       6.93e-14
```

refSeq cut(eval(boundary.dist), seq(0, 1/2, by = 0.1), include.lower

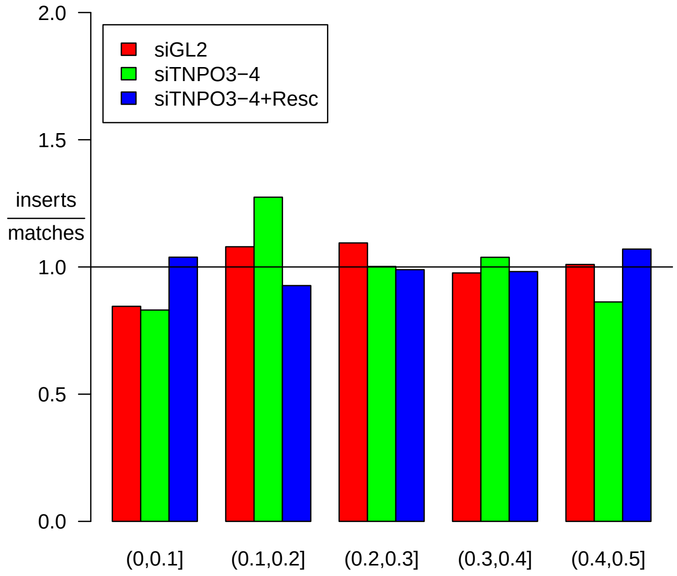

siGL2	siTNPO3-4	siTNPO3-4+Resc
0.0541	0.0971	0.3980

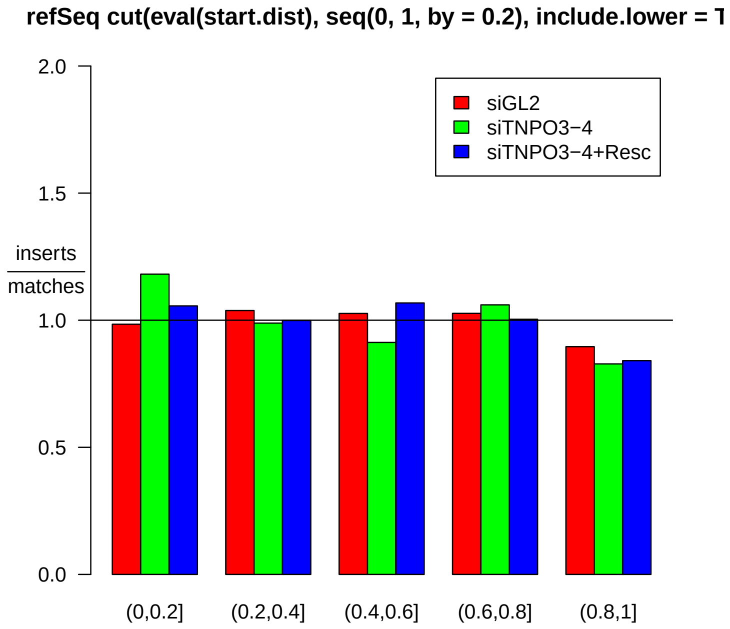


```
 siGL2       siTNPO3-4 siTNPO3-4+Resc
0.7580          0.4380          0.0421
```

5.3 genScan Annotations

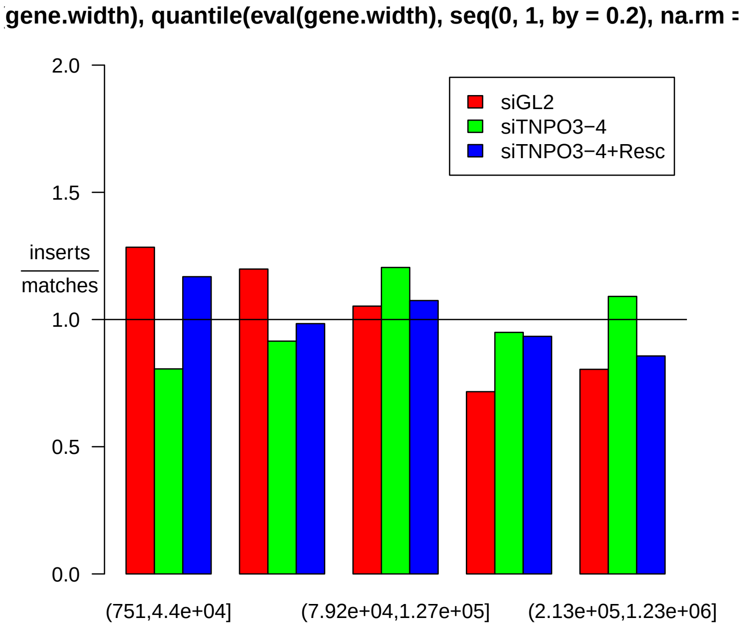


```
   siGL2       siTNPO3-4 siTNPO3-4+Resc
9.84e-08       1.10e-01       9.99e-02
```

other.width), quantile(eval(other.width), seq(0, 1, by = 0.2), na.rm =

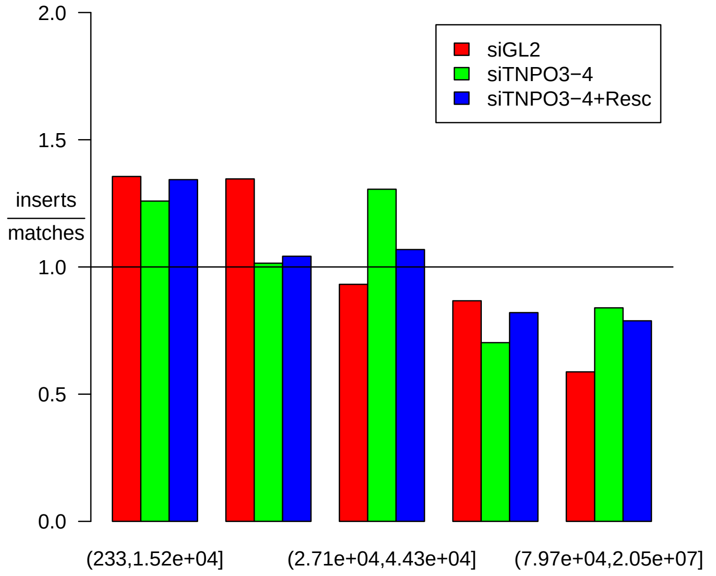


```
    siGL2       siTNPO3-4 siTNPO3-4+Resc
 1.06e-05        9.89e-02       1.54e-01
```

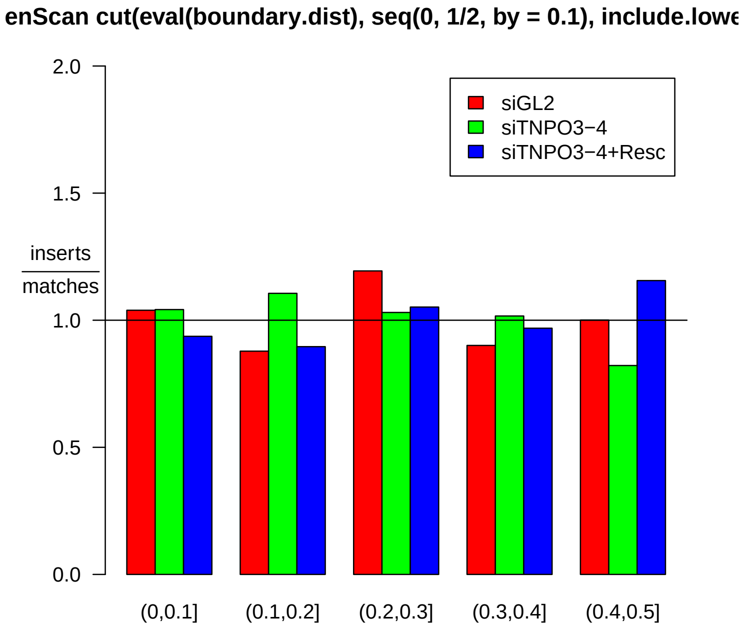


```
  siGL2      siTNPO3-4 siTNPO3-4+Resc
0.00641        0.60000        0.01670
```

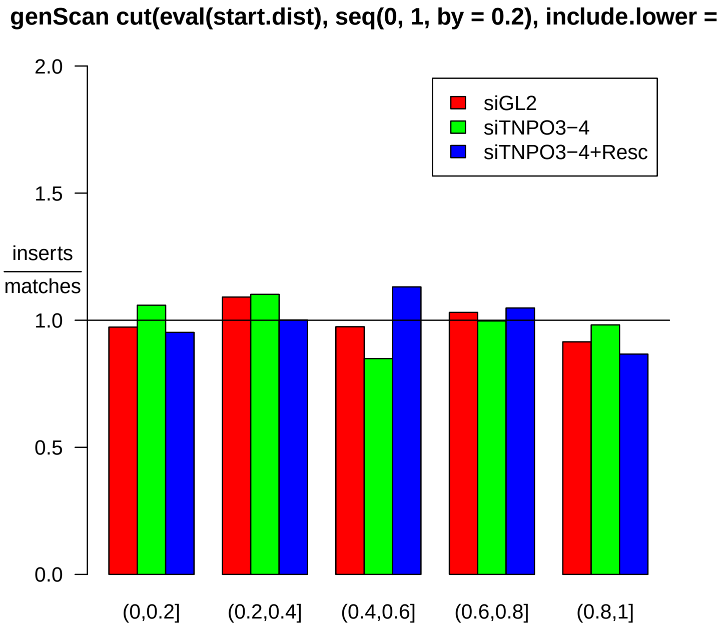

siGL2	siTNPO3-4	siTNPO3-4+Resc
0.204	0.818	0.010

5.4 uniGene Annotations

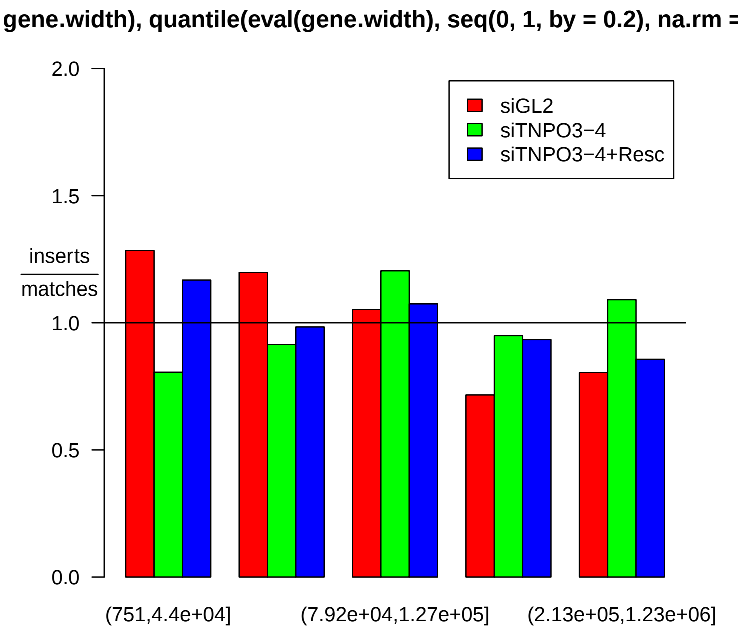


```
   siGL2       siTNPO3-4 siTNPO3-4+Resc
9.84e-08        1.10e-01       9.99e-02
```

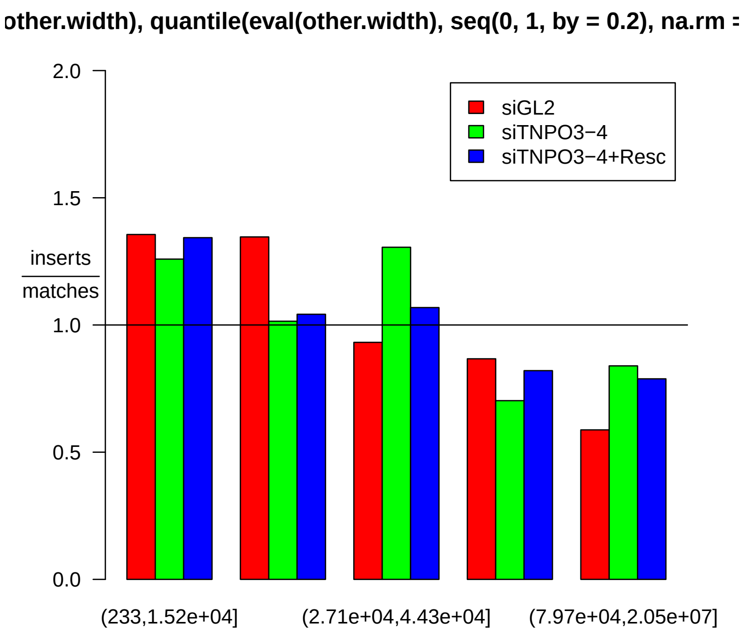


```
   siGL2      siTNPO3-4 siTNPO3-4+Resc
1.06e-05       9.89e-02       1.54e-01
```

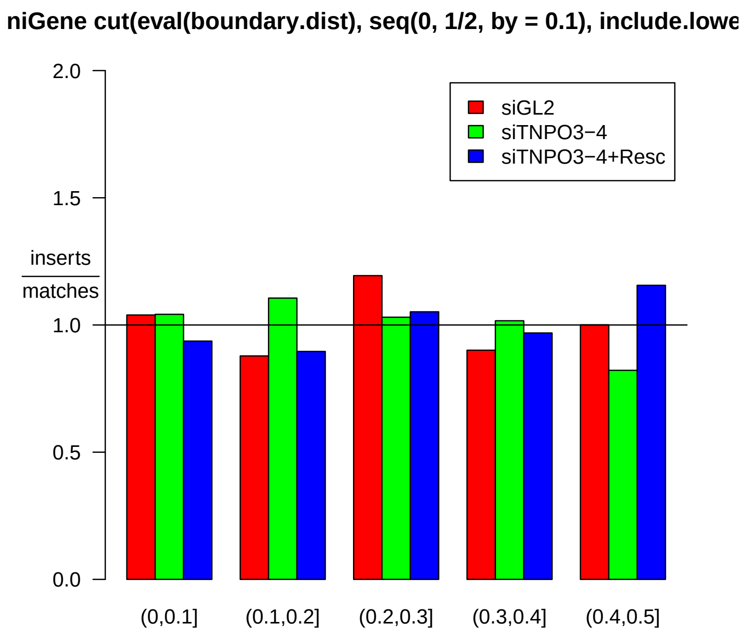

siGL2	siTNPO3-4	siTNPO3-4+Resc
0.00641	0.60000	0.01670

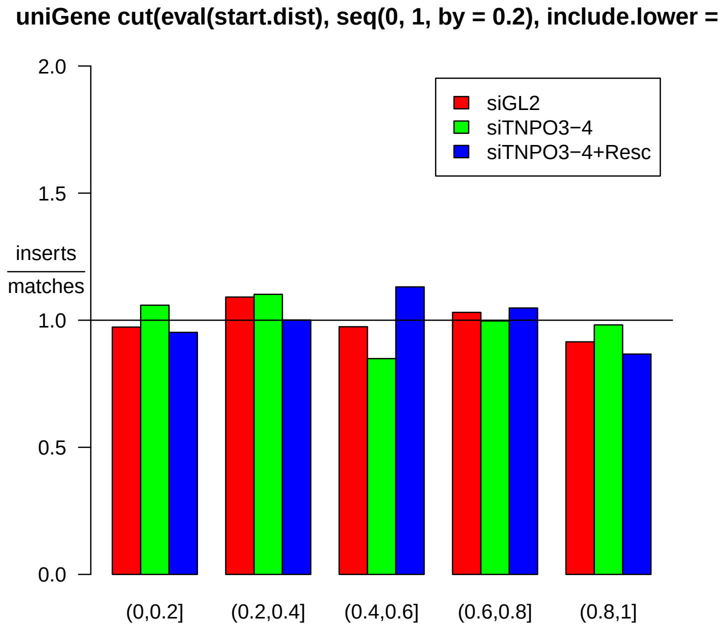

siGL2	siTNPO3-4	siTNPO3-4+Resc
0.204	0.818	0.010

6 GC content

Here we study the effect of GC content on insertion. The GC content is taken from the Human Genome Draft at GoldenPath from the table `http://genome.ucsc.edu/goldenPath/hg18/database/gc5Base.txt.gz`.

Following the plot is a table of fitted coefficients based on splitting the GC percent data at the median.

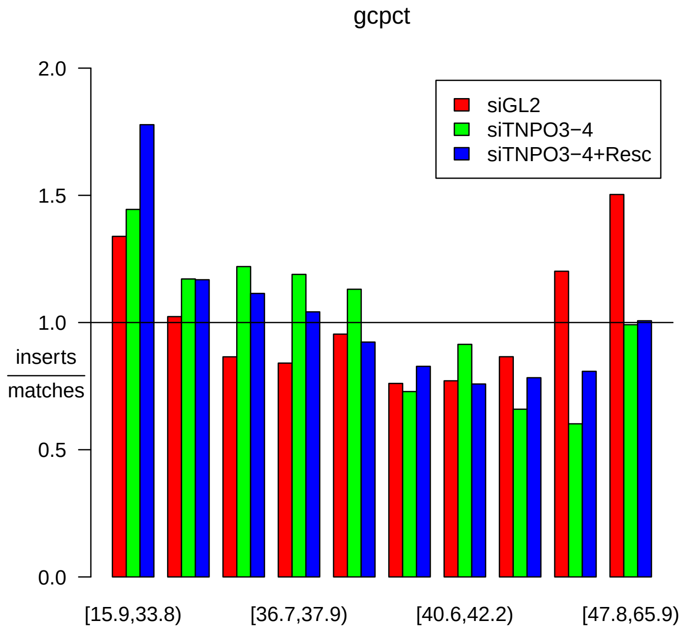

	coef	se	z	p
siGL2	-0.0133	0.0707	-0.188	8.51e-01
siTNPO3-4	-0.5040	0.1380	-3.650	2.64e-04
siTNPO3-4+Resc	-0.3880	0.0629	-6.170	6.88e-10

7 Cytobands

Here we study the association of cytoband with insertion intensity. The data are obtained from
`http://genome.ucsc.edu/goldenPath/hg18/database/cytoBand.txt.gz`.

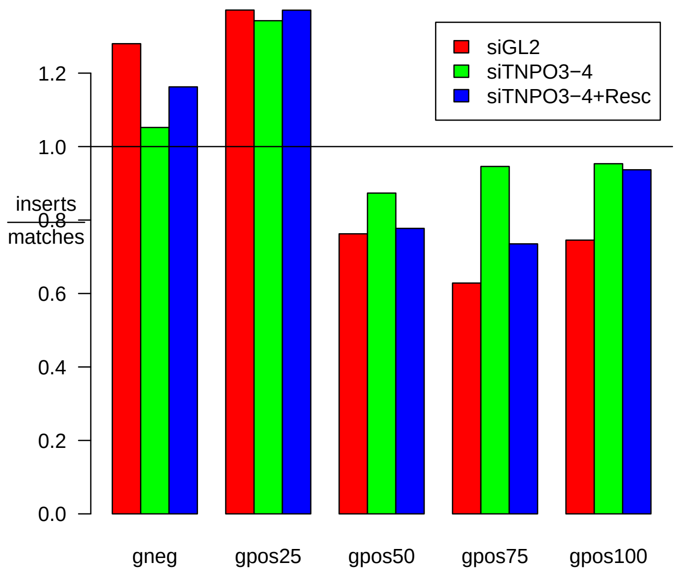

A formal test of significance attains a p-value of $< 2.22e-16$. Here is the table of coefficients of the log ratio of intensities for true insertion sites versus control insertion sites (comparing each category of Giemsa staining to 'gneg') along with their standard errors, z statistics, and p-values:

	coef	se	z	p
cyto.typeacen	NA	0.0000	NA	NA
cyto.typegpos100	-0.323	0.0612	-5.27	1.35e-07
cyto.typegpos25	0.124	0.0839	1.48	1.39e-01
cyto.typegpos50	-0.438	0.0708	-6.18	6.47e-10
cyto.typegpos75	-0.509	0.0703	-7.24	4.43e-13
cyto.typegvar	NA	0.0000	NA	NA

References

[1] Yvonne M.M. Bishop, Stephen E. Fienberg, and Paul W. Holland. *Discrete multivariate analyses: Theory and practice* (MIT Press, 1975).

[2] P. McCullagh and John A. Nelder. *Generalized linear models.* (Chapman & Hall ltd, 1999).

[3] Xiaolin Wu, Yuan Li, Bruce Crise, Shawn M. Burgess "Transcription Start Regions in the Human Genome Are Favored Targets for MLV Integration," *Science,* **300**(5626), (June 2003): 1749-1751.
