# Supplementary material for: HIV Integration Targeting: A Pathway Involving Transportin-3 and the Nuclear Pore Protein RanBP2
Source: PLoS Pathog. 2011 Mar 10;7(3):e1001313. doi: 10.1371/journal.ppat.1001313 (PMC3053352; doi:10.1371/journal.ppat.1001313)
Supplement: Text S3 — Integration site preference under gene silencing. (1.33 MB PDF) [file ppat.1001313.s013.pdf]

Integration Site Preference Under Gene Silencing

Charles C. Berry

July 9, 2009

1 Introduction

It is of interest to see how the action of different genes affects the integration site preference of a retrovirus. One tool for assessing the effects of a gene is the use of an siRNA which inteferes with gene transcription. In this analysis the integration site profiles of a number of knockdown lines are studied and compared.

Several strategies re used. One is to comapre the distribution of a variable or collection of variables using measures of location. In the simplest case, the proportions of integration sites in genes are compared. For collections of related variables, the Mahalanobis distance between sets of means is used. In that case the variance-covariance matrix used is based on the pooled central moments over all lines, i.e. the mean of each line is subtracted from the values for that line and the sums of squares and crossproducts of the centered variables for each line are aggregated.

Yet another strategy used is to fit a model to data that can be used to predict integration intensity [Berry et al., 2006] from each such knockdown line. The predictive model is based on genomic features of the integration sites (and those of matched random controls), such as local nucleotide sequence, location in a gene, GC proportion of surrounding regions, et cetera. Using the collection of models from a collection of siRNA knockdowns, one can predict the integration intensity over the host genome and identify pairs of knockdowns that differ from one another in terms of retroviral preference for particular sites on the host genome and quantify this difference.

In order to form the collection of models, a statistical learning tool (or tools) is (are) needed that will provide predictions notwithstanding numerical issues such as rank insufficiency amongst candidate regressors. Moreover, the statistical tools ought to make sensible compromises between bias and variance, that is, they ought to avoid overfitting wihtout being excessively rigid.

Two methods are used here. One uses a penalized conditional logit model (i.e. conditional logit ridge regression). This is implemented in the R `survival` package. The other uses conditional logit BMA, which can be obtained from a modified version of the `bic.surv` function in the R `BMA` package.

In each of these methods, computational issues may arise due to rank insufficiency in the Fisher information or linear separability of integration sites

from their matched controls. These are resolved by using a forward screening of candidate regressors. The ordering of variables in the screening is based on an L1 penalized logistic regression, which implementation is pretty much immune to the numerical issues just mentioned.

2 Data Used

The data used consist of varying number of integration sites from different lines. Here are the counts:

```
                                         sites
Brady-HIV-293T-siANAPC-5-highMOI-Oct08     307
Brady-HIV-293T-siANAPC-5-lowMOI-Oct08      321
Brady-HIV-293T-siANAPC-6-highMOI-Oct08     126
Brady-HIV-293T-siANAPC-8-highMOI-Oct08     205
Brady-HIV-293T-siANAPC-8-lowMOI-Oct08      112
Brady-HIV-293T-siIK-9-highMOI-Oct08        648
Brady-HIV-293T-siIK-9-lowMOI-Oct08         384
Brady-HIV-293T-siMAP4-4-highMOI-Oct08      452
Brady-HIV-293T-siMAP4-4-lowMOI-Oct08        40
Brady-HIV-293T-siMAP4-9-highMOI-Oct08      424
Brady-HIV-293T-siMAP4-9-lowMOI-Oct08       652
Brady-HIV-293T-siMOCK-6-highMOI-Oct08      274
Brady-HIV-293T-siMOCK-lowMOI-Oct08          43
Brady-HIV-293T-siNUP98-3-highMOI-Oct08     132
Brady-HIV-293T-siNUP98-3-lowMOI-Oct08       98
Brady-HIV-293T-siNUP98-5-highMOI-Oct08     175
Brady-HIV-293T-siNUP98-5-lowMOI-Oct08      502
Brady-HIV-293T-siNUP98-7-highMOI-Oct08     128
Brady-HIV-293T-siNUP98-7-lowMOI-Oct08       78
Brady-HIV-293T-siNUP98-8-highMOI-Oct08      87
Brady-HIV-293T-siNUP98-8-lowMOI-Oct08      104
Brady-HIV-293T-siRANBP-6-highMOI-Oct08     463
Brady-HIV-293T-siRANBP-6-lowMOI-Oct08      354
Brady-HIV-293T-siRANBP-Pool-highMOI-Oct08  480
Brady-HIV-293T-siRANBP-Pool-lowMOI-Oct08   366
Brady-HIV-293T-siSKIIP-5-highMOI-Oct08     159
Brady-HIV-293T-siSKIIP-5-lowMOI-Oct08      538
Brady-HIV-293T-siTNPO3-1-highMOI-Oct08      80
Brady-HIV-293T-siTNPO3-1-lowMOI-Oct08       58
Brady-HIV-293T-siTNPO3-2-highMOI-Oct08     168
Brady-HIV-293T-siTNPO3-2-lowMOI-Oct08      514
Brady-HIV-293T-siTNPO3-4-highMOI-Oct08     177
Brady-HIV-293T-siTNPO3-4-lowMOI-Oct08      268
Brady-HIV-293T-siTNPO3-5-highMOI-Oct08     612
Brady-HIV-293T-siTNPO3-5-lowMOI-Oct08      308
```

Brady-HIV-293T-siTNPO3-Pool-highMOI-Oct08	191
Brady-HIV-293T-siTNPO3-Pool-lowMOI-Oct08	222
Brady-HIV-293T-siWDHD1-4-highMOI-Oct08	243
Brady-HIV-293T-siWDHD1-4-lowMOI-Oct08	8
Brady-HIV-293T-siWDR46-1-highMOI-Oct08	182
Brady-HIV-293T-siWDR46-1-lowMOI-Oct08	229
Brady-HIV-293T-siWDR46-Pool-highMOI-Oct08	262
Brady-HIV-293T-siWDR46-Pool-lowMOI-Oct08	296
Ronen-HIV-293T-FLJ_2	1593
Ronen-HIV-293T-FLJ_3	722
Ronen-HIV-293T-GL2	4327
Ronen-HIV-293T-HYPB_1	2273
Ronen-HIV-293T-MAP4_3	3737
Ronen-HIV-293T-MAP4_4	3420
Ronen-HIV-293TsiLL-untreated	468

The numbers of sites range from 8 to 4327. Obviously, it will be difficult to make inferences on lines for which only a few integration sites were recovered.

3 Gene Effects

The following plot shows the fractions of sites located in RefSeq genes. The vertical lines depict the 95 percent confidence intervals. The horizontal line shows the overall proportion of sites in genes.

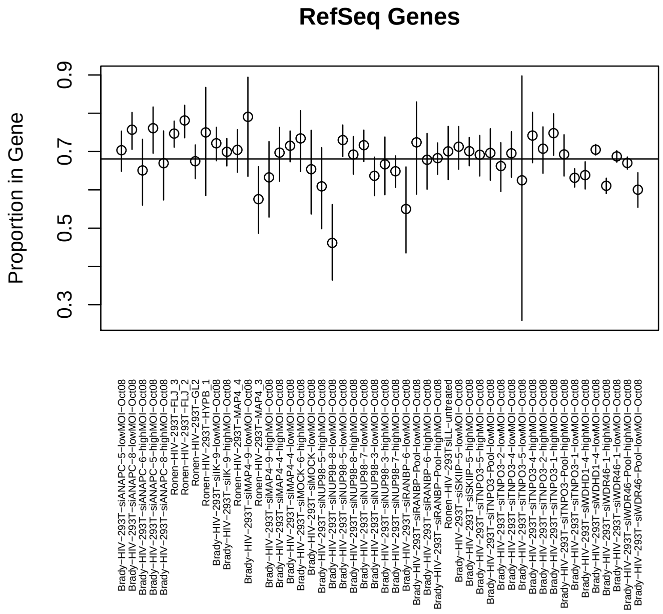

The chisquare statistic for the 2 by 50 table is 230.4, which attains $p = 2.6e-25$. As can be seen there are a number of lines whose confidence interval does not include the overall proportion.

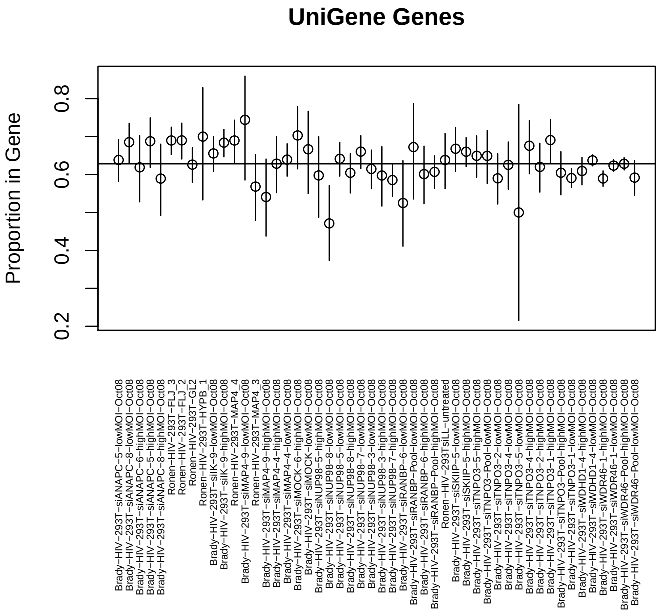

The chisquare statistic for the 2 by 50 table is 121.9, which attains $p = 3.8e-08$ Here, too, there are a number of lines whose confidence interval does not include the overall proportion.

4 Local Sequence Effects

The following plot uses a heatmap to show the Mahalanobis distances between the position specific proportions of oligonucleotides of the 20 flanking bases for each pair of lines. The clustering dendrogram is presented as a heuristic tool for visually organizing those distances.

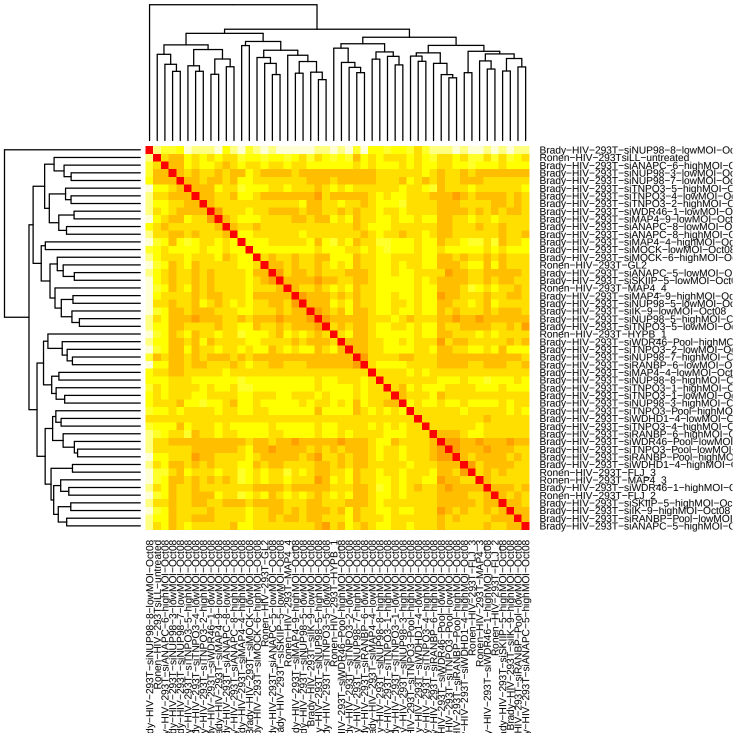

Taking the chisquare distiribution with 60 degrees as th ereference distribution, p-values are computed for each pair of lines. There is 1 line from which 25 or more lines differ at a false discovery rate of 0.01 or less using the q-value criterion [Storey et al., 2004]. This is Brady-HIV-293T-siNUP98-8-lowMOI-Oct08.

The oligonucleotide proportions in the 20 base pair regions surrounding the integration sites are shown in the following figure. The proportions for Brady-HIV-293T-siNUP98-8-lowMOI-Oct08 for each position are compared to those for the other lines using the Pearson chi-square test for the 2 by 4 table. The tick marks at the bottom show locations at which the difference is significant at $p < 0.05$ (chi-square test for the 2 by 4 table) with (thick lines) and without (narrow lines) Bonferronization for 20 comparisons.

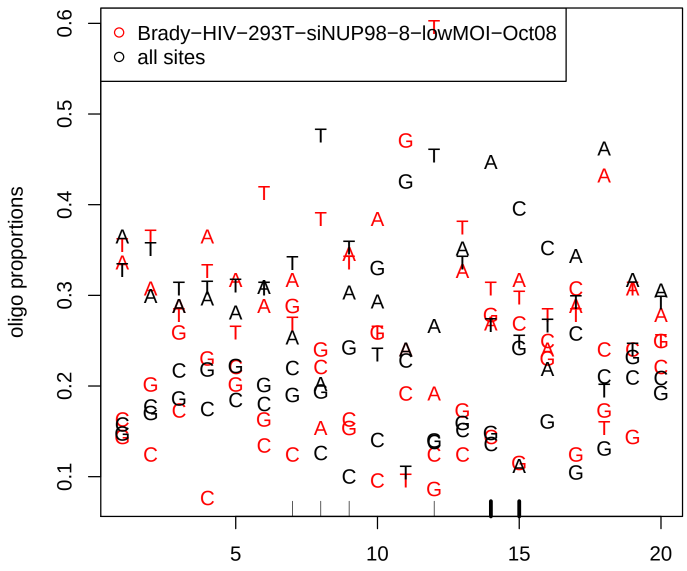

5 GC effects

The following figure shows the Mahalanobis distances between vectors of GC proportions in windows of varying sizes.

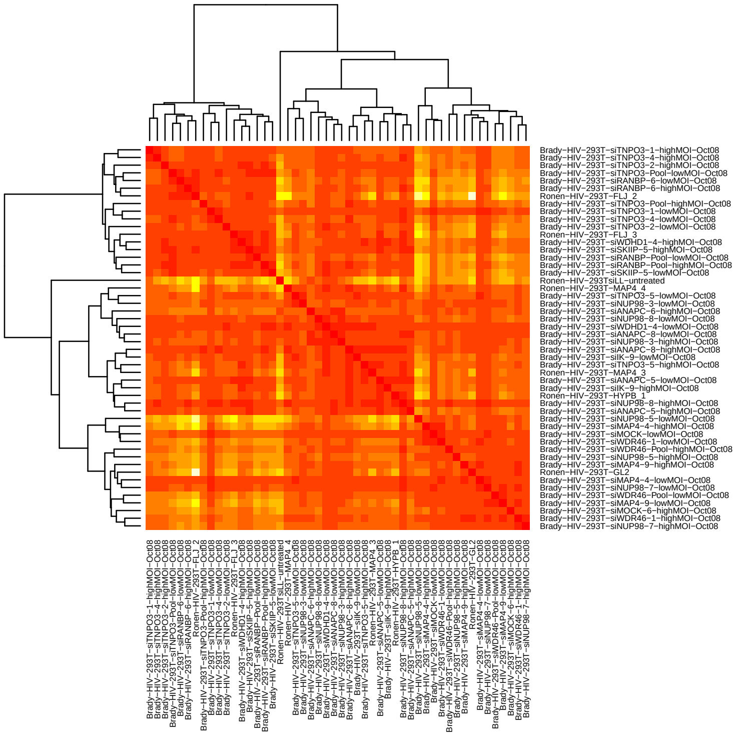

From the cluster diagram, it appears that there are four or more groups according to the GC density in different windows. The following graph shows the patterns of GC richness in those four groups.

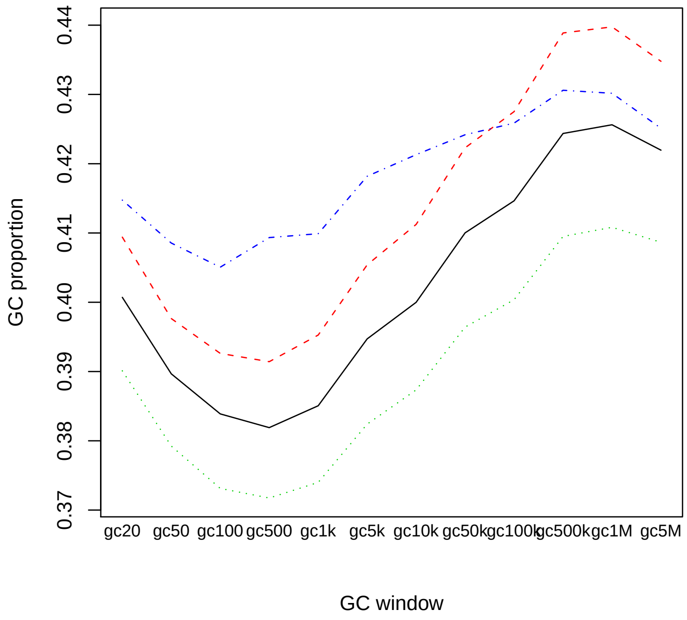

As can be seen, the principal difference among three of the groups is in the overall level of the GC curves, whilst the fourth shows somewhat less GC effect across the range of window widths.

6 RefSeq Density

The following figure shows the pairs of Mahalanobis distances for the density of RefSeq genes in windows are varying widths surrounding the integration site.

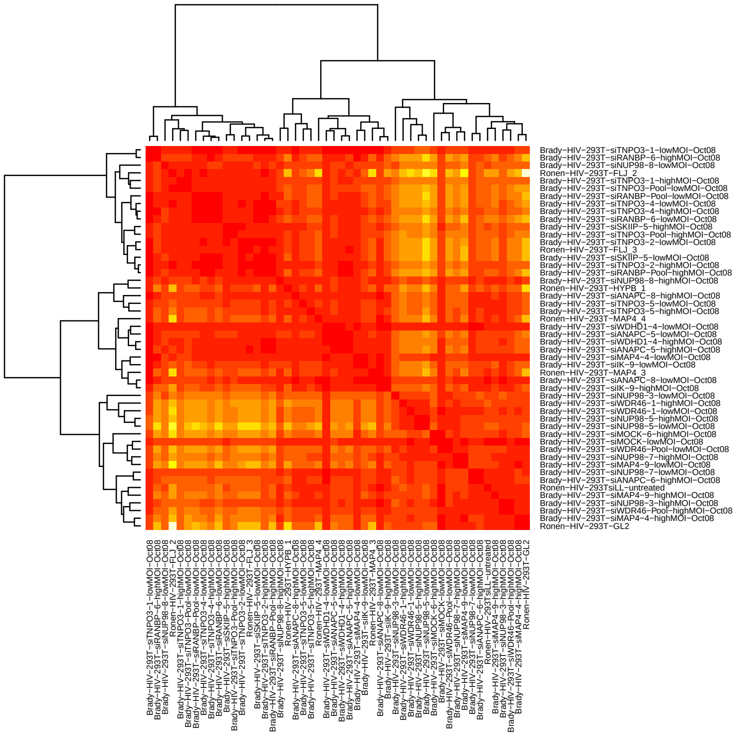
Brady−HIV−293T−siTNPO3−1−lowMOI−Oct08
Brady−HIV−293T−siRANBP−6−highMOI−Oct08
Brady−HIV−293T−siNUP98−8−lowMOI−Oct08
Ronen−HIV−293T−FLJ_2
Brady−HIV−293T−siTNPO3−1−highMOI−Oct08
Brady−HIV−293T−siTNPO3−Pool−lowMOI−Oct08
Brady−HIV−293T−siRANBP−Pool−lowMOI−Oct08
Brady−HIV−293T−siTNPO3−4−lowMOI−Oct08
Brady−HIV−293T−siTNPO3−4−highMOI−Oct08
Brady−HIV−293T−siRANBP−6−lowMOI−Oct08
Brady−HIV−293T−siSKIIP−5−highMOI−Oct08
Brady−HIV−293T−siTNPO3−Pool−highMOI−Oct08
Brady−HIV−293T−siTNPO3−2−lowMOI−Oct08
Ronen−HIV−293T−FLJ_3
Brady−HIV−293T−siSKIIP−5−lowMOI−Oct08
Brady−HIV−293T−siTNPO3−2−highMOI−Oct08
Brady−HIV−293T−siRANBP−Pool−highMOI−Oct08
Brady−HIV−293T−siNUP98−8−highMOI−Oct08
Ronen−HIV−293T−HYPB_1
Brady−HIV−293T−siANAPC−8−highMOI−Oct08
Brady−HIV−293T−siTNPO3−5−lowMOI−Oct08
Brady−HIV−293T−siTNPO3−5−highMOI−Oct08
Ronen−HIV−293T−MAP4_4
Brady−HIV−293T−siWDHD1−4−lowMOI−Oct08
Brady−HIV−293T−siANAPC−5−lowMOI−Oct08
Brady−HIV−293T−siWDHD1−4−highMOI−Oct08
Brady−HIV−293T−siANAPC−5−highMOI−Oct08
Brady−HIV−293T−siMAP4−4−lowMOI−Oct08
Brady−HIV−293T−siIK−9−lowMOI−Oct08
Ronen−HIV−293T−MAP4_3
Brady−HIV−293T−siANAPC−8−lowMOI−Oct08
Brady−HIV−293T−siIK−9−highMOI−Oct08
Brady−HIV−293T−siNUP98−3−lowMOI−Oct08
Brady−HIV−293T−siWDR46−1−highMOI−Oct08
Brady−HIV−293T−siWDR46−1−lowMOI−Oct08
Brady−HIV−293T−siNUP98−5−highMOI−Oct08
Brady−HIV−293T−siNUP98−5−lowMOI−Oct08
Brady−HIV−293T−siMOCK−6−highMOI−Oct08
Brady−HIV−293T−siMOCK−lowMOI−Oct08
Brady−HIV−293T−siWDR46−Pool−lowMOI−Oct08
Brady−HIV−293T−siNUP98−7−highMOI−Oct08
Brady−HIV−293T−siMAP4−9−lowMOI−Oct08
Brady−HIV−293T−siNUP98−7−lowMOI−Oct08
Brady−HIV−293T−siANAPC−6−highMOI−Oct08
Ronen−HIV−293TsiLL−untreated
Brady−HIV−293T−siMAP4−9−highMOI−Oct08
Brady−HIV−293T−siNUP98−3−highMOI−Oct08
Brady−HIV−293T−siWDR46−Pool−highMOI−Oct08
Brady−HIV−293T−siMAP4−4−highMOI−Oct08
Ronen−HIV−293T−GL2

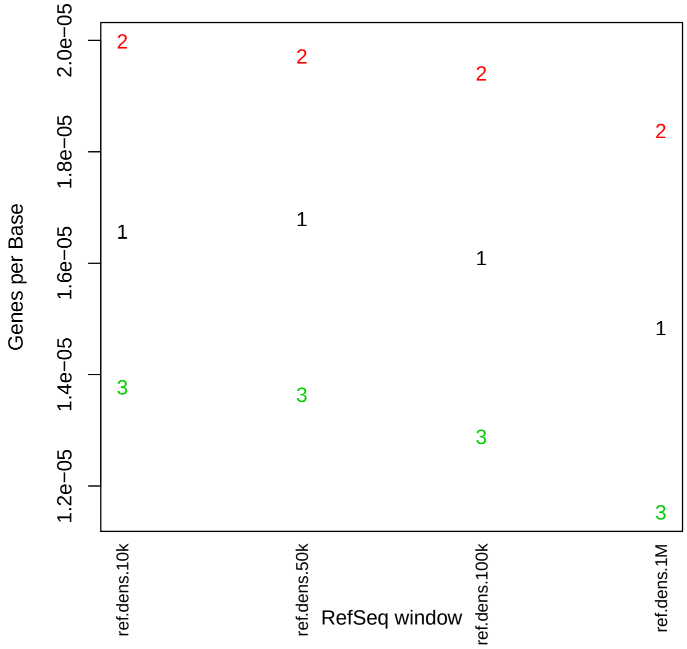

Evidently the principal differences are in the overall frequency of RefSeq genes in the neighborhood of the integration sites.

7 Gene Expression Density

The following figure shows the pairs of Mahalanobis distances for the density of expressed genes in windows are varying widths surrounding the integration site.

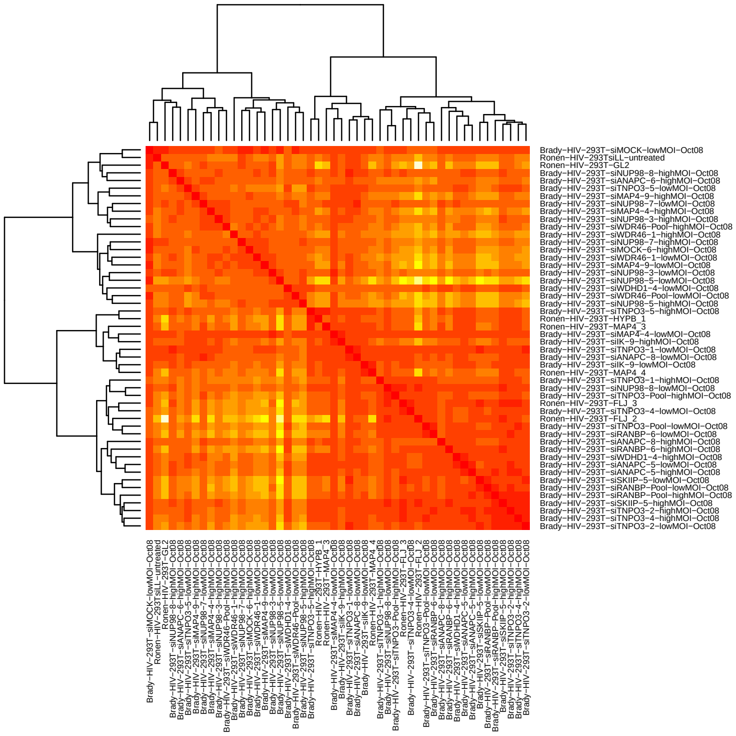

Brady−HIV−293T−siMOCK−lowMOI−Oct08
Ronen−HIV−293TsiLL−untreated
Ronen−HIV−293T−GL2
Brady−HIV−293T−siNUP98−8−highMOI−Oct08
Brady−HIV−293T−siANAPC−6−highMOI−Oct08
Brady−HIV−293T−siTNPO3−5−lowMOI−Oct08
Brady−HIV−293T−siMAP4−9−highMOI−Oct08
Brady−HIV−293T−siNUP98−7−lowMOI−Oct08
Brady−HIV−293T−siMAP4−4−highMOI−Oct08
Brady−HIV−293T−siNUP98−3−highMOI−Oct08
Brady−HIV−293T−siWDR46−Pool−highMOI−Oct08
Brady−HIV−293T−siWDR46−1−highMOI−Oct08
Brady−HIV−293T−siNUP98−7−highMOI−Oct08
Brady−HIV−293T−siMOCK−6−highMOI−Oct08
Brady−HIV−293T−siWDR46−1−lowMOI−Oct08
Brady−HIV−293T−siMAP4−9−lowMOI−Oct08
Brady−HIV−293T−siNUP98−3−lowMOI−Oct08
Brady−HIV−293T−siNUP98−5−lowMOI−Oct08
Brady−HIV−293T−siWDHD1−4−lowMOI−Oct08
Brady−HIV−293T−siWDR46−Pool−lowMOI−Oct08
Brady−HIV−293T−siNUP98−5−highMOI−Oct08
Brady−HIV−293T−siTNPO3−5−highMOI−Oct08
Ronen−HIV−293T−HYPB_1
Ronen−HIV−293T−MAP4_3
Brady−HIV−293T−siMAP4−4−lowMOI−Oct08
Brady−HIV−293T−siIK−9−highMOI−Oct08
Brady−HIV−293T−siTNPO3−1−lowMOI−Oct08
Brady−HIV−293T−siANAPC−8−lowMOI−Oct08
Brady−HIV−293T−siIK−9−lowMOI−Oct08
Ronen−HIV−293T−MAP4_4
Brady−HIV−293T−siTNPO3−1−highMOI−Oct08
Brady−HIV−293T−siNUP98−8−lowMOI−Oct08
Brady−HIV−293T−siTNPO3−Pool−highMOI−Oct08
Ronen−HIV−293T−FLJ_3
Brady−HIV−293T−siTNPO3−4−lowMOI−Oct08
Ronen−HIV−293T−FLJ_2
Brady−HIV−293T−siTNPO3−Pool−lowMOI−Oct08
Brady−HIV−293T−siRANBP−6−lowMOI−Oct08
Brady−HIV−293T−siANAPC−8−highMOI−Oct08
Brady−HIV−293T−siRANBP−6−highMOI−Oct08
Brady−HIV−293T−siWDHD1−4−highMOI−Oct08
Brady−HIV−293T−siANAPC−5−lowMOI−Oct08
Brady−HIV−293T−siANAPC−5−highMOI−Oct08
Brady−HIV−293T−siSKIIP−5−lowMOI−Oct08
Brady−HIV−293T−siRANBP−Pool−lowMOI−Oct08
Brady−HIV−293T−siRANBP−Pool−highMOI−Oct08
Brady−HIV−293T−siSKIIP−5−highMOI−Oct08
Brady−HIV−293T−siTNPO3−2−highMOI−Oct08
Brady−HIV−293T−siTNPO3−4−highMOI−Oct08
Brady−HIV−293T−siTNPO3−2−lowMOI−Oct08

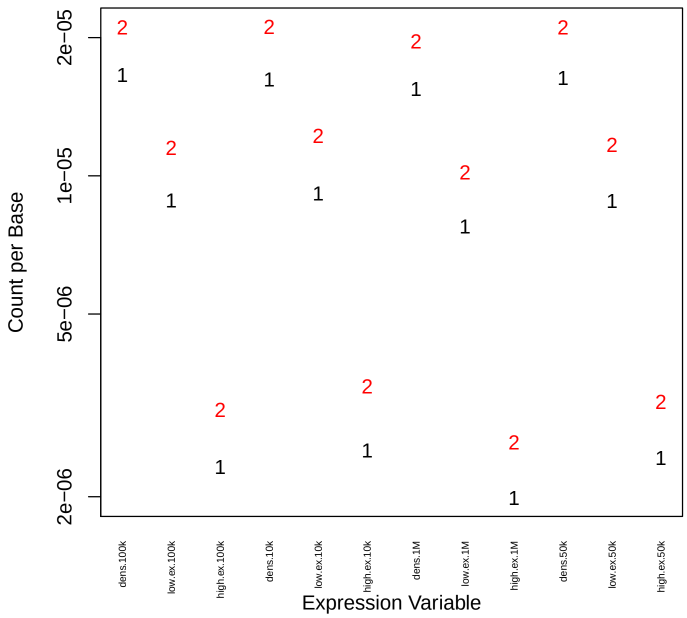

Evidently, the differences showing up in each of the variables is of the same magnitude when viewed on a logarithmic scale.

8 CpG Islands and Density

The following figure shows the pairs of Mahalanobis distances for the presence or count of CpG islands in windows are varying widths surrounding the integration site.

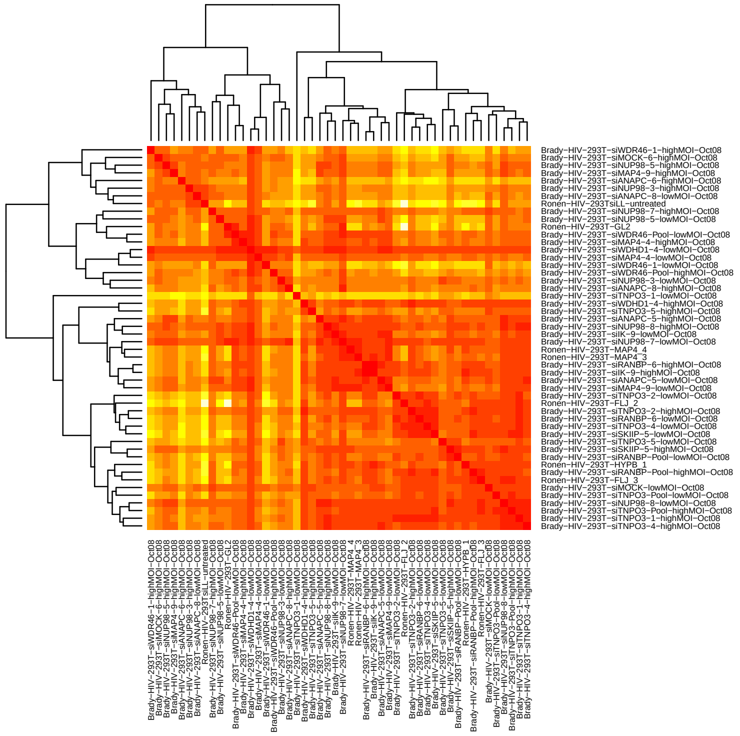
Brady−HIV−293T−siWDR46−1−highMOI−Oct08
Brady−HIV−293T−siMOCK−6−highMOI−Oct08
Brady−HIV−293T−siNUP98−5−highMOI−Oct08
Brady−HIV−293T−siMAP4−9−highMOI−Oct08
Brady−HIV−293T−siANAPC−6−highMOI−Oct08
Brady−HIV−293T−siNUP98−3−highMOI−Oct08
Brady−HIV−293T−siANAPC−8−lowMOI−Oct08
Ronen−HIV−293TsiLL−untreated
Brady−HIV−293T−siNUP98−7−highMOI−Oct08
Brady−HIV−293T−siNUP98−5−lowMOI−Oct08
Ronen−HIV−293T−GL2
Brady−HIV−293T−siWDR46−Pool−lowMOI−Oct08
Brady−HIV−293T−siMAP4−4−highMOI−Oct08
Brady−HIV−293T−siWDHD1−4−lowMOI−Oct08
Brady−HIV−293T−siMAP4−4−lowMOI−Oct08
Brady−HIV−293T−siWDR46−1−lowMOI−Oct08
Brady−HIV−293T−siWDR46−Pool−highMOI−Oct08
Brady−HIV−293T−siNUP98−3−lowMOI−Oct08
Brady−HIV−293T−siANAPC−8−highMOI−Oct08
Brady−HIV−293T−siTNPO3−1−lowMOI−Oct08
Brady−HIV−293T−siWDHD1−4−highMOI−Oct08
Brady−HIV−293T−siTNPO3−5−highMOI−Oct08
Brady−HIV−293T−siANAPC−5−highMOI−Oct08
Brady−HIV−293T−siNUP98−8−highMOI−Oct08
Brady−HIV−293T−siIK−9−lowMOI−Oct08
Brady−HIV−293T−siNUP98−7−lowMOI−Oct08
Ronen−HIV−293T−MAP4_4
Ronen−HIV−293T−MAP4_3
Brady−HIV−293T−siRANBP−6−highMOI−Oct08
Brady−HIV−293T−siIK−9−highMOI−Oct08
Brady−HIV−293T−siANAPC−5−lowMOI−Oct08
Brady−HIV−293T−siMAP4−9−lowMOI−Oct08
Brady−HIV−293T−siTNPO3−2−lowMOI−Oct08
Ronen−HIV−293T−FLJ_2
Brady−HIV−293T−siTNPO3−2−highMOI−Oct08
Brady−HIV−293T−siRANBP−6−lowMOI−Oct08
Brady−HIV−293T−siTNPO3−4−lowMOI−Oct08
Brady−HIV−293T−siSKIIP−5−lowMOI−Oct08
Brady−HIV−293T−siTNPO3−5−lowMOI−Oct08
Brady−HIV−293T−siSKIIP−5−highMOI−Oct08
Brady−HIV−293T−siRANBP−Pool−lowMOI−Oct08
Ronen−HIV−293T−HYPB_1
Brady−HIV−293T−siRANBP−Pool−highMOI−Oct08
Ronen−HIV−293T−FLJ_3
Brady−HIV−293T−siMOCK−lowMOI−Oct08
Brady−HIV−293T−siTNPO3−Pool−lowMOI−Oct08
Brady−HIV−293T−siNUP98−8−lowMOI−Oct08
Brady−HIV−293T−siTNPO3−Pool−highMOI−Oct08
Brady−HIV−293T−siTNPO3−1−highMOI−Oct08
Brady−HIV−293T−siTNPO3−4−highMOI−Oct08

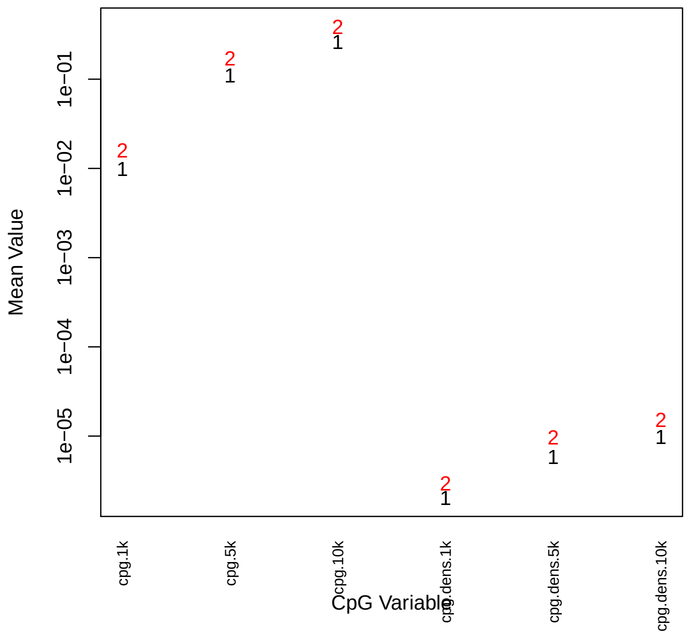

The separation for each of the variables appears to be roughly the same when viewed on the logarithmic scale.

9 OncoGene Neighborhood

The following figure shows the pairs of Mahalanobis distances for the presence of oncogenes in windows are varying widths surrounding the integration site.

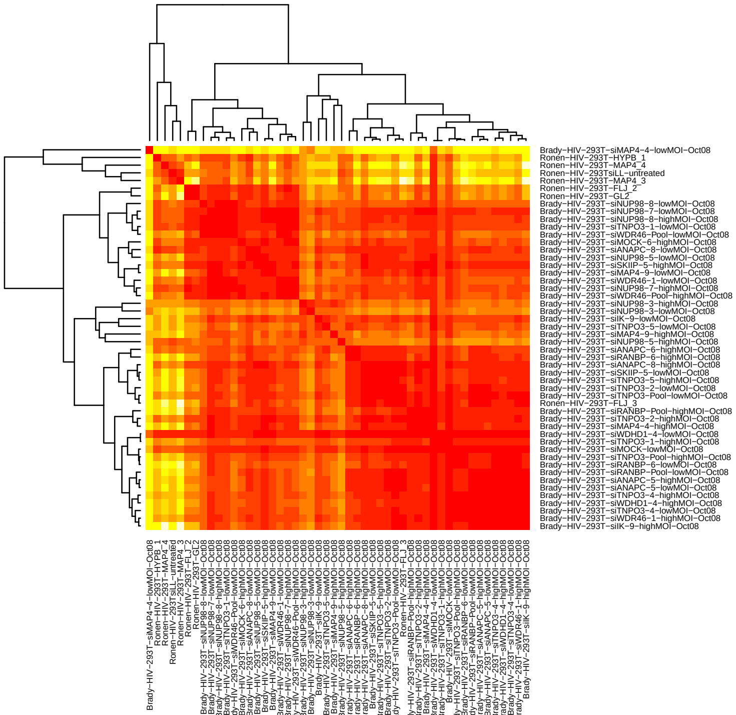
Brady−HIV−293T−siMAP4−4−lowMOI−Oct08
Ronen−HIV−293T−HYPB_1
Ronen−HIV−293T−MAP4_4
Ronen−HIV−293TsiLL−untreated
Ronen−HIV−293T−MAP4_3
Ronen−HIV−293T−FLJ_2
Ronen−HIV−293T−GL2
Brady−HIV−293T−siNUP98−8−lowMOI−Oct08
Brady−HIV−293T−siNUP98−7−lowMOI−Oct08
Brady−HIV−293T−siNUP98−8−highMOI−Oct08
Brady−HIV−293T−siTNPO3−1−lowMOI−Oct08
Brady−HIV−293T−siWDR46−Pool−lowMOI−Oct08
Brady−HIV−293T−siMOCK−6−highMOI−Oct08
Brady−HIV−293T−siANAPC−8−lowMOI−Oct08
Brady−HIV−293T−siNUP98−5−lowMOI−Oct08
Brady−HIV−293T−siSKIIP−5−highMOI−Oct08
Brady−HIV−293T−siMAP4−9−lowMOI−Oct08
Brady−HIV−293T−siWDR46−1−lowMOI−Oct08
Brady−HIV−293T−siNUP98−7−highMOI−Oct08
Brady−HIV−293T−siWDR46−Pool−highMOI−Oct08
Brady−HIV−293T−siNUP98−3−highMOI−Oct08
Brady−HIV−293T−siNUP98−3−lowMOI−Oct08
Brady−HIV−293T−siIK−9−lowMOI−Oct08
Brady−HIV−293T−siTNPO3−5−lowMOI−Oct08
Brady−HIV−293T−siMAP4−9−highMOI−Oct08
Brady−HIV−293T−siNUP98−5−highMOI−Oct08
Brady−HIV−293T−siANAPC−6−highMOI−Oct08
Brady−HIV−293T−siRANBP−6−highMOI−Oct08
Brady−HIV−293T−siANAPC−8−highMOI−Oct08
Brady−HIV−293T−siSKIIP−5−lowMOI−Oct08
Brady−HIV−293T−siTNPO3−5−highMOI−Oct08
Brady−HIV−293T−siTNPO3−2−lowMOI−Oct08
Brady−HIV−293T−siTNPO3−Pool−lowMOI−Oct08
Ronen−HIV−293T−FLJ_3
Brady−HIV−293T−siRANBP−Pool−highMOI−Oct08
Brady−HIV−293T−siTNPO3−2−highMOI−Oct08
Brady−HIV−293T−siMAP4−4−highMOI−Oct08
Brady−HIV−293T−siWDHD1−4−lowMOI−Oct08
Brady−HIV−293T−siTNPO3−1−highMOI−Oct08
Brady−HIV−293T−siMOCK−lowMOI−Oct08
Brady−HIV−293T−siTNPO3−Pool−highMOI−Oct08
Brady−HIV−293T−siRANBP−6−lowMOI−Oct08
Brady−HIV−293T−siRANBP−Pool−lowMOI−Oct08
Brady−HIV−293T−siANAPC−5−highMOI−Oct08
Brady−HIV−293T−siANAPC−5−lowMOI−Oct08
Brady−HIV−293T−siTNPO3−4−highMOI−Oct08
Brady−HIV−293T−siWDHD1−4−highMOI−Oct08
Brady−HIV−293T−siTNPO3−4−lowMOI−Oct08
Brady−HIV−293T−siWDR46−1−highMOI−Oct08
Brady−HIV−293T−siIK−9−highMOI−Oct08

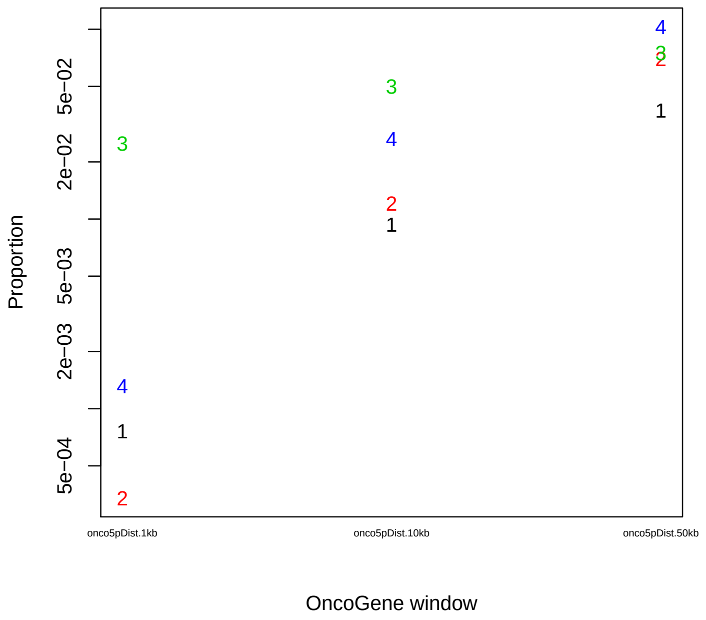

Here there appear to be four groups of sites defined by the frequency of oncogenes in thier neighborhoods. However, the data are exceedingly sparse, and none of the apparent differences is even nominally significant.

10 All Variables

The following figure shows the pairs of Mahalanobis distances for the all of the above variables considered simultaneously.

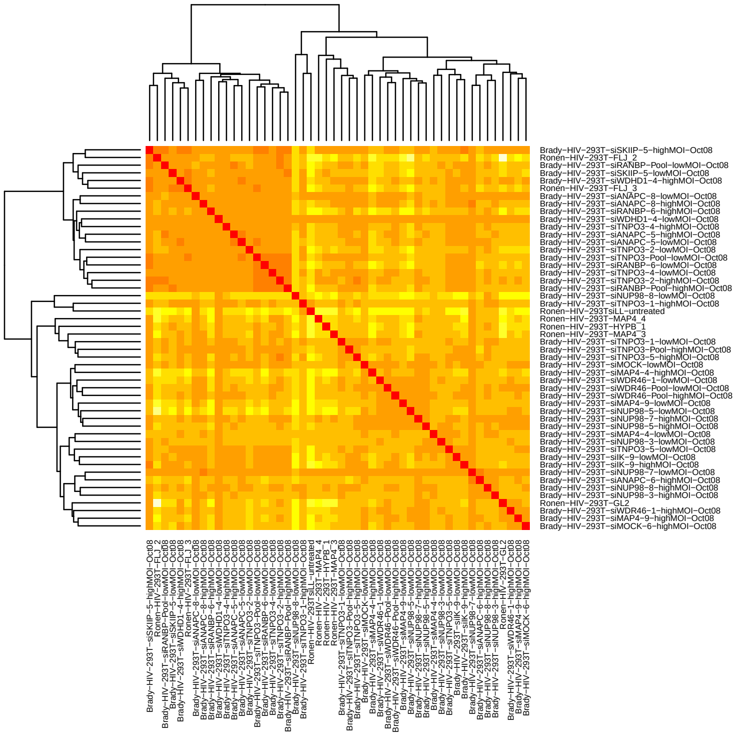

There appear to be two distinct groups. The following graphic shows the differences in the average levels of the variables after they have been normalized to have mean zero and unit variance (which makes it possible to display all of them simultaneously). As can be seen, the biggest differences are due to regional variations in GC density and gene density.

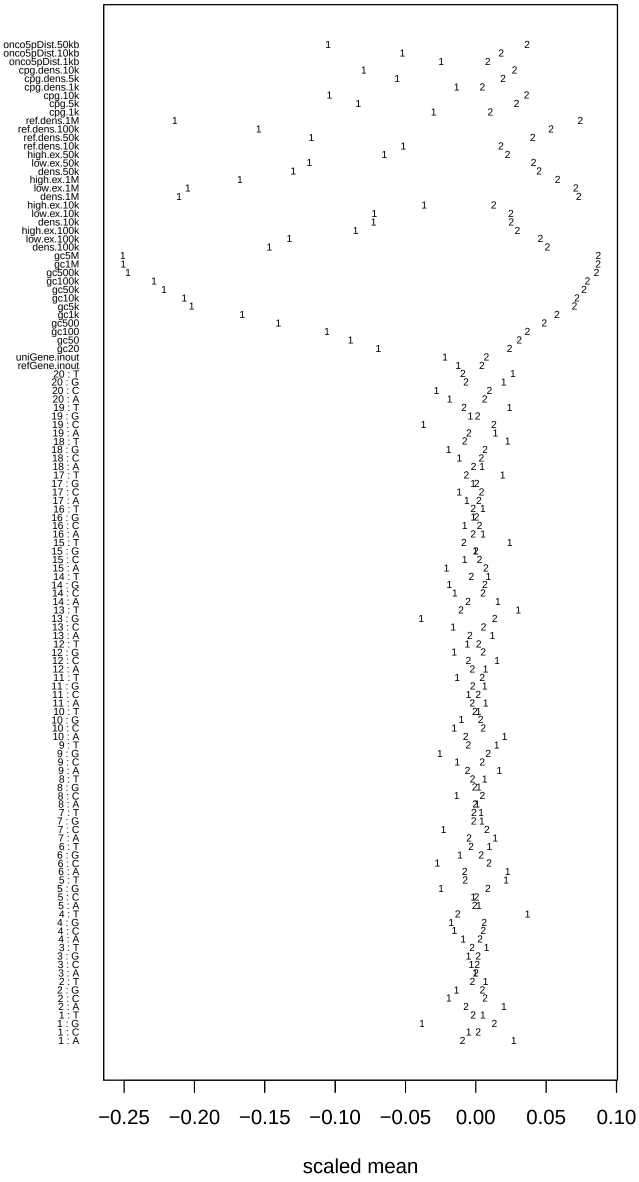

11 Predicted Intensity

Here the predicted integration intensity is used to assess the distances between the lines. For each line, a fitted model that predicts integration intensity is applied to a common set of genomic sites. In the figure that follows, the Euclidean

distance between the collections of predicted intensities is displayed and used as a basis for constructing the clustering diagram. The actual model used is the conditional logit model implemented in the `survival` R package. The variables used in the model for each line are determined by the sequence of variables entered in an ordinary logistic model subjected to an L1 penalty using the `glmnet` R package. The target is to fit all of the variables selected using the default settings of the `glmnet` function using the conditional logistic model with an L2 penalty whose target is to penalize to achieve an effective number of degrees of freedom that is half of the number of variables included. As it turns out, this is not always achievable due to numerical instabilities. The approach used to finding a feasible fit is to enter variables in the same order as the logistic fit does with a decreasing L1 penalty and exclude any variable that leads to numerical instabilities.

The estimated distance between predicted intensity of two lines is inflated by the variation in the estimates of intensity. In fact, the line with the fewest integration sites seems to stand apart, which may only reflect the difficulty in properly characterizing the genomic site preference in that line. To avoid being mislead by excessive variation, only lines in which there are 200 or more integration sites are studied below.

Here the Euclidean distance between the logarithms of the integration intensities is studied (after centering the log intensities at zero).

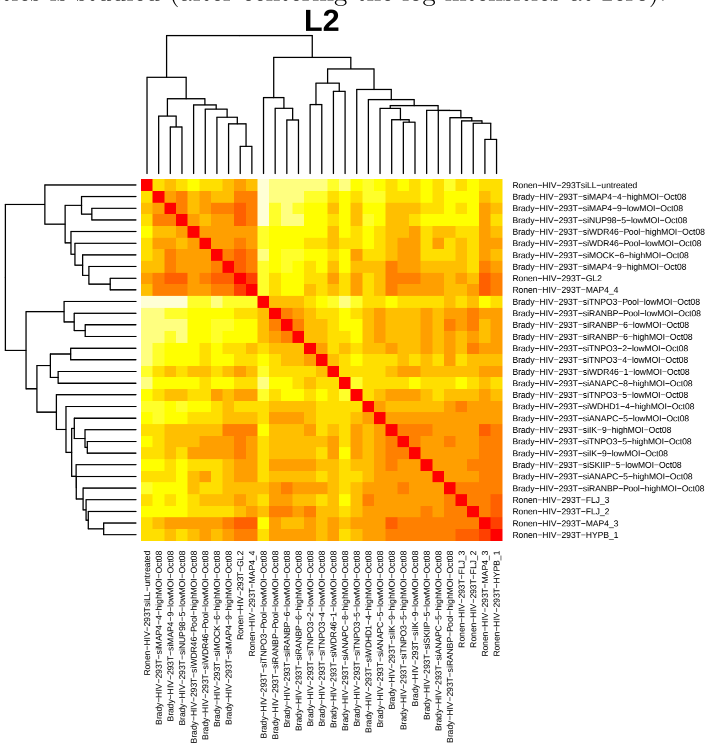

The following figure uses the same data, but both centers and scales the

predictions. The Euclidean distance here is just $1-rij$, where r_{ij} is the Pearson correlation of the predicted intensities for lines i and j.

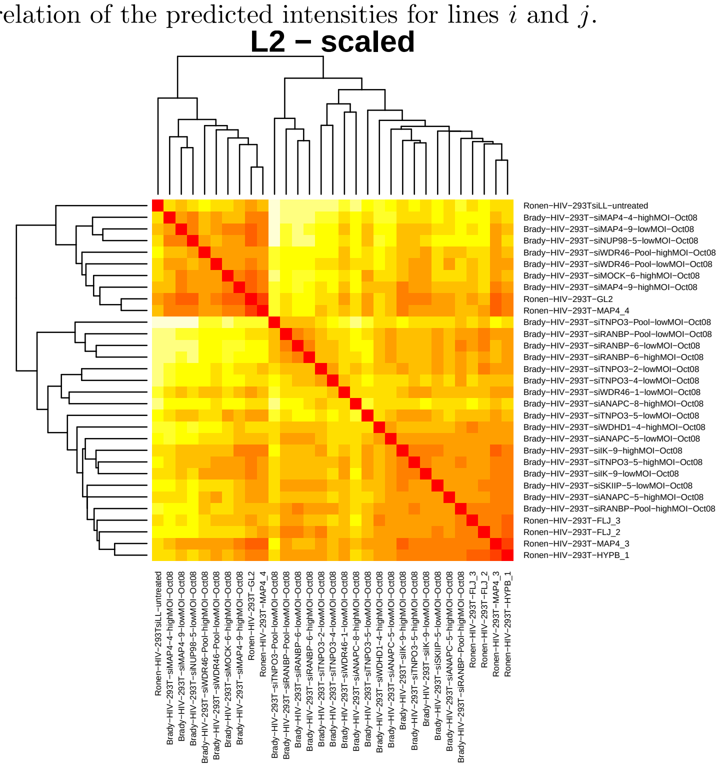

Here the intensities are estimated using Bayes Model Averaging of conditional logit models as implemented in the `BMA` R package. The subset of variables used in each line is that selected by the L1 penalized logistic regression described above. Sometimes, numerical instabilities arose, which were eliminated by removing candidate regressors that showed linear dependencies on the other regressors. As before the log intensities are centered.

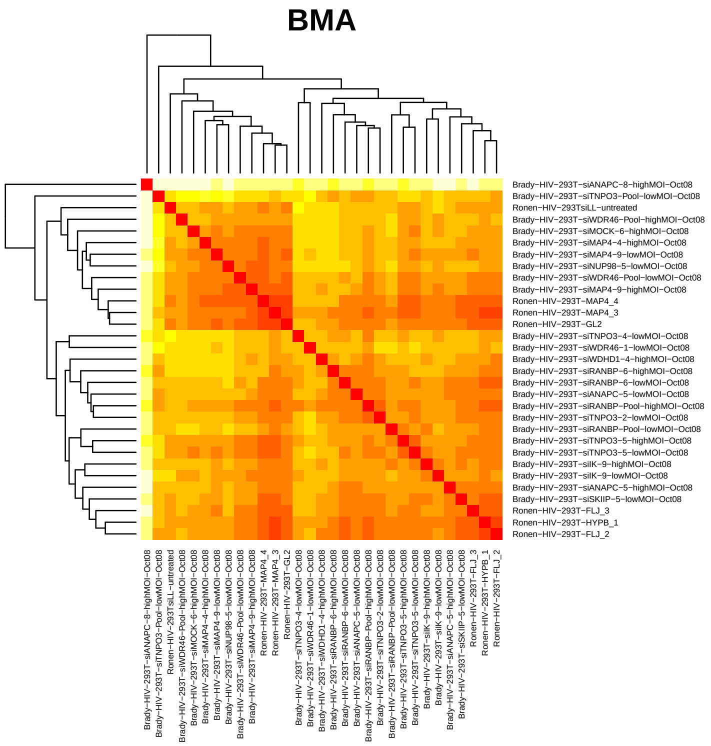

And here is the result when the log intensities are both centered and scaled.

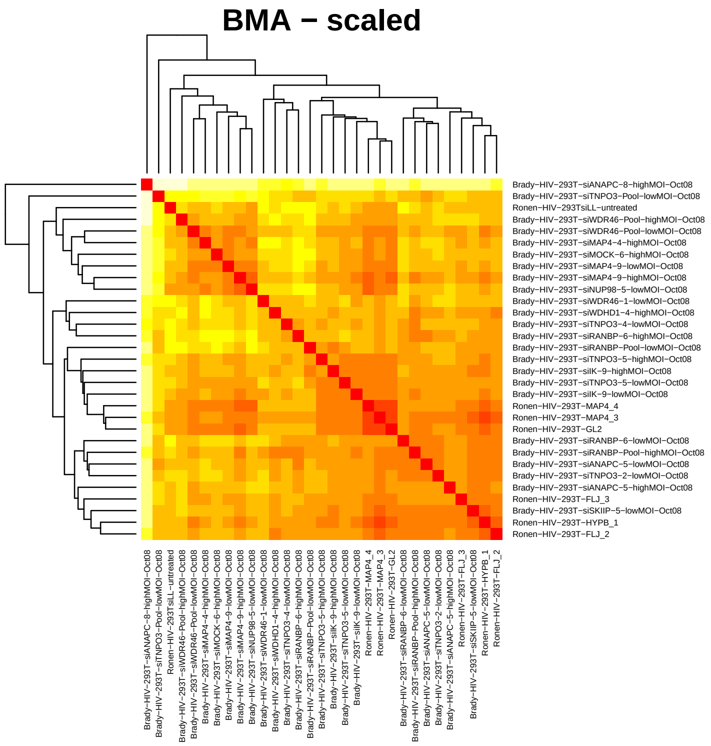

In each of the above heatmaps, the lines corresponding to the 4 categories highest in the respect dendrogram were identified. The table below shows how well they agree.

L2	BMA	L2.scaled	BMA.scaled	Freq
1	1	1	1	12
1	3	1	1	1
2	1	2	1	3
3	3	3	1	2
4	1	4	1	3
2	2	2	2	1
3	3	3	3	8
4	4	4	4	1

As can be seen, there is substantial agreement among the categories. The figure below shows the means of individual variables corresponding to four categories obtained from the L2 penalized conditional logit fit.

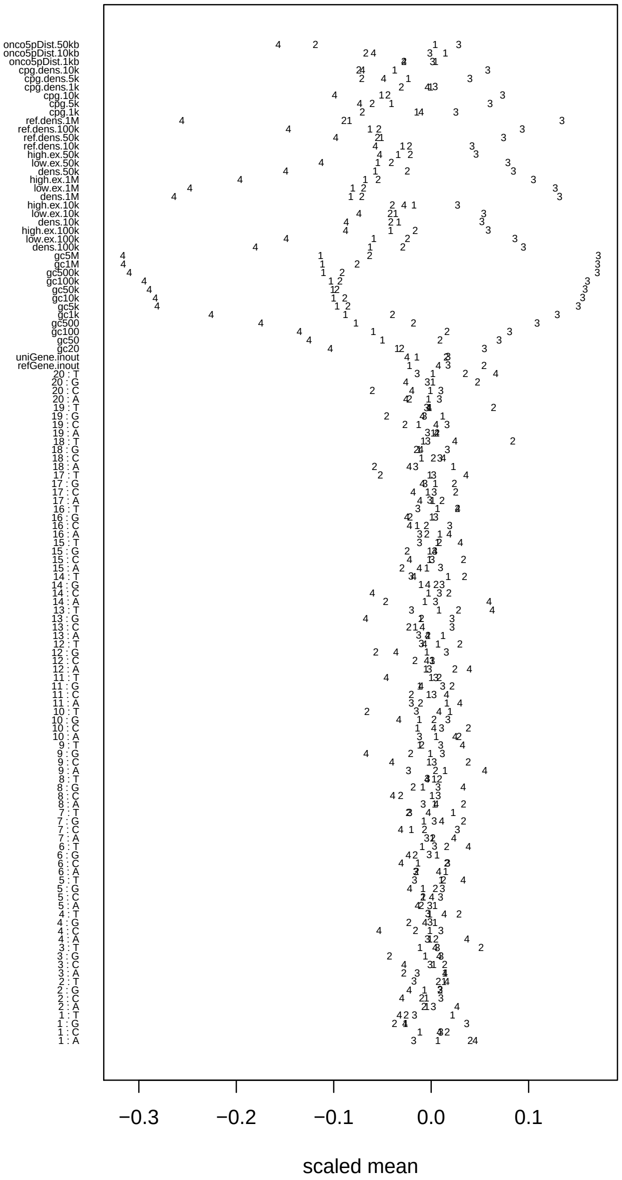

The identity of the lines according to the category in the dendrograms shown above is given here:

	L2	L2.scaled	BMA	BMA.scaled
Brady-HIV-293T-siANAPC-5-highMOI-Oct08	1	1	1	1
Brady-HIV-293T-siANAPC-5-lowMOI-Oct08	1	1	1	1
Brady-HIV-293T-siANAPC-8-highMOI-Oct08	2	2	2	2
Brady-HIV-293T-siIK-9-highMOI-Oct08	1	1	1	1
Brady-HIV-293T-siIK-9-lowMOI-Oct08	1	1	1	1
Brady-HIV-293T-siMAP4-4-highMOI-Oct08	3	3	3	3
Brady-HIV-293T-siMAP4-9-highMOI-Oct08	3	3	3	3
Brady-HIV-293T-siMAP4-9-lowMOI-Oct08	3	3	3	3

Brady-HIV-293T-siMOCK-6-highMOI-Oct08	3	3	3	3
Brady-HIV-293T-siNUP98-5-lowMOI-Oct08	3	3	3	3
Brady-HIV-293T-siRANBP-6-highMOI-Oct08	4	4	1	1
Brady-HIV-293T-siRANBP-6-lowMOI-Oct08	4	4	1	1
Brady-HIV-293T-siRANBP-Pool-highMOI-Oct08	1	1	1	1
Brady-HIV-293T-siRANBP-Pool-lowMOI-Oct08	4	4	1	1
Brady-HIV-293T-siSKIIP-5-lowMOI-Oct08	1	1	1	1
Brady-HIV-293T-siTNPO3-2-lowMOI-Oct08	2	2	1	1
Brady-HIV-293T-siTNPO3-4-lowMOI-Oct08	2	2	1	1
Brady-HIV-293T-siTNPO3-5-highMOI-Oct08	1	1	1	1
Brady-HIV-293T-siTNPO3-5-lowMOI-Oct08	1	1	1	1
Brady-HIV-293T-siTNPO3-Pool-lowMOI-Oct08	4	4	4	4
Brady-HIV-293T-siWDHD1-4-highMOI-Oct08	1	1	1	1
Brady-HIV-293T-siWDR46-1-lowMOI-Oct08	2	2	1	1
Brady-HIV-293T-siWDR46-Pool-highMOI-Oct08	3	3	3	3
Brady-HIV-293T-siWDR46-Pool-lowMOI-Oct08	3	3	3	3
Ronen-HIV-293T-FLJ_2	1	1	1	1
Ronen-HIV-293T-FLJ_3	1	1	1	1
Ronen-HIV-293T-GL2	3	3	3	1
Ronen-HIV-293T-HYPB_1	1	1	1	1
Ronen-HIV-293T-MAP4_3	1	1	3	1
Ronen-HIV-293T-MAP4_4	3	3	3	1
Ronen-HIV-293TsiLL-untreated	3	3	3	3

12 Software Used

The computations in this report were carried out using [R Development Core Team, 2008]. The report was generated using the `Sweave` function. Packages `glmnet` [Friedman et al., 2008], `survival`[Therneau and original R port by Thomas Lumley, 2009] and `BMA`[Raftery et al., 2009] were used. The `BMA` package was modified to allow stratified Cox models and to better report failures due to linear dependencies amongst regressors.

References

[Berry et al., 2006] Berry, C., Hannenhalli, S., Leipzig, J., and Bushman, F. (2006). Selection of target sites for mobile DNA integration in the human genome. *PLoS Comput Biol*, 2(11):e157.

[Friedman et al., 2008] Friedman, J., Hastie, T., and Tibshirani, R. (2008). *glmnet: Lasso and elastic-net regularized generalized linear models.* R package version 1.1.

[R Development Core Team, 2008] R Development Core Team (2008). *R: A Language and Environment for Statistical Computing.* R Foundation for Statistical Computing, Vienna, Austria. ISBN 3-900051-07-0.

[Raftery et al., 2009] Raftery, A., Hoeting, J., Volinsky, C., Painter, I., and Yeung, K. Y. (2009). *BMA: Bayesian Model Averaging.* R package version 3.10.

[Storey et al., 2004] Storey, J., Taylor, J., and Siegmund, D. (2004). Strong control, conservative point estimation and simultaneous conservative consistency of false discovery rates: a unified approach. *Journal of the Royal Statistical Society. Series B, Statistical Methodology*, pages 187–205.

[Therneau and original R port by Thomas Lumley, 2009] Therneau, T. and original R port by Thomas Lumley (2009). *survival: Survival analysis, including penalised likelihood.* R package version 2.35-4.
